# Towards a phylogenetic classification of Leptothecata (Cnidaria, Hydrozoa)

**DOI:** 10.1038/srep18075

**Published:** 2016-01-29

**Authors:** Maximiliano M. Maronna, Thaís P. Miranda, Álvaro L. Peña Cantero, Marcos S. Barbeitos, Antonio C. Marques

**Affiliations:** 1 Departamento de Zoologia, Instituto de Biociências, Universidade de São Paulo Rua do Matão Trav. 14, 101, 05508-090, São Paulo, Brazil; 2 Instituto Cavanilles de Biodiversidad y Biología Evolutiva, Departamento de Zoología Universidad de Valencia, Valencia, Spain; 3 Departamento de Zoologia, Caixa Postal 19020, Universidade Federal do Paraná, 81531-990, Curitiba, PR, Brazil; 4 Centro de Biologia Marinha, Universidade de São Paulo, São Sebastião, Brazil

## Abstract

Leptothecata are hydrozoans whose hydranths are covered by perisarc and gonophores and whose medusae bear gonads on their radial canals. They develop complex polypoid colonies and exhibit considerable morphological variation among species with respect to growth, defensive structures and mode of development. For instance, several lineages within this order have lost the medusa stage. Depending on the author, traditional taxonomy in hydrozoans may be either polyp- or medusa-oriented. Therefore, the absence of the latter stage in some lineages may lead to very different classification schemes. Molecular data have proved useful in elucidating this taxonomic challenge. We analyzed a super matrix of new and published rRNA gene sequences (16S, 18S and 28S), employing newly proposed methods to measure branch support and improve phylogenetic signal. Our analysis recovered new clades not recognized by traditional taxonomy and corroborated some recently proposed taxa. We offer a thorough taxonomic revision of the Leptothecata, erecting new orders, suborders, infraorders and families. We also discuss the origination and diversification dynamics of the group from a macroevolutionary perspective.

Leptothecata Cornelius, 1992 is the most speciose clade within Medusozoa Petersen, 1979, with approximately 2,000 nominal species described, corresponding to more than half of the richness of Medusozoa^1^,^2^,^3^. Historically, its taxonomy has been based on diverse morphological characters of the benthic (polyps and colonies) and planktonic stages (medusae; see reviews in Cornelius^4^,^5^). The original diagnosis of Thecata was remarkably based on exoskeletal characters of polyps, mainly the hydrotheca and the gonotheca: “Polypi surrounded by a membranaceous tube, covering the subdivisions of their compound body” (Fleming^6^, p. 505). The metagenetic life cycle in Medusozoa was described thereafter^7^, with consequences for the taxonomic arrangement due to a dual classification system, one for polyps and another for medusae.

Subsequently, Thecaphora was proposed by Hincks (1868)^8^ as “Hydroida furnished with thecae” (p. lxvii) and “The chitinous receptacle in which the polypites are lodged in one of the Hydroid suborders (*Thecaphora*)” (p. iii), along with the description of several suprageneric taxa (e.g., Campanulinidae, Haleciidae and Lafoeidae). The classification was also based on skeletal characters of the polyps and, when present, on the medusa stage. Classification problems were recognized for some groups, including uncertainties due to the lack of information on the life cycle of the polyp and the medusa stages (Allman^9^, Hincks^8^, Cornelius^10^, Calder^11^). In 1871, Allman^12^ proposed the taxon Calyptoblastea based on characters of the skeleton surrounding the polyp stage and variations in the life cycle: “Calyptoblastic ([...] covered; [...] bud). The condition of a hydroid when an external protective receptacle (hydrotheca or gonangium) invests either the nutritive or generative buds. CALYPTOBLASTEA, the name of one of the sub-orders of HYDROIDA” (1871, p. xvii, all capitals in the original)^12^.

Based on the medusa stage, Haeckel^13^ proposed the taxon Leptomedusae and several other suprageneric taxa (e.g., Eirenidae, Mitrocomidae). This proposal strengthened a parallel classification scheme, with some studies based only on the polyp stage, and others only on the medusa stage.

Broch^14^ split the leptothecates into Conica (species with conical hypostome and without pre-gastric cavity) and Proboscoida (“Proboscoidea” p. 224, species with globular hypostome and pre-gastric cavity). With few exceptions (*e.g*., Rees^15^), this dual classification predominated until the second half of the 20^th^ century, when Naumov^16^,^17^ aimed at an integrative approach^18^, highlighting that “…to create such a unified classification of medusae and polyps, accurate data is needed on the individuals of different generations indicating that they belong to the same species…” (Naumov^17^, p. 94). This proposal, along with Hincks^8^,^19^,^20^,^21^, Allman^9^,^12^, Morton^22^, and Rees^15^, reinforced a classification of Hydrozoa that was maintained until the end of the 20^th^ century (*viz.*, Millard^23^; Cornelius^24^; Bouillon^25^,^26^; Petersen^27^). The main taxonomic change along this period was the fusion and definition of names based on polyps and medusae, although these decisions were inconsistent with the classification of Hydrozoa at the time^25^,^26^ (summary in Table 6 of Bouillon^28^, p. 266). Later, this classification was intensively criticized and only partially accepted (Cornelius^4^,^5^; Bouillon & Boero^29^; Bouillon *et al.*^30^).

Finally, the name Leptothecata Cornelius, 1992 (Cornelius^31^, p. 246) was proposed as an alternative to synthesize the taxa “Leptomedusae” + “Thecatae” as a counterpart to Anthoathecata, “Anthomedusae” + “Athecatae”. At the same time, other phylogenetic hypotheses for Hydrozoa were proposed, based on morphological^32^,^33^ and molecular^34^ analyses, as well as important studies challenging the status of many taxa, including Leptothecata itself. In the past decade, identification and corroboration of major hydrozoan lineages were accomplished, although the main relationships among them were not conclusive (e.g., Hydroidolina^35^; Trachylina^36^,^37^,^38^).

Leptothecata has always been considered a monophyletic taxon^32^,^34^,^35^,^39^. Nevertheless, additional hypotheses for internal relationships based on molecular (Campanulariidae Johnston, 1836^40^; Plumularioidea McCrady, 1859^41^,^42^; Lafoeidae A. Agassiz, 1865 and Hebellidae Fraser, 1912^43^) and morphological analyses (Lafoeidae and Hebellidae^44^) challenged the monophyly of several of those taxa. A landmark leptothecate phylogeny incorporated important new taxa and recovered two main clades, Statocysta and Macrocolonia^45^. We assessed, reviewed and discussed phylogenetic hypotheses from these recent studies, including those in which new family-level groups were recognized (e.g., Schizotrichidae^46^).

Clearly, the existence of the polyp and the medusa stages creates complex issues for taxonomic interpretation. The current phylogenetic context obtained from molecular analyses points to obvious conflicts concerning classification and the current interpretation of macroevolutionary patterns, such as the evolution of the polyp skeleton in the Proboscoida clade and medusa traits in Statocysta. Thus, unresolved questions remain for both ecology and evolution of Leptothecata and demand a more thorough analysis, considering phylogeny, classification and taxonomy. Therefore the goals of this study are (1) to propose a phylogenetic hypothesis using molecular data from the largest set of Leptothecata sequences assembled so far; (2) to compare this hypothesis with other hypotheses proposed in the last 20 years; (3) to propose a new taxonomic classification for the order and (4) to briefly discuss some macroevolutionary considerations about the Leptothecata, mainly considering the large-scale phylogenetic patterns in morphology and biology that have become evident as a consequence of the proposed hypothesis.

## Results

**Figure 1:**
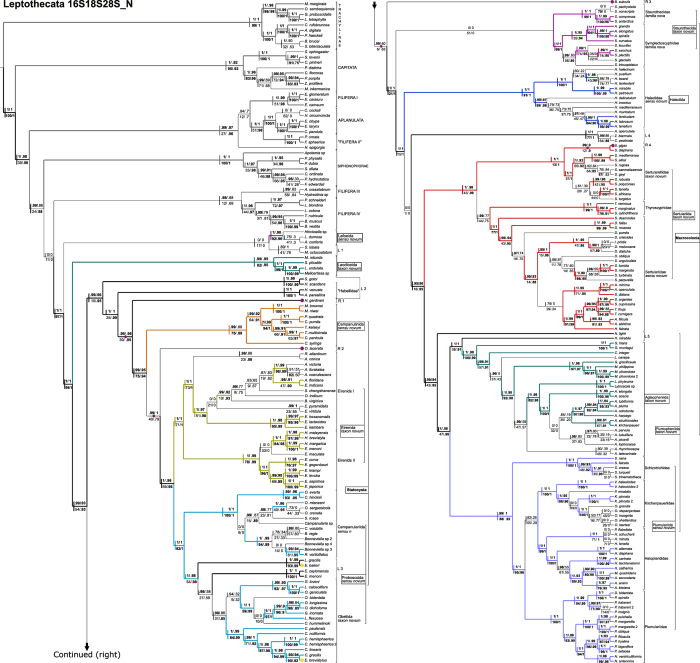
Cladogram of the phylogenetic analysis of Leptothecata with minimum filtering (matrix 16S18S28S_N). Support values are integer numbers or decimals (no leading zeros). Parametric support above the branch (aBAYES/aLRT), non-parametic support below the branch (BS/SH-aLRT); values in plain text indicate non-significant support (aBAYES < 0.95; aLRT < 0.9; BS < 75%; SH-aLRT < 0.85); significant support values are in bold face (aBAYES ≥ 0.95; aLRT ≥ 0.9; BS ≥ 75%; SH-aLRT ≥ 0.85). Colors were used when support is significant for at least 3 out of 4 methods and fine black lines for those with low support (two or fewer significant values). The bars above branches indicate clades found in the topology obtained from the Leptothecata 16S18S28S matrix; bars beneath the branches indicate the same for 16S18S28S_Nrw4. To the right of the description of major clades are details on the newly proposed groups, emphasizing suborders and orders (black box). Purple dots indicate unstable terminals (rogue taxa) and yellow dots were likely misidentified. Rogue taxa were not considered in the evaluation of the clades in other analyses, and together with the ones marked by yellow dots, are considered *incertae sedis* in the proposed taxonomy. Basic taxonomy (species and genera) from the WoRMS database.

**Figure 2:**
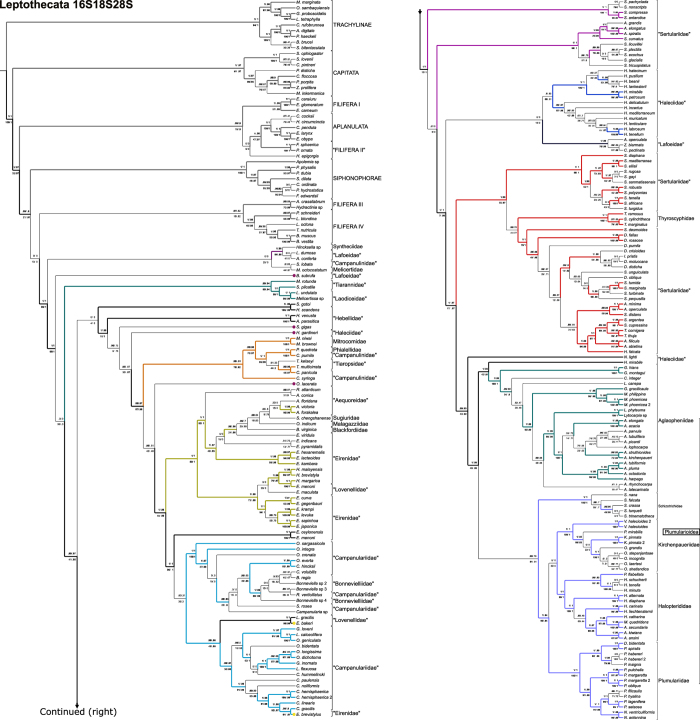
Cladogram of the Leptothecata obtained from the unfiltered (matrix 16S18S28S). Branch colors, support values and notation for unstable taxa as described in Fig. 1. Names within quotation marks (“ ”) are non-monophyletic groups (traditional taxonomy from WoRMS).

**Figure 3:**
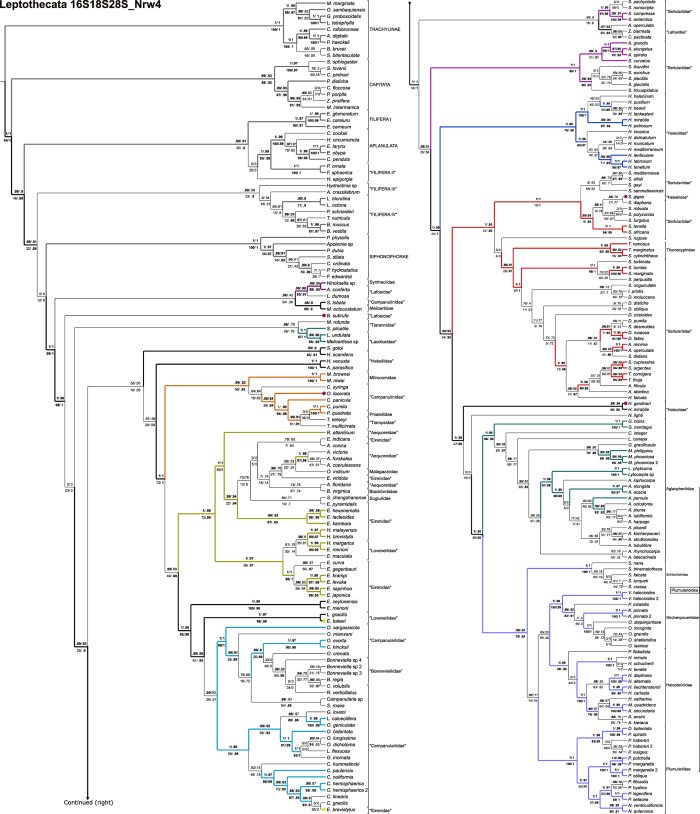
Cladogram of the Leptothecata obtained from the intensively filtered (matrix 16S18S28S_Nrw4). Branch colors, support values and notation for unstable taxa and taxonomy as described in Fig. 1.

The combined datamatrix of ribosomal markers 16S, 18S and 28S, with light data filtering (herein 16S18S28S_N), is our working hypothesis (Fig. 1, Tables 1, 2, 3, 4) because it offers the greatest support values for the majority of basal nodes. Unfiltered (Fig. 2) and heavily filtered (Fig. 3) results are presented for comparison among noise filtering levels. Analyses based on different combination of matrices and noise filtering are presented in Tables 1 and 2 and Supplemental Material (Text and Figs. 3), and a small number of contrasting patterns are dissected in the discussion section.

Basal clades and species whose topological placements were unresolved or whose placements were strongly supported, but conflicting across different topologies, were called “Lineages” (marked with the letter L; Figs 1, 2, 3, Table 2). Rogue taxa (marked in our topologies with letter R), on the other hand, are those terminals whose positions vary widely among the pseudo-replicates generated by nonparametric bootstrap analysis (see Methods section). Such instability leads to an overall decrease in bootstrap support across clades summarized by a consensus tree. Rogue taxa will, by definition, be poorly supported by bootstrap analysis, but they may be significantly supported by the other methods employed in this study (see Methods section). Four rogues (*Hydrodendron gardineri* – R 1, *Opercularella lacerata* – R 2, *Billardia subrufa –* R 3 and *Scandia gigas* – R 4) had substantially different placements across topologies (Figs 1, 2, 3). Hence, they were considered Leptothecata *incertae sedis* and excluded from the new taxonomic proposal.

### Outgroups

Most of the outgroups (Table S1) were monophyletic in the combined analyses (*e.g.*, Aplanulata^47^ Figs 1, 2, 3). A filiferan clade (including groups III and IV) was recovered as the sister group of Leptothecata in analyses of low-filtering and unfiltered matrices (Figs 1 and 2). However, Siphonophorae is sister group of Leptothecata according to the analysis of the highly filtered matrix (Fig. 3). In all cases, support values for those relationships were low.

### Main clades of Leptothecata, some rogue taxa and “traditional lineages”

The most basal taxon of Leptothecata includes species of Lafoeidae (*Lafoea dumosa* and *Acryptolaria conferta*) and *Hincksella* sp. (Syntheciidae), comprising the clade Lafoeida *sensu novum* (with stable basal position and high support; Figs 1, 2, 3; Tables 2 and 3) plus Lineage 1 (L 1), which has low support and includes species of Campanulinidae and Melicertidae (*Stegella lobata* and *Melicertum octocostatum*, respectively). Laodiceida *taxon novum* is another well-supported clade with stable position, including species of Laodiceidae (monophyletic if *Staurodiscus gotoi* is not considered) and Tiarannidae (not monophyletic). Laodiceida is the sister group of main clade comprising Lineage 2, Statocysta, Macroloconia and also, includes the rest of lineages (L 3 to L 5) and rogue taxa (R 1 to R 4) (Figs 1, 2, 3).

### Statocysta and Lineage 2

The working hypothesis (Fig. 1) strongly supports Statocysta and Macrocolonia^45^. Statocysta comprises the majority of species with medusae in the life cycle (Figs 1, 2, 3; Table 2) and is composed by three large clades: Campanulinida *sensu novum*, Eirenida *taxon novum* (divided into two subclades) and Proboscoida *sensu novum* (also comprising two subclades). Species of Lineage 2, encompassing species of Hebellidae (*Hebella scandens*, *Hebella venusta* and *Anthohebella parasitica*; non monophyletic) and Laodiceidae (*S. gotoi*), have stable position and high support as a sister group of the clade formed by *Hydrodendron gardineri* (R 1) + Statocysta (including *Opercularella lacerata* –R 2–). Even though *H. gardineri and Opercularella operculata* were identified as rogue taxa, and classified accordingly in our working hypothesis (R 1 and R 2, respectively; Fig. 1), bootstrap was the only non-significant measure for *H. gardineri* position, out of the 4 branch support methods employed. Nevertheless, the position of this lineage varies widely across topologies. It is recovered as the sister group of Campanulinida *sensu novum* in the topology obtained from the unfiltered data (16S18S28S – Fig. 2) and as sister of its congeneric species in the 16S18S28S_Nrw4 analysis (Fig. 3).

The Campanulinida *sensu novum* (Fig. 1) includes species of Campanulinidae (*Calycella syringa*, *Campanulina panicula* and *Campanulina pumila*), Phialellidae (*Phialella quadrata*), and Mitrocomidae, the only family that is resolved as monophyletic (*Mitrocomella brownei*, *Mitrocomella niwai* and *Tiaropsidium kelseyi*). Campanulinida *sensu novum* is retrieved as sister group of Eirenida *taxon novum*+Proboscoida *sensu novum* in the working hypothesis (Fig. 1).

Two clades within Eirenida *taxon novum* are highly supported in all results. Eirenids I comprises species of Aequoreidae (*Rhacostoma atlanticum* and *Aequorea* spp.), Eirenidae (*Eutonina indicans*, and *Eirene* spp.), *Octophialucium indicum* (the only species assigned to Malagazzidae in our analysis), Blackfordiidae (*Blackfordia virginica*), and Sugiuridae (*Sugiura chengshanense*) (Figs 1, 2, 3). Eirenids II comprises genera from two lineages with high support: one group includes species of Eirenidae (*Helgicirrha brevistyla*, *Helgicirrha malayensis*) and species of Lovenellidae (*Hydranthea margarica, Eucheilota maculata* and *Eucheilota menoni*); the sister lineage comprises species of *Eutima* and *Eugymnanthea japonica*.

The last clade of Statocysta is Proboscoida *sensu novum.* It includes species of Campanulariidae and Bonneviellidae, together with species from the Lineage 3 (*Eirene ceylonensis* and *Eirene menoni*, and the Lovenellidae *Eucheilota bakeri* and *Lovenella gracilis*). Given the high nodal support of the two main clades of Proboscoida *sensu novum*, we propose two new taxa: Campanulariida *sensu novum* (composed by the sampled species of *Orthopyxis*, *Campanularia*, *Bonneviella*, plus *Rhizocaulus verticillatus* and *Silicularia rosea*), and Obeliida *taxon novum*, composed by Obeliidae *sensu novum* (species of *Laomedea* and *Obelia*, both genera recovered as polyphyletic in our results) and Clytiidae *sensu novum* (species of *Clytia* and *Eirene brevistylus*, possibly misidentified, which together with Lineage 3 are considered Statocysta *incertae sedis*).

### Macrocolonia

**Figure 4:**
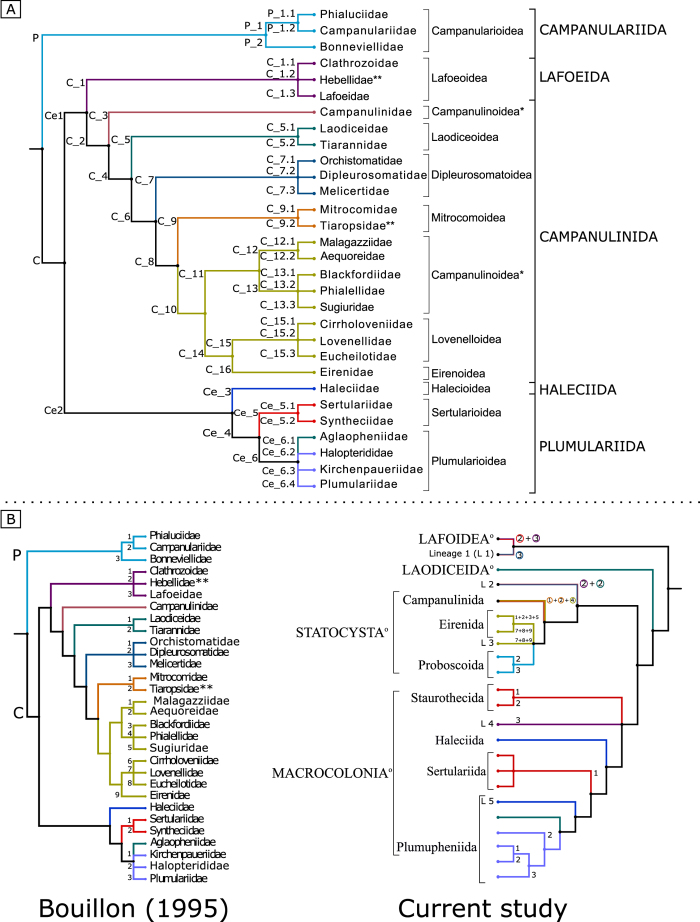
Cladogram (A): Classification of the Leptothecata following Bouillon^28^. Colors indicate representative clades. Codes at the base of the branches from Table 3. Asterisk indicates non-monophyletic, original proposal. Cladogram (**B**): comparison of the clades following Bouillon^28^ (left, defining the families) with the principal clades and lineages we propose herein (right, see Fig. 1); the symbol ^o^ indicates clades already defined in the literature^45^. Colors indicate representative clades and the numbers at the bases of the branches indicate a summary of the relative position according to Bouillon^28^ and this study. Clades defined by more than one family in Bouillon^28^ are indicated by a combination of colors and number of the family in each case. Rogue taxa were not included and double asterisks indicate families absent from Bouillon^28^.

This taxon is divided into four large crown clades (Staurothecida *taxon novum*, Haleciida *sensu novum*, Sertulariida *taxon novum* and Plumupheniida *taxon novum;* Figs. 1 and 4) and several apical taxa (e.g., Aglaopheniida *taxon novum*, Plumulariida *sensu novum*, Staurothecidae *fam. nov.*, Symplectoscyphidae *fam. nov.*), and a clade with unstable position within Macrocolonia, named Lineage 4 (“Zygophylacinae”^43^,^44^). Even though monophyly of L 4 is highly supported, its position as sister group of the Sertulariida has virtually no support (Fig. 1) and neither has the L 4 +Haleciidae clade recovered in the analysis of the unfiltered matrix (Fig. 2). It was placed as the sister group of the Sertulariidae in the tree obtained from the highly-filtered dataset (Fig. 3), but again, support was non-significant regardless of the method employed. Lineage 5 (*Nemalecium lighti* and *Hydrodendron mirabile*) is part of Plumuphenniida and the rogue taxon R 4, *Scandia gigas*, within Sertulariidae (Figs 1, 2, 3; Table 2). *Billardia subrufa* (R 5) was recovered either as sister taxon of Macrocolonia (Fig. 1) or close to Lafoeida *sensu novum*+Lineage 1 (Figs 2 and 3). The placement of this taxon was weakly supported in all analyses.

Macrocolonia subgroups require redefinition; therefore we propose some amendments and new taxa:

- Staurothecidae *fam. nov.* (Type genus: *Staurotheca* Allman, 1888), with high nodal support, includes species of *Staurotheca*, for which only *Staurotheca antarctica* had nuclear markers included in the analyses; we acknowledge that *Staurotheca dichotoma* Allman, 1888, type-species of *Staurotheca*, is missing from our analysis. However, the hypothesis seems reasonable because of the wide taxon sampling of congeners and the taxonomic stability of the genus. More data on the type species should further test the present hypothesis.

- Symplectoscyphidae *fam. nov.* (Type genus: *Symplectoscyphus* Marktanner-Turneretscher, 1890) includes *Antarctoscyphus* Peña Cantero, García Carrascosa & Vervoort, 1997 and *Symplectoscyphus*, two monophyletic genera with high support. An exception is *Symplectoscyphus curvatus*, which is sister group of the *Antarctoscyphus* species (Figs 1, 2, 3; see considerations about taxonomic validation of *S. curvatus* in the discussion section); we acknowledge that *Symplectoscyphus australis* Marktanner-Turneretscher, 1890, type-species of *Symplectoscyphus*, is missing from our analysis; however the hypothesis is reasonable because of the wide taxon sampling of congeners and the taxonomic stability of the genus. Future data on the type species will test accordingly the present hypothesis.

- Haleciida *sensu novum*, including species of *Halecium* Oken, 1815, is highly supported and occupies a stable position as sister group of the clade (Sertulariida *taxon novum* (Aglaopheniida *taxon novum*, Plumulariida *sensu novum*)) in the multilocus analyses.

- Sertulariida *taxon novum*, composed by Sertularellidae *fam. nov.* (Type genus: *Sertularella* Gray, 1848; includes *S. polyzonias* (Linnaeus, 1758), type species of *Sertularella*, besides its congeners and *Symplectoscyphus turgidus*), Thyroscyphidae (including species of *Thyroscyphus* Allman, 1877 and *Sertularelloides cylindritheca*), and Sertulariidae (including *Idiellana pristis*, *Hydrallmania falcata*, *Salacia desmoides* and species of the monophyletic genera *Abietinaria* Kirchenpauer, 1884*, Amphisbetia* L. Agassiz, 1862*, Diphasia* L. Agassiz, 1862 and *Thuiaria* Fleming, 1828, as well as the species of the non-monophyletic genera *Dynamena* Lamouroux, 1812 and *Sertularia* Linnaeus, 1758).

Although *N. lighti* and *H. mirabile* are traditionally placed into Haleciidae Hincks, 1868, they are a basal lineage (Lineage 5) of the well-supported clade Plumupheniida *taxon novum* (Figs 1 and 3), which includes two main taxa:

- Aglaopheniida *taxon novum*, a well-supported node in all combined analyses (Figs 1, 2, 3; Table 2);

- Plumulariida *sensu novum*, composed by Schizotrichidae Peña Cantero, Sentandreu & Latorre, 2010 (for which the phylogenetic position is kept in multilocus analysis), Kirchenpaueriidae Stechow, 1921, Halopterididae Millard, 1962 (in which none of the traditional genera, as *Antennella* Allman, 1877 and *Halopteris* Allman, 1877, are monophyletic in the combined analyses), and Plumulariidae McCrady, 1859 (in which only *Nemertesia* Lamouroux, 1812 is monophyletic).

## Discussion

In this section we contrast the new taxonomic classification proposed herein with the traditional one (Figs 1, 2, 3, 4; Tables 2, 3, 4 and Supplementary Table S1; number of species for each group based on WoRMS database^2^), and discuss some macroevolutionary patterns that may be associated with the diversification of Leptothecata.

### Molecular markers and evaluation of phylogenetic results

The general outline of our results is consistent with recent hypotheses^41^,^45^,^46^, but some particular aspects should be emphasized. The mitochondrial marker 16S accumulates greater nucleotide variation than the nuclear markers 18S and 28S, which may be seen by the differences in conserved sites, singletons and informative sites for parsimony, in relation to the total sequence size (Table 1). The 16S sequences are shorter and display a relatively greater proportion of filtered sites than 18S and 28S (Table 1). Thus, average support in 16S trees is expected to be lower for non-parametric methods, especially in the case of bootstrap.

Rogue taxa and “Lineages” found in the analysis of the 16S matrix have lower nodal support and greater tendency to change their phylogenetic position according to the level of filtering applied to the concatenated matrices. This is especially true for terminals with abundant missing data, such as *H. gardineri* and *S. gigas* (Figs 1, 2, 3; Supplementary Information, “Rogue taxa analysis” section). Taxonomic artifacts associated with 16S include long-branch attraction (LBA, also see Peña Cantero *et al.*^46^), such as between Siphonophorae and Plumularioidea, the group with the greatest variation in ribosomal data in Leptothecata (see Supplementary Figs S3-5 online). Previous studies attempted to get around this issue by not including Siphonophorae species as part of outgroup sampling^41^. Assuming a somewhat constant evolutionary rate, it is expected that basal lineages are more variable with greater saturation. While the use of 16S recovered most of the lineages of Plumularioidea, the LBA artifact results in the accumulation of nucleotide variation, causing non-historical convergence of Siphonophorae and Plumularioidea, which are groups with substantial differences in their life cycles, such as the loss of the medusa stage, strong polymorphism and colony specialization. Interestingly, these taxa bear superficial resemblance, such as the lack of the medusa stage, strong polymorphism and high zooid specialization.

Most non-Plumularioidea lineages are not monophyletic in the 16S analysis, in contrast with patterns from the nuclear markers 18S and 28S, both of which result in similar phylogenies, with 18S having lower resolution (Figs S6-8). The 28S provides a robust phylogenetic framework for Leptothecata due to the number of variable nucleotides, relative gene frequencies in the different matrices (with and without filtering) and support values in the individual phylogenies. Basal nodes in the 28S phylogeny and other phylogenetic groups (*e.g.*, Sertulariida *taxon novum*) are recovered in the concatenated data matrices, with higher support when compared with nodal support of single-gene phylogenies.

Analyses of data matrices assembled under a range of criteria (combined or individual markers and filtering taxa and/or nucleotides at different levels) and employing different branch support methods, allow a better evaluation of the nodal stability by assessing congruence among results (*e.g.*, Table 2; Supplementary Information “Rogue taxa analysis” online). Topologies change with the level of filtering, usually with a concomitant decrease in nodal support. This indicates that the highest filtering level “throws the baby out with the bath water”, i.e. eliminates information together with spurious phylogenetic signal. Intermediate levels of filtering seemingly strike a more reasonable signal-to-noise ratio. Additionally, simulations strongly indicate that non-parametric bootstrap is the branch support method most prone to type II error (i.e. failing to support a “true” clade)^48^. Therefore, by employing alternative support methods, our working hypothesis has recovered a greater number of significantly supported clades than what would be obtained by employing traditional bootstrapping (see Methods for details). All the taxonomic propositions in the following sections were thus based on this hypothesis (Fig. 1).

### Outgroups and sister groups of Leptothecata

The sister group of Leptothecata remains unclear^35^,^45^, with different taxa being included or excluded in previous analyses of Leptothecata (*e.g.*, exclusion of Siphonophorae^41^). Our topologies resulting from combined matrices, did not recover a well-supported sister group for Leptothecata. The only high nodal support for a sister group of Leptothecata was found in the 28S marker analyses (28S_N and 28_Nrw4), in which Bougainvilliidae (Filifera IV after Cartwright *et al.*^35^) is well-supported as its sister group (basal relationships similar to 16S18S28S). However, more data are definitely necessary to confirm the placement of this family.

### Previous conflicts in leptothecate phylogeny and a new working hypothesis

Molecular studies based on ever-increasing number of species have contradicted taxa considered to be monophyletic under a traditional sense, such as Conica, Proboscoida and Plumulariida^45^,^49^. However, our results also corroborate some traditional taxa defined by their exoskeleton and specialized characters of polyps, as in the reformulated Plumularioidea (Fig. 4, Table 2). Ours is the most inclusive analysis of Leptothecata to date, which recovered well-supported clades based on multilocus data matrices and yielded a robust, working hypothesis (Figs 1 and 4; Tables 2, 3, 4). However, our proposal for new names is conservative and focuses only on the more stable and robust nodes, often corroborated by morphological synapomorphies, aiming to minimize changes in the current classifications (Figs 1 and 4). Instead of filtering “problematic terminals” from our main results, we considered that they should be included in the main results because these specimens/molecular markers are available in GenBank as any other sequence. We acknowledge that this strategy resulted in somewhat controversial or not well supported positions, and therefore some of them are considered. Leptothecata *incertae sedis* (Lineages 1 and 2, rogue taxa), specific clades’ *incertae sedis* (Lineages 3-5), and only a few as dubious terminals (e.g, potential taxonomic misidentifications).

### Lafoeida *sensu novum*

Lafoeida *sensu novum* comprises only the three species sampled here by now and is presently defined by having stolonal or erect colonies, polyps without abcaulinar caecum, hydrothecae without operculum and diaphragm (considering other potential genera) and fixed gonophores, usually forming coppinia (aggregation of gonothecae that can include nematothecae^4^).

Previous analyses had placed Hebellinae, Zygophylacinae and Lafoeinae into the Lafoeidae (cf. Marques *et al.*^44^) (Hebellidae and Zygophylacidae, respectively; Tables 3, S1, Figs 1 and 4), or related Zygophylacinae with Sertulariidae (cf. Marques *et al.*^44^). In our hypothesis, assumed to have a limited number of lafoeid species (Fig. 1, Table S1), Lafoeidae is the only nominotypic group of Lafoeida, not related to the “Hebellidae” and Zygophylacidae (see below), which is in agreement with previous studies^35^,^43^,^44^,^45^. Future addition of taxa will test that more properly.

Other molecular analyses indicate that Lafoeidae includes species of *Filellum*, *Lafoea, Cryptolaria* and *Acryptolaria* (Moura *et al.*^43^,^50^, present study), but its taxonomic range could be wider, because *Grammaria* (6 species) and *Cryptolarella* (1 species) were not included in our analyses. Altogether, the relationship of both genera with other Lafoeidae has been supported by morphology^44^,^51^. Another pending question about phylogenetic affinities for this family is its relationship with Clathrozoidae^25^,^26^, a family with two monospecific genera that has not been sampled in any published molecular phylogenetic analysis.

*Hincksella* sp. (traditionally considered as part of Syntheciidae) is the terminal that completes this clade. Morphological similarities between Syntheciidae and Lafoeidae species have already been identified^52^. Morphologically, the trophosome of *Hincksella* is similar to Lafoeida (*e.g., Acryptolaria* and *Lafoea*) because of the tubular hydrotheca. However, the proximal region of the hydrotheca of *Hincksella* has a distinct floor perforated by a hydropore^53^; additionally, species of Lafoeida, such as those of *Acryptolaria* and *Lafoea*, have coppinia (absent in *Hincksella;* but see Millard^23^ and Calder^53^ for opposing arguments on the affinity between *Hincksella* and Lafoeidae). Nonetheless, the hypothesis can only be tested with the inclusion of *Parathuiaria* (1 species) and *Synthecium* (23 species) in a broader analysis including multilocus data for *Hincksella*.

Additional references: Billard^54^; Rees & Vervoort^55^; Vervoort^56^; Migotto & Marques^57^; Schuchert^58^; Marques *et al.*^59^,^60^; Bouillon *et al.*^30^; Peña Cantero *et al.*^51^.

### *Stegella lobata and Melicertum octocostatum* (Lineage 1 – L 1)

*Stegella lobata* belongs to the monotypic genus *Stegella* Stechow, 1919, which was previously associated with Campanulariidae^61^,^62^, and later included in Campanulinidae (Stechow 1919; originally as *Stegella grandis*^63^). It is characterized by pedicellate hydrothecae, rhizocaulomic colonies (hydrorhiza erect in parallel stolons), operculum with four flaps and fixed gonophores^64^,^65^.

*Melicertum octocostatum* belongs to the only genus of Melicertidae whose polyp stage is known. This family is divided in 4 genera and 8 species, and is characterized by stolonal colonies with unusual morphology when contrasted with the typical “thecate” polyp: absence of hydrotheca around the hydranth and probably with no gonotheca surrounding gonophores^30^. Medusae of this taxon have no ocelli or other marginal sensory structure^4^, but the gonads are on the radial canals, which is a typical character of the medusa of Leptothecata. In our phylogeny *M. octocostatum* is the basalmost leptothecate species with medusae with gonads on the radial canals.

The clade defined by *S. lobata* and *M. octocostatum* (Melicertidae) has a basal position in Leptothecata (Figs 1 and 2), similar to Leclère *et al.*^45^ but different from Peña Cantero *et al.*^46^. This group varies in both its affinities and nodal support, depending on the analysis (Leptothecata *incertae sedis*; Figs 1, 2, 3). Hopefully, sampling a higher number of species and additional markers will elucidate the relative positions of *S. lobata* and *M. octocostatum.* The same goes for other unsampled families of the superfamily Dipleurosomatoidea (Orchistomatidae - 6 species, Dipleurosomatidae - 8 species, Table S1).

Additional references: Bouillon^25^,^26^; Govindarajan *et al.*^40^.

### Laodiceida *taxon novum*

Laodiceida *taxon novum* includes two families with medusae in their life cycles (Laodiceidae and Tiarannidae), although the medusa stage is still unknown in several species. However, in species where it is known, the medusa is characterized by the presence of gonads along the radial canals in Laodiceidae, and on the interradial walls of the stomach (or in adradial pouches) in Tiarannidae. Both groups have the pelagic stage with no statocysts but with cordyli^4^. The benthic stage has stolonal and erect (*Stegopoma* spp.) colonies, hydrothecae with operculum and diaphragm slightly developed or absent.

Monophyly of Laodiceida *taxon novum* and its phylogenetic position within Leptothecata are well supported (Table 2, Figs 1, 2, 3, cf. Leclère *et al.*^45^). According to our working hypothesis, Tiarannidae is not monophyletic and Laodiceidae becomes monophyletic only if *Staurodiscus gotoi* is not considered as part of the family (Fig. 1). Indeed, medusae of *S. gotoi* are morphologically similar to those of *Hebella* (Migotto & Andrade^66^; cf. Leclère *et al.*^45^; Moura *et al.*^43^), suggesting homoplasy of the marginal cordyli in *Staurodiscus* and species that truly belong to Laodiceidae.

Discussions on the validity of the families of Laodiceida *taxon novum* have been focused on the position of the gonads in the medusae^4^,^25^,^26^,^66^, and some uncertainties still remain because several genera are poorly sampled, their life cycles are not fully known, and the polyp identification is complex, since their morphology is generally characterized as “cuspidella” type^30^. Also, *Staurodiscus* (type species *Staurodiscus tetrastaurus* Haeckel, 1879), whose monophyly remains uncertain, further confounds these issues.

Additional references: Pagès *et al.*^67^; Vito *et al.*^68^.

### “Hebellidae”, *Staurodiscus gotoi* (Lineage 2 – L 2)

This is a non-monophyletic lineage including three species of “Hebellidae” (*Anthohebella parasitica*, *Hebella scandens*, *Hebella venusta*) and *S. gotoi* (see considerations on Laodiceida *taxon novum*), with high support for the relative position of these species (Figs 1, 2, 3). Similar results were recovered elsewhere with a smaller set of “Hebellidae” species^45^. Also, the proximity between *S. gotoi* and “Hebellidae” reinforced previous considerations about misinterpretations of life cycles in these groups. The phylogenetic proximity between *Scandia gigas* and Haleciidae (Fig. 2), contrasts with its placement close to *Hydrodendron gardineri* and *H. mirabile* in Fig. 3 (genus traditionally considered as Haleciidae; Figs 1, 2, 3, Table 3), and also with its placement in our working hypothesis, firmly set in Sertularellidae (rogue taxon; Fig. 1). As already remarked, such instability might be attributed to the 16S characters, thus we prefer to regard Lineage 2 as Leptothecata *incertae sedis*.

The phylogenetic basal position of L 2 and their life cycles (*Hebella* spp. and *S. gotoi* have medusae, *Anthohebella* has “swimming gonophores”) corroborate the medusa as an ancestral condition in Leptothecata life history (Fig. 1). According to this hypothesis, the absence of medusae in the life cycle of Leptothecata would be a derived condition. “Hebellidae” and *S. gotoi* have stolonal colonies with campanulate hydrothecae provided with a diaphragm or annular thickening, and morphologically simple gonothecae^44^. While morphologically similar, other genera of “Hebellidae”, such as *Bedotella* (1 species), with fixed gonophores, and *Halisiphonia* (5 species), which apparently has the medusa stage, need to be included in molecular and life cycle reconstruction analyses^33^,^69^.

Additional references: Calder^53^; Cornelius^4^; Boero *et al.*^70^,^71^; Migotto *et al.*^72^.

### Statocysta

Statocysta are characterized by having statocysts, a typical equilibrium structure of the medusa stage^45^, indicative of a more active swimming. Two main lineages are well supported (Fig. 1): Campanulinida *sensu novum* and Eirenida *taxon novum* + Proboscoida *sensu novum*. *Opercularella lacerata* has a dubious position in our phylogenetic hypothesis (rogue taxa; Figs 1, 2, 3, Table 3). The traditional division of Leptothecata into Conica and Proboscoida is not supported because Statocysta combines parts of each one of these “taxa”. Proboscoida, in its traditional sense, might be re-interpreted as a derived clade within Conica (Figs 1, 2, 3, 4, Tables 3, S2). Future studies may infer alternative sister groups for Statocysta, especially considering those lineages included in Lineage 2 (“Hebellidae” and *S. gotoi*) and *O. lacerata* (rogue taxa).

### Campanulinida *sensu novum*

This taxon includes species of several families and superfamilies of the traditional taxonomy (e.g., Mitrocomidae and Tiaropsidae), but its composition does not support the monophyly of Campanulinida in a traditional sense (cf. Bouillon^25^,^26^). Due to the difficulty of defining diagnostic characters for families and genera traditionally placed in Campanulinida^4^, the lack of information on life cycle and the difficulty in treating species with unrecognized medusae (e.g., *Calycella* and *Campanulina*; Bouillon *et al.*^30^), the monophyly for these genera and families have often been questioned. A clear example of the need to understand more about life cycle strategies in Hydrozoa is found in *Lafoeina* (Campanulinidae) and *Cirrholovenia*. The benthic stage of *Lafoeina* species are mistakenly mixed with species of other taxa (e.g. *Cuspidella* species), in some cases with unknown medusa stage, and in others with young medusae strikingly similar to *Cirrholovenia* (genus of Lovenellidae, not represented in molecular studies^72^). Nomenclaturally, Campanulinidae is nominotypic, but rather a complex of species, whose problematic taxonomic status and identity have already been debated by other authors^53^. To solve the “campanulinid” taxonomy will be a major task in itself, beyond the scope of this manuscript. Therefore, we are aware that our hypothesis of Campanulinida *sensu novum* must be more broadly tested, including sequences of the type species of the genus *Campanulina* van Beneden, 1847 (Fig. 1).

None of the traditional lineages in Campanulinida is supported by our data, except the monophyly of Mitrocomidae (Figs 1, 2, 3, Tables 3, 4; Bouillon^26^). This is not surprising because there are no synapomorphies proposed for supraspecific taxa in the traditional Campanulinida^1^. In synthesis, morphology of the sampled species varies in the clade from the “cuspidella” type (stolonal colony, polyp with operculum but lacking diaphragm) to “generic campanulinida” (stolonal or erect colony, polyp with operculum and diaphragm). In those species with known medusa, this stage has four simple radial canals, many statocysts, hollow tentacles and no cirri (except *Mitrocomella*)^4^,^26^.

### Eirenida *taxon novum*

This clade comprises some of the remaining families traditionally assigned to Campanulinida (e.g., Aequoreidae, Eirenidae, Lovenellidae^28^). Two groups are revealed in our working hypothesis: Eirenids I and Eirenids II.

#### - Eirenids I

If corroborated, this clade will eventually become the nominotypical taxon because it includes species of the family Eirenidae Haeckel, 1879^25^,^26^ (non-monophyletic) and the genus *Eirene* Eschscholtz, 1829 (non-monophyletic), besides the type species of the genus, *Eirene viridula* (Péron & Lesueur, 1809). It also includes several genera of Campanulinoidea and *Eutonina* (one of the two valid species in the taxon).

In an attempt to overcome the difficulty in defining diagnostic characters in Hydrozoa, Bouillon^25^,^26^ proposed the non-monophyletic clade “Campanulinoidea”, characterized by “medusa without exumbrellar cirri” (with nomenclatural problems, N.B. the phylogenetic hypothesis in Bouillon^25^: p. 12). In this hypothesis, a non-monophyletic Campanulinoidea included the problematic “Campanulinidae” (Fig. 4. and Table 3). Also, *Eirene* is complicated to diagnose, requiring the presence of the medusa stage for an adequate taxonomic identification; additionally, the medusa stage may also be found without cirri. Other groups external to Eirenids I have medusae lacking cirri (*e.g.*, Phialellidae), and therefore, the state of character “medusa without cirri” is homoplastic.

Polyps of Eirenids I are “campanulinida” type, with stolonal colonies (except *Aequorea* and *Eirene*), operculum formed by flaps without clear demarcation from the hydrotheca, hydranths with intertentacular membrane and hydrotheca with diaphragm. Medusa has gonads on the radial canals, distant from the manubrium, ocelli and cirri are absent, and there are many closed statocysts (Cornelius^4^, Table 3). Internal relationships usually have weak support (Figs 1, 2, 3).

Additional references: Bouillon & Boero^29^; Govindarajan *et al.*^73^; Kubota^74^; Bardi & Marques^75^; Leclère *et al.*^45^.

#### - Eirenids II

This clade comprises two subclades with high support (Figs 1, 2, 3): “Eirenidae” (polyphyletic traditional family, see above for considerations about Eirenids I) and two species of Lovenellidae (*Eucheilota menoni* and *Eucheilota maculata*; another species of *Eucheilota* appears in Statocysta Lineage 3 along with *Lovenella gracilis*).

One of the two main clades in Eirenids II includes the monophyletic genus *Helgicirrha* (with 2 sampled species), *Hydranthea* (considered as Lovenellidae by Bouillon *et al.*^30^) and *Eucheilota* (Lovenellidae). Its sister group includes species of *Eutima* and *Eugymnanthea japonica*, nested amongst them^76^. Eirenids II is interesting due to both morphology and lifestyle of the species: those of *Eugymnanthea* and *Hydranthea* produce eumedusoids and inhabit the mantle cavity of bivalves.

Eirenids II have “campanulinida” colonies, with reduced hydrotheca and hydranths protected by a slender perisarc and intertentacular membrane; medusa with gonads on the radial canals, lateral cirri and closed statocysts^30^.

### Proboscoida *sensu novum* and Lineage 3 (L 3)

Originally, Proboscoida included Bonneviellidae and Campanulariidae^14^, and later also Phialuciidae, being characterized mainly by the polyp with trumpet-shaped hypostome^26^. Except for Phialuciidae (monotypic family not included in this analysis), our topology provides support for phylogenetic proximity of the remaining Proboscoida, with the inclusion of Statocysta *incertae sedis* Lineage 3 (defined by *Eucheilota bakeri*+*Lovenella gracilis* and *Eirene ceylonensis*+*Eirene menoni*) as part of dubious results. In the Lovenellidae clade, our analysis groups *E. bakeri* with *L. gracilis* (Fig. 1). The origin of the GenBank sequences of *E. bakeri* is unknown to us and we suspect the material could have been incorrectly identified (part of conflicts are related to sequences’ metadata^77^). We and other specialists (P. Schuchert, pers. comm.) agree on this possibility, and it would be necessary to examine the voucher to assure its identification. Historically, *Lovenella* has been associated to Campanulariidae (Hincks^8^, based on polyp characters), Lovenellidae (Russell^78^, based on medusa characters) and *Campalecium* (family Haleciidae, based on the “haleciid type” morphology of the polyp^30^). Hypotheses for Lovenellidae also included *Eucheilota* (Russell 1953), *Cirrholovenia* (Kramp 1959) and *Hydranthea* (see historical record in Miranda *et al.*^79^). Together with Eirenidae species, efforts on sampling additional molecular data for species of Lovenellidae are necessary to confirm its monophyly, or even to consider a possible resurrection of Eucheilotidae Bouillon, 1984. In our analysis, Proboscoida did not diverge firstly in Leptothecata as sister group of Conica, in contrast to the original proposal of the group (cf. Bouillon^28^).

Proboscoida *sensu novum* includes two highly-supported main clades, Campanulariida *sensu novum* and Obeliida *taxon novum*, the latter with high nodal support for apical groups as well (Figs 1, 2, 3, 4, Tables 3 and 4).

#### - Campanulariida *sensu novum*

This taxon includes species of the traditional families Campanulariidae and Bonneviellidae. It is characterized by stolonal and erect colonies, occasionally polysiphonic (as in *Rhizocaulus*), with medusae or eumedusoids present (e.g., *Orthopyxis*; Cornelius^24^). No genus is recovered as monophyletic in our main results. Therefore, unresolved systematic questions, already discussed in literature^80^, still remain, such as the monophyly of *Campanularia* and *Orthopyxis*, which is related with taxonomic interpretations based on the presence/absence of diaphragm and the shape of hydrotheca (e.g., Millard^23^).

Additional references: Berrill^81^; Cunha *et al.*^80^; Govindarajan *et al.*^40^.

#### - Obeliida *taxon novum*

Obeliida *taxon novum* embraces the remaining families of Proboscoida, namely Clytiidae and Obeliidae (Table 4). Obeliidae is monophyletic with high nodal support (Fig. 3, topology 16S18S28S_Nrw4). Clytiidae *sensu novum* is monophyletic as well, despite the presence of “*Eirene brevistylus*” (probably due to a long-branch attraction artifact on 16S sequences^82^ or to taxonomic misidentification) – presently this specimen is considered as *incertae sedis*. This clade is characterized by the presence of hydrothecae with diaphragm, and a wide variety of reproductive strategies: presence of uniform medusae (*Obelia*), meconid medusae (*Gonothyraea*) and fixed gonophores (*Laomedea*)^5^,^24^,^83^.

Additional references: Cornelius^83^; Govindarajan *et al.*^84^; Lindner *et al.*^85^.

### *Billardia subrufa* (Rogue taxa 4)

The taxonomy of *Billardia* Totton, 1930 remains uncertain, and the genus has been morphologically associated either with Campanulariidae^86^, Lafoeidae and Syntheciidae^23^,^24^. *Billardia* colonies are erect, with no diaphragm or with an “annular thickening” at the base of the hydrotheca; gonophores are fixed and protected by a long, ringed gonotheca^52^,^87^. We obtained new sequences of the antarctic *Billardia subrufa*, similar to those of GenBank. Previous molecular analysis placed *B. subrufa* near to Lafoeidae, at the base of Leptothecata^40^,^45^. In our results, however, *B. subrufa* is at the base of Macrocolonia or proximal to Lafoeidae+Lineage 1 (Figs 1, 2, 3). The unstable position and low nodal support led us to consider *B. subrufa* as a leptothecate *incertae sedis*.

Additional references: Cornelius^5^.

### Macrocolonia

Macrocolonia was proposed together with Statocysta, including groups with complex and erect colonies, and encompassing species of Plumularioidea, Sertulariidae and Haleciidae^45^. It mostly coincides with the traditional proposal of Plumulariida and Haleciida (original Haleciida included the family Syntheciidae; Bouillon^25^,^28^; Fig. 4, Table 3), being characterized by “*Hydranthes à hypostome conique, et dont le feuillet endodermique est différencié em deux régions distinctes, hydranthes rétractables dans leurs hydrotèques, Celles-ci ont une symétrie bilatérale, sont dépourvues de diaphragme mais leur plancher est perforé par un hydropore*” (Bouillon^25^, p. 20)^88^. Macrocolonia has the greatest species richness within Medusozoa, with the medusa stage reduced and with fixed gonophores in most part of the species. Our hypothesis emphasizes the proposal of some new taxa, as well as readjustments in already defined taxa. We do not find it necessary to reinterpret Macrocolonia. Rather, we point out the relative positions of both Macrocolonia *incertae sedis*: Lineage 4 (Zygophylacinae) and Lineage 5 (*Nemalecium lighti* and *H. mirabile*), together with interesting evolutionary aspects concerning *Staurotheca* and *Schizotricha*.

### Staurothecida *taxon novum –* Staurothecidae *fam. nov.* and Symplectoscyphidae *fam. nov*

At the base of Macrocolonia, Plumulariida, Sertularioidea and Sertulariidae are not monophyletic, which contradicts the traditional taxonomic view of these taxa within Leptothecata^5^. In our present understanding, a basal branch of Macrocolonia includes a weakly supported clade that we have named as Staurothecida *taxon novum* to preserve the taxonomic logic of Leptothecata. Staurothecida *taxon novum* includes two new well-supported families that support previously described patterns^46^: Staurothecidae *fam. nov.* and Symplectoscyphidae *fam. nov.*

Staurothecidae *fam. nov.* includes the type-genus *Staurotheca* Allman, 1888 and its species, which were previously attributed to Syntheciidae^23^,^25^,^26^,^28^,^89^,^90^ and Sertulariidae^91^. Morphologically, Staurothecidae and *Staurotheca* are characterized by tubular hydrothecae, with operculum and diaphragm arranged either in two longitudinal rows of opposite, sub-opposite or alternate hydrothecae or in decussate series. Male and female gonothecae are directly inserted at the hydrothecal base, with female gonothecae sometimes arranged on special supporting structures (Peña Cantero & Vervoort^92^, p. 2664).

Symplectoscyphidae *fam. nov.* includes the type-genus *Symplectoscyphus* Marktanner-Turneretscher, 1890 and the genus *Antarctoscyphus*, both of them historically related (cf. Peña Cantero *et al.*^91^). Like *Staurotheca*, *Symplectoscyphus* is also a monophyletic taxonomically stable taxon (e.g., Peña Cantero *et al.*^46^). We corroborated this taxon and we characterize *Symplectoscyphidae fam. nov.* by the hydrothecal aperture provided with three cusps and by an operculum of three valves. Nonetheless, the genera vary in colony morphology, branching pattern and gonotheca morphology (Peña Cantero *et al.*^91^, p. 24). *Symplectoscyphus curvatus* has to be taxonomically revalidated, since it is sister group of *Antarctoscyphus* species (Figs 1, 2, 3). Also, the identity of *Symplectoscyphus turgidus* specimen used here was confirmed by P. Schuchert (pers. comm.) and therefore, its placement within *Sertularella* has to be re-evaluated.

Additional references: Peña Cantero *et al.*^46^,^90^,^91^; Peña Cantero & Vervoort^92^,^93^; Peña Cantero^94^.

### Haleciida, Halecioidea, Haleciidae *sensu novum*

Haleciidae *sensu novum* (and the redundant taxa Halecioidea and Haleciida) encompasses only species of *Halecium* Oken, 1815. It is a homogeneous and monophyletic taxon, sister group of Sertulariida *taxon novum* and Plumupheniida *taxon novum*. The complex taxonomic history of the taxa was reviewed by Calder^53^, Schuchert^95^ and Peña Cantero^96^. Some genera previously attributed to Haleciidae were placed in Plumulariidae and Lafoeidae due to the presence of nematothecae^97^, which are absent in species of *Halecium*^23^. In our topology, the genera *Hydrodendron* and *Nemalecium* have an alternative position, related to Plumularioidea (Lineage 5; Figs 1, 2, 3).

*Halecium* colonies are erect and ramified, often with free hydrophores. The hydrotheca is shallow, with conspicuous proximal desmocytes, and with hydranth not retractable into it. The gonophores are fixed and protected in solitary gonothecae or aggregated in glomulus, usually dimorphic (cf. Calder^53^).

Additional references: Cornelius^5^; Schuchert^95^; Peña Cantero^96^.

### Zygophylacinae (Lineage 4)

Zygophylacidae was considered several times to be a subfamily of Lafoeidae (Zygophylacinae) due to the presence of bilateral and tubular hydrotheca^44^,^53^. Here, the sampled species of Zygophylacinae occupy alternative positions in Macrocolonia as sister group of Sertulariida+Plumupheniida (Fig. 1), Haleciida (Fig. 2) and *Staurotheca* (Fig. 3). All these relative positions with low support are likely to be related to the exclusive use of 16S marker in our analyses for these species. Zygophylacinae has already been considered as Sertulariidae, related to *Abietinaria*, due to the form of the colonies, the presence of operculum (present in *Abietinella*) and the hydrothecal shape^98^. Despite being within Macrocolonia, the species of Zygophylacinae here analysed have characters not found in other members of Sertulariida, Staurotheciida or Haleciida *sensu novum*, such as the presence of nematothecae and gonothecae aggregated into coppinia or scapus^44^. Because of their *incertae sedis* position among Macrocolonia, and features as defensive polyps (as present in Plumupheniida), a multilocus analysis would better define their position. Their basal “condition” called our attention to a speculative scenario, where Zygophylacinae resulted in a family group (as already discussed by Moura^43^), as a basal lineage of Macrocolonia, or directly related to Plumupheniida.

### Sertulariida *taxon novum*

Traditionally, sertulariids are well-supported taxa defined by their colony shape and hydrothecal morphology, although these characters are plastic, and integrative proposals have been questioned^99^. Sertulariidae was firstly split into three subfamilies (Sertulariinae, Thyroscyphinae and Sertomminae), mainly based on whether hydrothecae were sessile or pedicellate, and on the presence-absence of abcaulinar cecum^53^,^63^. The current scope of Sertulariida *taxon novum*, however, includes several sertulariid species that are herein classified among families with new interpretations, such as Thyroscyphidae and Sertulariidae *sensu novum*, besides the new taxon Sertularellidae *fam. nov*. Other groups, on the other hand, were removed from Sertulariidae (see Staurothecidae and Symplectoscyphidae above). Overall, Sertulariida *taxon novum* is characterized by stolonal or erect monopodial colonies, with radial to bilateral symmetry on hydrothecae, hydrothecal margin with 3 to 4 cusps, operculum of 2 to 4 valves, and fixed gonophores (except for *Amphisbetia operculata* (Linnaeus, 1758) and *Sertularia marginata* (Kirchenpauer, 1864), which are medusoid-producing species). Below, we present our new interpretations and descriptions within Sertulariida *taxon novum*:

#### - Sertularellidae *fam. nov*

This is a well-supported clade, including the highly speciose genus *Sertularella* Gray, 1848 with 131 nominal species distributed worldwide^3^. Colonies are erect, mono- or polysiphonic, branched or unbranched, and hydrothecae have four marginal cusps and a pyramidal operculum with four triangular valves^53^. Our results call attention to a possible reinterpretation of *S. turgidus* as part of Sertullarellidae, and supports *Sertularelloides cylindritheca* as a Thyroscyphidae species, as already discussed^100^. Further samples for these species will be of great value, mainly because until now there is only one specimen sampled for *S. turgidus*^45^(Fig. 1; see below).

Additional references: Vervoort^98^,^101^; Vervoort & Watson^102^.

#### - Thyroscyphidae

Morphologically, Thyroscyphidae includes species with pedicellate hydrotheca, with annular diaphragm and hydranths with a double basal annulus instead of abcaulinar diverticulum. Traditionally, the family includes *Thyroscyphus*, *Parascyphus, Sertularelloides*, *Thyroscyphoides*, *Uniscyphus* and *Symmetroscyphus*^2^,^53^. Historically, Thyroscyphidae is monophyletic, either considered as a separate clade from Sertulariidae (*e.g.*, Calder^53^; Shimabukuro & Marques^88^), or as part of Sertulariidae (cf. Millard^23^; Bouillon^26^). We keep Thyroscyphidae at the family rank because of its high support and coincidence with other similar hypotheses^45^,^100^, although small changes in the position of *Thyroscyphus* might occur. We include *S. cylindritheca* (previously *Sertularella cylindritheca*) in Thyroscyphidae^46^,^100^, but this species will need further analysis, including *Parascyphus*, because of its uncertain affinities (cf. Calder^53^).

#### - Sertulariidae *sensu novum*

A redefinition of the taxa within Sertulariidae *sensu stricto* is required, due to our proposal involving new families (i.e., Sertularellidae, Staurothecidae and Symplectoscyphidae). In its current scope, many genera still need to be sampled and validated, and even the richest genera (*e.g., Sertularia* and *Dynamena*) are not considered monophyletic in our hypothesis, as already presented and discussed^45^,^100^; morphological features, such as the abcauline diverticulum (cf. Calder^53^ p. 86–87), still have to be tested as synapomorphies. Sertulariidae *sensu novum* includes species with hydrothecae with non-pyramidal operculum, with one (e.g., *Abietinaria*, *Diphasia*, *Idiellana*, *Salacia*, *Thuiaria*) or two valves (the adcaulinar one sometimes divided into two, *e.g., Amphisbetia*, *Dynamena*, *Hydrallmania*, *Sertularia*). These characters are homoplastic in our working hypothesis; other characters also proved to be equally uninformative, such as fasciculation, hydrothecal arrangement and shape of hydrothecal aperture. It is noteworthy to highlight that the non-monophyly of *Sertularia* raises back the question about the validity of *Tridendata* Stechow, 1920. Indeed, *Sertularia argentea* (type species of *Sertularia*), and *Sertularia perpusilla* (type species of *Tridentata*) falls apart in our hypotheses (Figs 1, 2, 3), and several of the species usually assigned to the genus *Tridentata* (e.g., *Tridentata marginata*, *Tridentata turbinata*, *Tridentata tumida*) are close to the type species *T. perpusilla* (D.R. Calder, pers. comm.). The addition of new sertulariid taxa and sequences shall provide a better test for this hypothesis, but presently it surely cannot be discarded. Sertulariidae *sensu novum* has decreased its species richness, since traditional genera such as *Sertularella*, *Antarctoscyphus* and *Symplectoscyphus* (representing ~41% of the number of species in Sertulariidae *sensu stricto*^3^) are not here considered within the family.

Additional references: Berrill^103^; Calder^53^; Cornelius^4^,^5^; Migotto^104^; Marques *et al.*^59^.

### Plumupheniida *taxon novum*

*N. lighti* and *H. mirabile* have unstable positions, but generally related to the clades Aglaopheniida *taxon novum* and Plumulariida *sensu novum*. Except for *N. lighti* and *H. mirabile*, Aglaopheniida *taxon novum* and Plumulariida *taxon novum* are characterized by uniseriate hydrothecae^5^,^25^,^26^,^28^,^45^,^46^ (Figs 1, 2, 3). Concerning the taxonomy, Plumupheniida has a different taxonomic comprehensiveness from Plumularioidea McCrady, 1859^3^ because *N. lighti* and *H. mirabile* are included in this clade based on multilocus data (similar situation for Aglaopheniida and Aglaopheniidae; see below and Table 3).

### *Nemalecium lighti* and *Hydrodendron mirabile* (Lineage 5 – L 5)

Traditionally considered as haleciids, *N. lighti* and *H. mirabile* were not recovered as a monophyletic group, or as closely related to Haleciida; therefore they are considered Plumupheniida *incertae sedis*. *Nemalecium lighti* is remarkable in having “swimming gonophores” with corpuscles that may be related to buoyancy^105^. This character is also found in some Plumulariidae (e.g., *Macrorhynchia philippina* cf. Gravier-Bonnet & Migotto^105^) and Aglaopheniidae (e.g., *Dentitheca bidentata* cf. Migotto & Marques^57^). The presence of “nematodactyls” in the ring of tentacles^106^ should be reanalyzed concerning its organization, because it may be homologous to nematophores^53^, which would corroborate the affinity of *Nemalecium* with the Plumupheniida. However, future analyses are still necessary to better investigate this question and to test the position of *N. lighti* with respect to the rest of Plumupheniida, especially concerning other traditional “haleciids”.

*Hydrodendron mirabile* is highly supported as sister group of Aglaopheniida, and shares the presence of nematophores and nematothecae with Aglaopheniida+Plumulariida *sensu novum*. Defense polyps, therefore, are corroborated as a possible synapomorphic condition of Plumulariida+Aglaopheniida+*H. mirabile* (Fig. 1, 2, 3), and not as a convergence (Leclère *et al.*^41^; corrobating a posterior result^45^). The taxonomic position of *H. mirabile*, however, has to be tested with the inclusion of additional molecular data for *H. gardineri* and other terminals of *Hydrodendron*, a genus with ca. 25 nominal species^2^.

Additional references: Rees & Vervoort^55^; Vervoort^56^; Cornelius^5^.

### Aglaopheniida *taxon novum*

Aglaopheniida has a high nodal support (see also references^41^,^42^,^45^). Some proposals are incongruent with our topology, because Gymnangiinae/Aglaopheniinae, and Aglaopheniini/Cladocarpini^107^ are not monophyletic due to the position of *Gymnangium gracilicaule* and *Lytocarpia* sp. Also, all traditional genera of Aglaopheniidae, except by *Macrorhynchia*, are not monophyletic in our proposal (Fig. 1; see also previous analyses^45^). Aglaopheniida is characterized by having paired lateral nematothecae partially fused to the hydrotheca, all nematothecae one-chambered and immovable^46^. Part of the taxon has gonothecae protected by phylactocarps or corbulae^5^.

Additional references: Gravier^108^; Svoboda & Cornelius^109^.

### Plumulariida *sensu novum*

Plumulariida *sensu novum* has a high nodal support and includes four families characterized by free nematothecae and bilateral symmetry of the hydrotheca in many species. Our cladogram shows Schizotrichidae as sister group of the rest of Plumulariida *sensu novum* (Figs 1, 2, 3), instead of sister group of Aglaopheniidae, as originally proposed^46^. Our proposal of the relationship among the other three families (viz., Halopterididae, Kirchenpaueriidae and Plumulariidae; Figs 1, 2, 3) is different from that previously proposed (cf. Leclère *et al.*^41^). The topology (Kirchenpaueriidae (Halopterididae, Plumulariidae)) (cf. Leclère *et al.*^45^; Peña Cantero *et al.*^46^) refutes the absence of cauline hydrothecae as synapomorphy for Plumulariidae, Kirchenpaueriidae and Aglaopheniidae^45^,^110^.

Kirchenpaueriidae is characterized by the absence of paired lateral nematothecae, and the presence of one to two mesial nematophores, naked or with reduced nematothecae, associated with each hydrothecae, usually also reduced^45^. Kirchenpaueriidae was traditionally considered as sister group of other Plumulariida due to the simple morphology of the nematothecae^90^, which are similar to those of Haleciidae^107^. However, our phylogeny does not corroborate this hypothesis, since Schizotrichidae has a basal position in Plumulariida *sensu novum*. Our phylogenetic relationships within Kirchenpaueriidae are as previously thought^111^: *Ventromma* is a monophyletic group (cf. Calder 1997^107^; Peña Cantero & Marques^112^) not synonymous of *Kirchenpaueria* (cf. Millard^23^; Bouillon^26^), which differs from *Ventromma* by the presence of naked sarcostyles^107^. In our topology, *Kirchenpaueria pinnata*, *Pycnotheca mirabilis* and species of *Oswaldella* define a monophyletic group. *Oswaldella* is a monophyletic group with high support and geographic distribution restricted to Antarctica and Patagonia shelf^112^,^113^,^114^. Further studies with molecular and morphological data should focus on the taxonomic affinities of *Halicornopsis*, *Ophinella* and *Wimveria*.

As stated above, Halopterididae becomes monophyletic with the exclusion of Schizotrichidae (viz., Peña Cantero *et al.*^46^) and the inclusion of *Polyplumaria flabellata*^5^,^107^,^110^. Amendments of the diagnosis based on morphological characters of Halopterididae was proposed by Peña Cantero *et al.*^46^. The genera traditionally considered as Halopterididae are not monophyletic (e.g. *Halopteris* Allman, 1877, cf. Leclère *et al.*^41^,^45^; Peña Cantero *et al.*^46^), and the shape of the colony as the main character to define their taxonomical limits might be questionable^110^. *Halopteris* is divided into several high-supported clades. *Halopteris carinata* Allman, 1877, type species of *Halopteris*, has different morphology from many of the species traditionally assigned to the genus (except for *Halopteris liechtensternii* (Marktanner-Turneretscher, 1890)), and the hypothesis that genera of Halopterididae are not monophyletic can be supported by morphology as well as molecular data (Dale Calder, pers. comm.). The position of species of *Antennella* on the topology (Figs 1, 2, 3) supports multiple origins of simplified colonies from the pinnate ancestral form^110^. Four genera (i.e., *Anarthoclada*, *Astrolabia*, *Nuditheca* and *Pentatheca*) have to be included in further analysis, especially because they have already been considered as Aglaopheniidae^30^, Halopterididae^107^,^110^ and Schizotrichidae^46^.

Plumulariidae is defined by our molecular data as sister group of Kirchenpaueriidae+Halopterididae, and it is also morphologically well defined^46^ (Figs 1, 2, 3). The taxonomy of the group, however, is troubled, because the genera are poorly defined and the species share many plastic characters^107^. Two well-supported clades were found in Plumulariidae: one includes the non-monophyletic genera *Dentitheca* and species of *Plumularia* (*Plumularia spiralis, Plumularia habereri* and *Plumularia insignis*); the other includes the 9 remaining *Plumularia* and 2 species of *Nemertesia* (*Nemertesia antennina* and *Nemertesia ventriculiformis*). In our phylogeny, *Nemertesia* is monophyletic and related to *Plumularia* species, a different relationship from previous study^115^. Overall, our results are congruent with the proposals of the current literature based on genetic data^41^,^45^,^46^.

Additional references: Millard^116^; Ansín Agís *et al.*^117^; Schuchert^118^.

### Evolutionary scenarios, life cycle and biogeography

Traditional evolutionary scenarios for Leptothecata may be either inadequate or speculative because of the lack of data, and sometimes because of a grounded phylogenetic framework. For instance, the hypothesis that erect, complex colonies without the medusa stage, such as those of Macrocolonia, would be an eventual energetic tradeoff for medusa suppression (discussed in Cornelius^31^), or the evolution of the “swimming gonophores”^119^, are not based on phylogenetic hypotheses.

However, one could say that the evolution of Leptothecata supports an interpretation of adaptive radiation^120^, both by having high rates of lineage diversification with morphological innovations (*e.g.*, colony structure), and diversified ecological properties (*e.g.*, benthic life cycle). On the other hand, extinction rates are difficult to estimate because of the limited fossil data, which severely restrict our knowledge on the historical distribution of ancestral lineages. Several alternative hypotheses may explain the rapid diversification of Leptothecata^45^,^121^,^122^. For instance, loss of the medusa stage, together with the reduction in the number of larvae, would result in phylopatric species with long-lived, sessile polyp stages (cf. Thiel & Gutow^123^).

Yet, when trying to explain potential advantages for presence or absence of the medusa stage, there are different interpretations. According to Leclère *et al.*^45^ (p. 510 our emphasis), the high number of convergent medusa losses/reductions observed among hydrozoans strongly suggests that getting rid of the pelagic stage is positively selected under some circumstances, leading Cornelius^121^ to emphasize a challenging paradox: “if medusa loss is advantageous, and if it can evolve easily, then why have not all recent forms dispensed with the medusa long ago?” However, Cornelius stated that the paradox condition was the loss of the “advantageous medusa stage”: “The widespread loss of the medusa stage is a paradox in view of its presumed advantage and undoubted complexity” (Cornelius^31^, p. 249, our emphasis).

Leclère *et al.*^45^ proposed an explanation for the evolution of different life cycles strategies^124^. In their scenario, species with simple colonies (such as those typical of Statocysta) would invest more in the reproductive and dispersive pelagic stage, with a benthic stage in which the planula quickly settles on living substrates (with less competition and greater tolerance to environmental stress). On the other hand, species with erect, complex colonies, without medusa (like those typical of Macrocolonia), would strongly compete for the substrate and have limited dispersal abilities, which could result in low gene flow and consequent local speciation. The (homoplastic) appearance of species with medusoids in lineages that lost the medusa could, in turn, be explained by high rates of extinction of clades that do not acquire reproductive or dispersive benefits, limiting the phenomenon of colonial brooding^44^. Thus, it is clear that these ideas presume greater dispersal ability of the pelagic adult stages (cf. Gibbons *et al.*^125^,^126^ with Cornelius^31^,^121^,^127^).

Our data support the interpretation of dispersal and rapid settling in Statocysta, in contrast with competitiveness in Macrocolonia. Because of that, stasis (cf. Eldredge *et al.*^128^) may have predominated in lineages with medusa stage^45^. However, mechanisms associated with the speciation process in Macrocolonia would not be just a product of dispersal limitations. Other factors, even combined with those previously discussed, may have been important in the exploration of new potential ecological niches, genetic diversity and diversification success (e.g., parental investment^129^). The first mechanism is the type of fertilization (Miller^130^; discussed in Corynidae and Obeliinae by Panteleeva^131^), which is widely debated in other modular organisms such as tunicates and bryozoans^132^,^133^. Overall, hydrozoans with the medusa stage in the life cycle spawn gametes directly to the environment (e.g., Laodicea), while those without the medusa stage retain the unfertilized egg, presenting internal fertilization, sometimes with incubation (e.g., some *Diphasia* species). The second mechanism is associated with the strength and robustness of the colonies, especially those with a main axis with erect monopodial growth and branches that are strengthened by the fusion of the hydrotheca to the perisarc^134^. The third mechanism concerns the increasing in modular and morphological complexity, reflected in the high occurrence of nematophores/nematothecae in complex and reproductive defense structures^135^,^136^, such as the phylactocarps and the corbulae in Aglaopheniida. In this case, the polyp stage would live longer than the medusa stage, reducing the periodicity of the life cycle and increasing the rate of anagenesis^31^.

All these mechanisms modulate levels of endemism in different biogeographic scenarios of Hydrozoa. Cornelius^31^,^137^ discussed several hypotheses in favor of the prevalence of the polyp stage as the main mechanism of dispersal in Hydrozoa, such as the high-energetic cost of producing a medusa stage, the small-size of the hydroid colonies that release medusa, the different realms occupied by the two stages and the difficulties of interchanges between them, the large-size of the colonies that do not produce medusa and tend to perennate, the brief life span of the medusa stage, and the low chances of two sexually different medusa to arrive at the same time in a long-distant area, to reproduce and to develop a new hydroid colony. In this context, we may hypothesize on one hand, that species with both polyp and medusa stages in the life cycle may have greater endemicity than those with only polyp or medusa stage in the life cycle^31^,^121^. On the other hand, species with both polyp and medusa stages in the life cycle are less common in remote areas of the deep sea and at distant places from the continental shelves^138^,^139^, as well as in regions with extreme environmental conditions (*e.g.*, Antarctic, Arctic, hydrothermal vents). However, few biogeographic studies dealing with the level of endemicity and/or areas of endemism in Hydrozoa species were fully carried out, with this being an open question to be better explored through different biogeographic methods. Another interesting biogeographical pattern in Macrocolonia is seen in Staurothecida *taxon novum*, where *Staurotheca*, *Symplectoscyphus* and *Antarctoscyphus* are widely represented in Antarctic and sub-Antarctic waters, supporting a unique phylogenetic history. In contrast, other genera of Sertulariida *taxon novum* (*e.g., Amphisbetia*, *Diphasia*, *Dynamena*, *Sertularia* and *Thyroscyphus*) have an antipolar distribution. Results of ecological niche modeling^140^ of Bougainvilliidae show that, probably, the potential niche (and correlated areas) of distribution of certain species is the sum of ecological restrictions of polyps and medusae, which strongly limits distribution. Therefore, the presence of both stages in the life cycle results in narrower potential areas to be colonized. We conclude that reductionism in scenarios of advantages and disadvantages of the medusa stage are oversimplifications of complex evolutionary processes. It is also evident that the evolution of the life-cycle strategies has consequences for the understanding of species distributions and biogeography.

### Final considerations

Evidences accumulated during the last decades on morphology and life cycle of leptothecates demand new interpretations on the evolution of the group, especially because traditional taxonomy is incongruent with the recent phylogenetic hypotheses. Monophyly of many traditional taxa (e.g., Sertulariidae) is not supported, although some patterns remain strong, such as orders Statocysta and Macrocolonia (Table 4). Therefore, we propose a phylogenetic taxonomical reorganization of Leptothecata down to the infraorder level, but considering only the well-supported clades. Although most of main traditional groups were sampled in this study, we emphasize that several taxa (e.g., from species up to the family level) have not been included yet in broad phylogenetic analyses, and those clades must be intregrated to this classificatory framework in the near future:

Superorder Leptothecata Cornelius, 1992

 Order Lafoeida Bouillon, 1984, *sensu novum*

 Order Laodiceida *taxon novum*

 Order Statocysta Leclère, Schuchert, Cruaud, Couloux, Manuel, 2009

  Suborder Campanulinida Bouillon, 1984, *sensu novum*

  Suborder Eirenida *taxon novum*

  Suborder Proboscoida Broch, 1910 *sensu novum*

   Infraorder Campanulariida Bouillon, 1984, *sensu novum*

   Infraorder Obeliida *taxon novum*

 Order Macrocolonia Leclère, Schuchert, Cruaud, Couloux, Manuel, 2009

  Suborder Staurothecida *taxon novum* [includes Staurothecidae *fam. nov.* and Symplectoscyphidae *fam. nov.*]

  Suborder Haleciida Bouillon, 1984 *sensu novum*

  Suborder Sertulariida *taxon novum* [includes Sertularellidae *fam. nov.*]

  Suborder Plumupheniida *taxon novum*

   Infraorder Aglaopheniida *taxon novum*

   Infraorder Plumulariida Bouillon, 1984 *sensu novum*

## Methods

### Taxonomic sampling and data

We generated new data for 74 nominal species of Leptothecata for a total of 220 terminals in the combined analysis. Forty-three additional species were used as outgroups (14 new sampled species incorporated in this study): 27 “Anthoathecata”, 7 Siphonophorae Eschscholtz, 1829 and 9 Trachylina species (“Trachylinae” in the original description from Haeckel, 1879, p. 233), totaling 263 hydrozoan species (Table S1).

We developed a workflow considering alternative protocols to extract total DNA of adequate molecular weight from a variety of tissues (from rich-mesoglea medusae and rich-chitin benthic colonies). Extractions were carried out with the Bio-Rad® INSTAGENE kit (based on the Chelex caotrophic component^141^) by using a volume (concentration) adjustment in the one-step extraction reaction. This is important for micro samples when the concentration had to be optimized. If the extraction proved to be difficult, or otherwise unsatisfactory, we then used the Agencourt® DNAdvance kit, in which total DNA is separated from the remaining lysed cell-tissues by means of magnetic particles with high affinity for double-stranded DNA. Quality control of both methods was carried out with a Thermo® NanoDrop 2000c spectrophotometer. Subsequently, we used typical PCR with the appropriate primers and annealing temperatures (*Tm*) for each molecular marker of interest (see Supplementary Tables S2, S3 online). Following the control for successful PCR on 1.5% agarose gel (stained with Biotium® GelRed), the double-stranded DNA PCR products were purified with the Agencourt® AMPure® kit (final purified DNA concentrations were checked with Thermo® NanoDrop 2000c). The sequencing reaction was carried out with the ABI Big Dye V3.1® kit, under standard conditions using the original PCR primers and *Tm* conditions. Final precipitation of DNA was carried out with ammonium acetate and ethanol^142^ and samples were sequenced in the Hitachi® ABI PRISM3100 genetic analyzer® (IQUSP). Chromatograms were mounted in GeneCodes Sequencer 4.6 and Geneious v5.4^143^. Controls for potential contamination error on the consensus sequences were made using BLAST on GenBank and local databases^144^. Final sequences were submitted to GenBank using Geneious v5.4^143^ (procedure summarized in Fig. S1).

Primers combinations used for each molecular marker amplified almost complete nuclear ribosomal sequences (small ribosomal subunit, or 18S, with ~1,800 pb; large ribosomal subunit, 28S, with ~3,200 pb) and part of the mitochondrial molecular marker (large mitochondrial subunit, or 16S, with ~600 pb). Sequence generation success, considering species and marker, together with voucher codes and collecting locations are summarized in Table S1 and Fig. S2. Along with our samples including as many species as possible for the ingroup, we sampled species from the rest of Hydrozoa as outgroups compiling a supermatrix dataset^145^ (Table S1). Names of species and higher taxonomic units were compared with those in WoRMS (World Hydrozoa Database^2^ genus-level taxonomy) and Bouillon^28^ (family-level taxonomy) with a few exceptions (Tables 1, 4, S3 and S4 for details of primers and protocols).

### Alignment, filtering strategies and molecular marker combinations

Alignments were performed using MAFFT v6^146^ using the E-INS-i algorithm. After the trimming of low quality regions from the 5′ and 3′ ends of the individual alignments, we used Aliscore to eliminate saturated and ambiguously aligned regions with two levels of filtration^147^. At the first filtration level (light), gaps are treated as missing data (similar to treatment of gaps in phylogenetic analysis, command -N) and an N was added to the acronym designating the resulting matrices (e.g., the marker 16S becomes 16S_N). We employed the default window size (i.e. 6 residues). In the next filtering level (heavy), the moving windows size was reduced to the minimum allowed by the algorithm and the resulting matrices received a “_Nrw4” suffix (e.g., 18S results in “18S_Nrw4”). Thus, each marker was present in three aligned data matrices: raw (that is, unedited, e.g., 18S), light filtering of hypervariable regions (e.g., 18S_N) and heavily filtered (e.g., 18S_Nrw4). Finally, concatenated data matrices were assembled using SequenceMatrix^148^ for every case (a full data matrix, named 16S18S28S, lightly filtered datamatrix16S18S28S_N and heavily filtered damatrix 16S18S28S_Nrw4), with matrices of up to 6,000bp (Tables 1 and 2). Thus, our datamatrix strategy permitted two different approximations, allowing us to obtain single and multilocus phylogenies, with and without filtration in each case, making up a total of 12 individual phylogenetic analyses (Table 2).

### Phylogenetic analysis and support

We carried out 250 ML tree searches on each data matrix using RAxML v8^149^ under default settings with a gamma distribution (generated by the data) and no invariable sites (GTR+GAMMA, following the software manual) in CIPRES portal^150^. For multilocus data matrices were partitioned by gene (Table 2). For support, considering parametric methods we used aLRT (approximate likelihood ratio test) and aBAYES (approximate transformation Bayes test), and for non-parametric methods we used the Bootstrap (BS) and SH-aLRT^48^,^151^. Bootstrap analyses for multilocus matrices were carried out in RAxML v7.3.0. (1,000 replicas in conditions similar to the original analysis). Values for aLRT, SH-aLRT and aBAYES were calculated for each optimal ML tree from RAxML (multilocus and individual) in PhyML 3.0.1 beta^152^ (parameters similar to those in RAxML). Anisimova *et al.*^48^ present a comprehensive comparison of the four measures of node support listed above with respect to their levels of sensitivity and specificity. Using simulated data, those authors concluded that the most widely used measure of node support (non-parametric bootstrapping - BS) is too conservative i.e., many nodes present in the tree that was used to simulate the analyzed data were not significantly supported by bootstrapping under the same model employed in the simulation. Here, we report as significantly supported not only the nodes whose BS values are equal or greater than 75%, but also the nodes that had support values equal or above the thresholds obtained by Anisimova *et al.*^48^ (aBAYES > 0.95; aLRT > 0.9; SH-aLRT > 0.85), even if BS nodal support was not significant (i.e. <0.75). Marker saturation conditions for 16S and 18S were evaluated with figures of transitions versus transversions in DAMBE^153^ (Fig. S12).

In order to detect rogue taxa, nonparametric bootstrap pseudo-replicates obtained from concatenated matrices were analyzed using the online version of RogueNaRok^154^ (http://rnr.h-its.org), under default parameters. The top ten rogue taxa were deleted from every combined data matrix and these new, “reduced” matrices were re-analyzed as the original ones. These analyses were used to assess rogue taxa’s potential impact on the broad phylogenetic picture of the Leptothecata and to pinpoint taxa with conflicting positions across topologies obtained from matrices with different levels of noise filtering (for further information see Supplementary Text, Supplementary Tables S4, S5, and Supplementary Figs S13–S15). Access to main data (Figshare database): http://dx.doi.org/10.6084/m9.figshare.1556316.

## Additional Information

**How to cite this article**: Maronna, M. M. *et al.* Towards a phylogenetic classification of Leptothecata (Cnidaria, Hydrozoa). *Sci. Rep.* **6**, 18075; doi: 10.1038/srep18075 (2016).

## Supplementary Material

Supplementary Information

## Figures and Tables

**Table 1:** Summary statistics for the data matrices corresponding maximum likelihood trees.

Analysis	16S18S28S	16S18S28S_N	16S18S28S_Nrw4	16S	16S_N	16_Nrw4
Terminal taxa	263	**263**	263	254	254	254
Total sites	6,254	**5,608**	4,548	647	582	452
Conserved sites	2,522	**2,463**	2,413	118	117	112
Variable sites	3,677	**3,131**	2,132	526	465	340
Informative sites (parsimony)	2,861	**2,478**	1,551	462	417	300
Singleton sites	757	**637**	581	61	46	40
Gaps and missing data (%)	38.55	**33.02**	28.94	15.97	8.72	3.64
Maintained sites (Aliscore, in %)		**89.67**	72.72		89.95	69.86
Log-likelihood (RAxML)	−169046.88	−**151027.90**	−71717.62	−39040.32	−36397.24	−19898.97
Freq A C G T (PhyML)	0.28 0.19	**0.28 0.19**	0.29 0.19	0.40 0.12	0.40 0.12	0.38 0.13
	0.26 0.26	**0.26 0.25**	0.26 0.25	0.15 0.31	0.15 0.31	0.17 0.30
Invariant proportion: MEGA (Gamma+I. “use all sites”)	~36%	**~39%**	~43%	~16%	~18%	~21%
Parameters: (alpha PhyML) [Gamma MEGA]	(0.344) [0.647]	**(0.325) [0.651]**	(0.294) [0.548]	(0.424) [0.587]	(0.425) [0.571]	(0.6361) [0.443]
**Analysis**	**18S**	**18S_N**	**18S Nrw4**	**28S**	**28S N**	**28S Nrw4**
Terminal taxa	207	207	207	181	181	181
Total sites	1,955	1,802	1,566	3,652	3,224	2,530
Conserved sites	888	862	848	1,516	1,484	1,453
Variable sites	1,050	940	718	2,101	1,726	1,074
Informative sites (parsimony)	782	708	506	1.617	1.353	745
Singleton sites	262	229	212	434	362	329
Gaps and missing data (%)	14.40	7.93	2.86	20.39	12.67	7.31
Maintained sites (Aliscore, in %)		92.17	80.10		88.28	69.28
Log-likelihood (RAxML)	−37348.89	−35029.30	−21383.44	−91649.78	−78681.31	−29528.53
Freq A C G T (PhyML)	0.26 0.19	0.26 0.19	0.27 0.19	0.26 0.21	0.26 0.21	0.27 0.20
	0.26 0.26	0.26 0.26	0.26 0.26	0.28 0.24	0.28 0.23	0.27 0.23
Invariant proportion: MEGA (Gamma+I; “use all sites”)	~38%	~40%	~42%	~38%	~42%	~44%
Parameters: (alpha PhyML) [Gamma MEGA]	(0.296) [0.601]	(0.296) [0.595]	(0.273) [0.561]	(0.319) [0.633]	(0.295) [0.643]	(0.247) [0.530]

(multilocus: 16S1828S, 16S18S28S_N, 16S18S28S_Nrw4, single locus: 16S, 16S_N, 16S_Nrw4, 18S, 18S_N, 18S_Nrw4, 28S, 28S_N, 28S_Nrw4). The log-likelihoods were calculated in RAxML under the GTR+GAMMA model. Nucleotide frequencies and proportion of invariant sites calculated in PhyML and MEGA (together with the estimate of the alpha and gamma parameters in both programs). Cells were left empty when non-applicable.

**Table 2:**
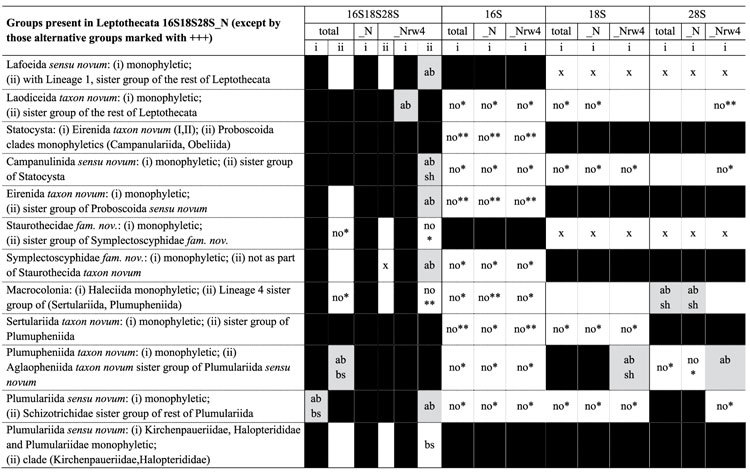
Results of nodal support for the main groups of Leptothecata in 16S18S28S_N (Fig. 1) and all topologies.

Black cells indicate nodes significantly supported by at least 3 of 4 support methods for multilocus, and 3 of 3 for single locus analysis; gray cells indicate that one or two branch support methods are significant (ab = aBAYES; sh = SH_aLRT, bs = Bootstrap); white cells represent unsupported clades considering all methods. Single asterisk (*) indicates para- or polyphyletic taxa, with small patristic distances among terminals; double asterisks (**) indicate large patristic distances (see figures and text for details). Symbol x indicates non-applicable (see main text and Supplementary Table S1 online). Rogue taxa and lineages with unstable placements are not considered, except where detailed.

**Table 3:** Taxonomic proposal for Leptothecata compared to Bouillon^28^, Cornelius^4^,^5^ and the WoRMS database^2^.

Group	Code	Mono (Y/N)?	Bouillon 1995	WoRMS 2015	Cornelius^4^,^5^	P/M
LEPTOTHECATA		**Y**	Subclass; Fleming, **1828** (=Thecatae)	Order; Cornelius, 1992	Subclass; Cornelius, 1992(=Leptothecatae)	P/M
PROBOSCOIDA	P	N	Order; Broch, 1909	Suborder; Broch, 1910 (not accepted: “polyphyletic assemblage”)	Order; Broch, 1910	P
Campanulariida	P	N	Suborder; Johnston, **1836**	absent	Suborder; Bouillon 1984	
Campanularioidea	P	N	Johnston, **1836**	absent	Johnston, **1837**	
Phialuciidae + Campanulariidae	P_1	X	absent	absent	absent	P
Phialuciidae	P_1.1	NS	Kramp, 1955	=	=	M
Campanulariidae	P_1.2	N	Johnston, **1836**	=	Johnston, **1837**	M
Bonneviellidae	P_2	N	Broch, 1909	=	Order; Broch, 1910	P
CONICA	C	N	Order; Broch, 1909	Genus; Broch, 1910 (not accepted: “polyphyletic assemblage”)	Order; Broch, 1910	P
Campanulinida + Lafoeida	Ce_1	N	absent	absent	absent	P
Lafoeida	C_1	N	Suborder; Agassiz, **1865**	absent	Suborder; Bouillon 1984	P
Lafoeoidea	C_1	N	Agassiz, 1865	absent	=	
Clathrozoidae	C_1.1	NS	Stechow, 1921	=	=	P
Hebellidae	C_1.2**	N	absent (part of Lafoeidae)	Fraser, 1912	=	P/M
Lafoeidae	C_1.3	N	Agassiz, **1865**	Hincks, **1868**	=	P
Campanulinida	C_2	N	Suborder; Hincks, **1868**	absent	Suborder; Bouillon, 1984	P
Campanulinoidea	C_3 & C_11**	N	Hincks, **1868**	absent	=	**
Campanulinidae	C_3	N	Hincks, **1868**	=	**=**	M
Campanulinida - Campanulinidae	C_4	N	absent	absent	absent	M
Laodiceoidea	C_5	Y*	Browne, 1907	absent	Agassiz, **1862**	M
Laodiceidae	C_5.1	Y*	Browne, 1907	Agassiz, **1862**	= WoRMS	P/M
Tiarannidae	C_5.2	N	Russell, 1940	Russell, 1950	=	P/M
Dipleurosomatoidea + Mitrocomoidea + Lovenelloidea + Eirenoidea + Campanulinoidea*	C_6	N	absent	absent	absent	M
Dipleurosomatoidea	C_7	X	Boeck, **1866**	absent	=	M
Orchistomatidae	C_7.1	NS	Bouillon, 1984 (=Orchistomidae)	=(Orchistomidae: “incorrect formation of family name”)	Bouillon, 1984 (recognized as “Orchistomidae”)	M
Dipleurosomatidae	C_7.2	NS	Boeck, **1866**	Russell, 1953	=	P/M
Melicertidae	C_7.3	X	Agassiz, **1862**	=	**=**	P/M
Mitrocomoidea + Lovenelloidea + Eirenoidea + Campanulinoidea*	C_8	N	absent	absent	absent	M
Mitrocomoidea	C_9	N	Torrey, 1909	absent	Haeckel, **1879**	M
Mitrocomidae	=C_9	N	Torrey, 1909	Haeckel, **1879**	= WoRMS	
Lovenelloidea + Eirenoidea + Campanulinoidea*	C_10	N	absent	absent	absent	M
Campanulinoidea* (= Campanulinoidea - Campanulinidae)	C_11	N	absent	absent	absent	M
Malagazziidae + Aequoreidae	C_12	N	absent	absent	absent	M
Malagazziidae	C_12.1	X	Bouillon, 1984	=	=	M
Aequoreidae	C_12.2	N	Eschscholtz, **1829**	=	**=**	M
Blackfordiidae + Phialellidae + Sugiuridae	C_13	N	absent	absent	absent	M
Blackfordiidae	C_13.1	X	Bouillon, 1984	=	not sampled	P/M
Phialellidae	C_13.2	X	Russell, 1953	=	=	P/M
Sugiuridae	C_13.3	X	Bouillon, 1984	=	not sampled	P/M
Lovenelloidea + Eirenoidea	C_14	N	absent	absent	absent	M
Lovenelloidea	C_15	N	Russell, 1953	absent	=	M
Cirrholoveniidae	C_15.1	NS	Bouillon, 1984	=	=	P
Lovenellidae	C_15.2	N	Russell, 1953	=	=	P
Eucheilotidae	C_15.3	N	Picard, 1958	not accepted (synonymous of Lovenellidae)	Bouillon, 1984	P
Eirenoidea	C_16	N	Haeckel, **1879**	absent	=	M
Eirenidae	C_16	N	Haeckel, 1879**	=	=	
Haleciida + Plumulariida	Ce 2	N	absent	absent	absent	P
Haleciida	Ce_3	N	Suborder; Hincks, **1868**	absent	Suborder; Bouillon, 1984	P
Halecioidea	Ce_3	N	Hincks, **1868**	absent	=	P
Haleciidae	Ce_3	N	Hincks, **1868**	=	=	
Plumulariida	Ce_4**	N	Hincks, **1868**	absent	Suborder; Bouillon, 1984	
Sertularioidea	Ce_5**	N	Lamouroux, **1812**	absent	=	P
Sertulariidae	Ce_5.1	N	Lamouroux, **1812**	=	**=**	P
Syntheciidae	Ce_5.2	X	Marktanner-Turneretscher, 1890	=	**=**	P
Plumularioidea	Ce_6	**Y**	Hincks, **1868**	McCrady, 1859***	Agassiz, **1862**	P
Aglaopheniidae	Ce_6.1	**Y**	Broch, 1918	Marktanner-Turneretscher, **1890**	Agassiz, **1862**	P
Kirchenpaueriidae	Ce_6.2	**Y**	Millard 1962	Stechow, 1921; Marktanner-Turneretscher, 1890***	Considered Subfamily Kirchenpaueriinae Stechow, 1921 (in Plumulariidae)	P
Halopterididae	Ce_6.3	N	Millard, 1962	=	**Considered Subfamily Halopteriinae Millard, 1962 (in Plumulariidae)	P
Plumulariidae	Ce_6.4	**Y**	Hincks, **1868**	Agassiz, **1862**; McCrady, 1859***	Agassiz, 1862**	P

The column “Mono” reports whether the corresponding group is monophyletic according to our working hypothesis. Asterisks indicate monophyly but for one terminal, X indicate that only one representative terminal of the group was sampled (therefore we are unable to fully ascertain the phylogenetic status of the group in our analysis) and NS indicates that no representative of the group was sampled. Taxonomic status and authorship according to the sources above are listed under respective columns. The symbol (=) indicates that taxonomy and authorship are identical to Bouillon^28^. Groups proposed in XIX century are in boldface. “P/M” indicates if original proposal was mainly based on polyp (P), medusae (M) characters or both (P/M). New taxonomic proposal and differences from Bouillon^28^ and WoRMS^2^ are also presented in Fig. 4, Table 4 and Table S1.

**differences in the contents considering original proposal from Bouillon^28^.

***=considering Calder^107^.

**Table 4:** Clades in the working hypothesis (Fig. 1) compared with those of Bouillon^28^.

Principal clades	Bouillon (1995)	Represented Groups or Genera and/or // Species
Order Lafoeida *sensu novum*	C_1.3 Ce_5.2*	*Acryptolaria conferta*, *Lafoea dumosa*, *Hincksella* sp.
Order Laodiceida *taxon novum*	C_5*	*Laodicea undulata*, *Modeeria rotunda*, *Melicertissa* sp., *Stegopoma plicatile*
Order Statocysta	C_3* C_5.2*M C_8* P_1.2* P_2*	(Campanulinida *sensu novum* (Eirenida *taxon novum, Proboscoida sensu novum*))
Suborder Campanulinida *sensu novum*	C_3* C_9* C_13.2* C_5.2*	*Mitrocomella*, Campanulina* //* Calycella syringa*, *Phialella quadrata*, *Tiaropsidium kelseyi*, *Tiaropsis multicirrata*
Suborder Eirenida *taxon novum*	C_10*	(Eirenids I, Eirenids II)
Eirenids I	C_12.1* C_16* C_12.2 C_13.3 C_13.1	Aequorea*, Eirene* //* Blackfordia virginica*, *Eutonina indicans*, *Octophialucium indicum*, *Rhacostoma atlanticum*, *Sugiura chengshanense*
Eirenids II	C_16* C_15.3*	Eucheilota*, Eutima*, *Helgicirrha*//*Hydranthea margarica*, *Eugymnanthea japonica*
Suborder Proboscoida *sensu novum*	P_1.2* P2* C_15.2* C_15.3*	(Campanulariida *taxon novum*, Obeliida *taxon novum*)
Infraorder Campanulariida *taxon novum*	P_1.2* P_2*	Bonneviella*, Campanularia*, Orthopyxis*//*Rhizocaulus verticillatus*, *Silicularia rosea*
Infraorder Obeliida *taxon novum*	C_15.2* C_15.3* P_1.2*	Laomedea*, Obelia*, Clytia*// Eirene brevistylus**, Gonothyraea loveni
Order Macrocolonia	Ce_2*	(Staurothecida *taxon novum* (Haleciida *sensu novum* (Sertulariida *taxon novum*, Plumupheniida *taxon novum*)))
Suborder Staurothecida *taxon novum*	Ce_5*	*Staurotheca*, *Antarctoscyphus*, Symplectoscyphus*
Staurothecidae *fam. nov.*	Ce_5.2*	*Staurotheca*
Symplectoscyphidae *fam. nov.*	Ce_5.1*	*Antarctoscyphus*, Symplectoscyphus*
Suborder Haleciida *sensu novum*	Ce_3*	*Halecium*
Suborder Sertulariida *taxon novum*	Ce_5*	(Sertularellidae *fam. nov.*(Thyroscyphidae, Sertulariidae *sensu novum*))
Sertularellidae *fam. nov.*	Ce_5*	*Sertularella*//Symplectoscyphus turgidus**
Thyroscyphidae	Ce_5*	Thyroscyphus*// *Sertularelloides cylindritheca*
Sertulariidae *sensu novum*	Ce_5*	*Abietinaria*, *Amphisbetia*, *Diphasia*, Dynamena*, Sertularia*, *Thuiaria*//*Hydrallmania falcata, Idiellana pristis*, *Salacia desmoides*
Suborder Plumupheniida *taxon novum*	Ce_6* Ce_3*	(Aglaopheniida *taxon novum*, Plumulariida *sensu novum*)
Infraorder Aglaopheniida *taxon novum*	Ce_6.1	Aglaophenia*, Gymnangium*, Lytocarpia*, *Macrorhynchia*//*Cladocarpus integer*
Infraorder Plumulariida *sensu novum*	Ce_6.2 Ce_6.3*Ce_6.4	(Schizotrichidae (Kirchenpaueriidae, Halopterididae, Plumulariidae))
Schizotrichidae	Ce_6.3*	*Schizotricha*
Kirchenpaueriidae	Ce_6.2	*Oswaldella*//*Kirchenpaueria pinnata*, *Pycnotheca mirabilis*, *Ventromma halecioides*
Halopterididae	Ce_6.3*	Antennella*, Halopteris*//*Monostaechas quadridens*, *Polyplumaria flabellata*
Plumulariidae	Ce_6.4*	*Nemertesia*, Plumularia*//*Dentitheca bidentata*

Single asterisks (*) denote non-monophyletic groups in Fig. 1 (topology 16S18S28S_N), considering traditional taxonomy from WoRMS database; double asterisks (**) indicate *dubious information so far*. Because of their unstable or poorly supported placements, lineages and rogue taxa were not included. For a phylogenetic description of differences between our results and Bouillon^28^, see Fig. 4.
