# Supplementary material for: Towards a phylogenetic classification of Leptothecata (Cnidaria, Hydrozoa)
Source: Sci Rep. 2016 Jan 29;6:18075. doi: 10.1038/srep18075 (PMC4731775; doi:10.1038/srep18075)
Supplement: Supplementary Information [file srep18075-s1.pdf]

**Title**: Towards a phylogenetic classification of Leptothecata (Cnidaria, Hydrozoa)

Maximiliano M. Maronna[1*], Thaís P. Miranda[1], Álvaro L. Peña Cantero[2], Marcos S. Barbeitos[3], and Antonio C. Marques[1,4]

1 Departamento de Zoologia, Instituto de Biociências, Universidade de São Paulo

Rua do Matão Trav. 14, 101, 05508-090, São Paulo, Brazil

2 Instituto Cavanilles de Biodiversidad y Biología Evolutiva, Departamento de Zoología

Universidad de Valencia, Valencia, Spain

3 Departamento de Zoologia, Caixa Postal 19020, Universidade Federeal do Paraná, 81531-990, Curitiba, PR, Brazil

4 Centro de Biologia Marinha, Universidade de São Paulo, São Sebastião, Brazil

*Corresponding author: maxmaronna@gmail.com

keywords: Leptothecata, phylogenetic systematics, taxonomy, macroevolution.

**We use this section to present additional information, results (figures and tables) and the "Rogue taxa analysis".**

**Main text and Results:**

**> Outgroups**

Overall, the support for the node defining the sister group was low, with the exception of the lineage Filifera IV+*Hydractinia* sp., in the 16S18S28S_Nw4 analysis[1] (Fig. 3; see also results of the 28S_N and 28S_Nrw4 analyses: Figs. S10, S11). The phylogenies for the 16S data had an artifact caused by long-branch attraction, causing Leptothecata to appear non-monophyletic (e.g., Fig. S3).

**> Statocysta**

Campanulinida *sensu novum* is also retrieved as a sister group of Eirenida *taxon novum* + Proboscoida *sensu novum* in the analyses of the unfiltered data matrix (16S18S28S), but it becomes sister clade to Eirenida *taxon novum* under the high filtering approach (16S18S28S_Nrw4) (Figs. 2-3).

**> Macrocolonia**

>> The nodal support of Staurothecidae fam. nov., Sertulariida *taxon novum*, Thyroscyphidae Stechow, 1920, and Sertulariidae Lamouroux, 1812 decreases with filtering (Figs. 1-3, Figs. S6-8).

>> In all the combined results, the basal lineages of Plumulariidae have high support values (Figs. 1-3; Table 2).

>> In the transition:transversion (ts:tv) frequency analysis for 16S and 18S, data matrices are similar, although the types of changes vary. In 16S, the relative frequencies of the nucleotides and the ts:tv relationship is very similar in filtered and unfiltered matrices (Fig. S12; Table 1), but frequencies change and bias in nucleotide frequencies decreases (Fig. S10; Table 1). In the 18S dataset, nucleotide frequencies are essentially constant (regardless of filtering level) in all matrices. The ts:tv ratio is similar for all but the highest filtering level (Fig. S10; Table 2). The 28S ts:tv relationships could not be calculated using DAMBE[153] due to buffer limitations.

**Supplemental Figures**

Figure 1 – Flow chart illustrating the strategy employed to acquire the genetic data information, emphasizing those changes we had to make (red dotted lines) and subsequent procedures after positive results (solid green lines). See Supplementary Tables 3 and 4.

Figure 2 – Map with the global distribution of the analyzed specimens. Red dots indicate the approximate geographic locations of specimens whose sequences were obtained from GenBank, and black dots indicate the geographic location of samples collected during this study (see Table S1). Map created using QGIS 2.10.1 http://qgis.org/ (Data source from DIVA-GIS website http://www.diva-gis.org/Data).

Figure 3 – Maximum-likelihood cladogram (unfiltered, 16S) for the Leptothecata. Three support values (two parametric, above the branch -aBAYES, aLRT-; one non-parametric, beneath the branch -SH-aLRT-) for each branch. Support values are integral numbers (1, 0) or decimals (no leading zroes). Basic taxonomy (species and genera) from WoRMS[2].

Figure 4 - Maximum-likelihood cladogram (low filtering, 16S) for the Leptothecata. Notations as in Fig. 3.

Figure 5 - Maximum-likelihood cladogram (high filtering, 16S) for the Leptothecata. Notations as in Fig. 3.

Figure 6 - Maximum-likelihood cladogram (unfiltered, 18S) for the Leptothecata. Notations as in Fig. 3.

Figure 7 - Maximum-likelihood cladogram (low filtering, 18S) for the Leptothecata. Notations as in Fig. 3.

Figure 8 - Maximum-likelihood cladogram (high filtering, 18S) for the Leptothecata. Notations as in Fig. 3.

Figure 9 - Maximum-likelihood cladogram (unfiltered, 28S) for the Leptothecata. Notations as in Fig. 3.

Figure 10 - Maximum-likelihood cladogram (low filtering, 28S) for the Leptothecata. Notations as in Fig. 3.

Figure 11 - Maximum-likelihood cladogram (high filtering, 28S) for the Leptothecata. Notations as in Fig. 3.

Figure 12 – Susbstitution patterns of the 16S and 18S markers. Matrices were unfiltered (16S, 18S, 28S), minimally filtered (16S_N, 18S_N, 28S_N) and high filtering (16S_Nrw4, 18S_Nrw4, 28S_Nrw4). Transitions (s) and transversions (v) are plotted against genetic distance calculated using the GTR model of evolution, including all alignment sites. (A) 16S, (B) 16S_N, (C) 16S_Nrw4, (D) 18S, (E) 18S_N, (F) 18S_Nrw4. Substitution patterns for the 28S marker could not be computed in the program DAMBE[153] because of buffer limitations.

Figure 13 - Maximum-likelihood cladogram from rogue-pruned, unfiltered, concatenated matrix (16S18S28S). Notations as in Fig. 3.

Figure 14 - Maximum-likelihood cladogram from rogue-pruned, lightly filtered, concatenated matrix (16S18S28S_N). Notations as in Fig. 3.

Figure 15 - Maximum-likelihood cladogram from rogue-pruned, highly filtered, concatenated matrix (16S18S28S_Nrw4). Notations as in Fig. 3.

Supplementary Table 1 – Species analyzed in the Leptothecata study. "Taxa/Category" refers to the groups, lineages and rogue taxa emphasized in the Results (Figure 1, 16S18S28S_N topology); order of genera and species does not imply monophyly. "Family (WoRMS)" indicates the family to which the genus or species belongs according to WoRMS[2]. The column “nr” indicates total number of analyzed samples. The column "S_s" refers to the number of species considered by WoRMS for that genus (“S”) and number of species analyzed in our study for that genus (“s”). "16S ", "18S " and "28S" present GenBank accession numbers for each molecular marker; sequences generated in this study are in bold face. Molecular markers for a certain species obtained from different vouchers are highlited using a gray background (GenBank code, voucher and Country/Region). Code "no ID" refers to those sequences where information is missing (i.e., voucher and/or country/region). Species cells with symbol * are those species considered as potential taxonomic errors (see main text for details). Species cells with symbol ** are those species with different taxonomic identification with GenBank or WoRMS (September 2015).

| Taxa / Category | Family (WoRMS) | nr | Species | S_s | 16S info | 18S info | 28S info | Voucher | Country/Region |
|---|---|---|---|---|---|---|---|---|---|
| Subclass Leptothecata | | | | | | | | | |
| Order Lafoeida *sensu novum* | | | | | | | | | |
| | Syntheciidae | 1 | *Hincksella* sp. | 11_1 | **KT757182** | | | **KU:570** | **Japan** |
| | Lafoeidae | 2 | *Lafoea dumosa* (Fleming, 1820) | 11_1 | FN424137 | **KT757183-4** | EU305520 | **lem_27A** | Antarctica |
| | | | | | | | | MHNG:INVE29952 | Iceland |
| | | 3 | *Acryptolaria conferta* (Allman, 1877) | 39_1 | AM887981 | | | no ID | Gulf of Cadiz |
| Lineage 1 (L 1) | Melicertidae | 4 | *Melicertum octocostatum* M. Sars, 1835 | 2_1 | FJ550510 | FJ550595 | FJ550451 | MHNG:INVE48744 | Norway |
| | Campanulinidae | 5 | *Stegella lobata* (Vanhöffen, 1910) | 1_1 | FN424119 | AY789778 | | no ID | Antarctica |
| | | | | | | | | 641AN | Antarctica |
| Order Laodiceida *taxon novum* | | | | | | | | | |
| | Laodiceidae | 6 | *Laodicea undulata* (Forbes & Goodsir, 1853) | 10_1 | FJ550471 | | FJ550390 | MHNG:INVE31753 | France |
| | | 7 | *Melicertissa* sp. | 7_1 | AY512515 | AF358075 | AY920798 | no ID | Guam Iceland |
| | | | | | | | | no ID | no ID |
| | Tiarannidae | 8 | *Modeeria rotunda* (Quoy & Gaimard, 1827) | 2_1 | FJ550476 | FJ550540 | FJ550396 | MHNG:INVE32967 | France |
| | | 9 | *Stegopoma plicatile* (Sars, 1863) | 5_1 | FJ550513 | FJ550598 | FJ550454 | MHNG:INVE48755 | Norway |
| Lineage 2 (L 2) | Hebellidae | 10 | *Anthohebella parasitica* (Ciamician, 1880) | 6_1 | AY787918 | EU272603 | EU272545 | MHNG:INVE29762 | Spain |
| | | 11 | *Hebella scandens* (Bale, 1888) | 14_2 | **KM822777** | | | **lem_S44** | Brazil |
| | | 12 | *Hebella venusta* (Allman, 1877) | | FJ550496 | FJ550574 | FJ550431 | MHNG:INVE35476 | Honduras |
| | Laodiceidae | 13 | *Staurodiscus gotoi* (Uchida, 1927) | 16_1 | FJ550472 | FJ550535 | FJ550391 | MHNG:INVE33467 | New Zealand |
| Order Statocysta | | | | | | | | | |
| Suborder Campanulinida *sensu novum* | | | | | | | | | |
| | Campanulinidae | 14 | *Calycella syringa* (Linnaeus, 1767) | 5_1 | AY789833 | AY789776 | FJ550372 | 664MA | USA |
| | | | | | | | | no ID | France |
| | | 15 | *Campanulina panicula* G.O. Sars, 1874 | 7_2 | FJ550511 | FJ550596 | FJ550452 | MHNG:INVE48748 | Norway |
| | | 16 | *Campanulina pumila*** (Clark, 1875) | | AY789834 | AY789777 | | 781MA | USA |
| | Mitrocomidae | 17 | *Mitrocomella brownei* (Kramp, 1930) | 9_2 | | FJ550521 | FJ550374 | no ID | France |
| | | 18 | *Mitrocomella niwai* Bouillon & Barnett, 1999 | | FJ550473 | FJ550536 | FJ550392 | no ID | New Zealand |
| | Phialellidae | 19 | *Phialella quadrata* (Forbes, 1848) | 11_1 | FJ550474 | FJ550537 | FJ550393 | MHNG:INVE33466 | New Zealand |
| | Tiaropsidae | 20 | *Tiaropsidium kelseyi* Torrey, 1909 | 7_1 | AY512517 | AF358079 | EU305537 | AGC1033 | USA |
| | | 21 | *Tiaropsis multicirrata* M. Sars, 1835 | 2_1 | FJ550468 | FJ550531 | FJ550386 | no ID | Iceland |
| Suborder Eirenida *taxon novum* | | | | | | | | | |
| Eirenids I | | | | | | | | | |
| | Aequoreidae | 22 | *Aequorea coerulescens* (Brandt, 1835) | 23_5 | **KT266599** | | | no ID | no ID |
| | | 23 | *Aequorea conica* Browne, 1905 | | HM053552 | | | IOCAS:36021 | no ID |
| | | 24 | *Aequorea floridana* (Agassiz, 1862) | | | | EU305506 | AGC333 | Bahamas |
| | | 25 | *Aequorea forskalea*** Péron & Lesueur, 1810 | | AY512518 | AF358076 | EU305505 | AGC1075 | USA |
| | | | | | | | | no ID | no ID |
| | | 26 | *Aequorea victoria* (Murbach & Shearer, 1902) | | EU305469 | AF358077 | AY920799 | no ID | no ID |
| | | | | | | | | KUNHM2867 | no ID |
| | | 27 | *Rhacostoma atlanticum*** L. Agassiz, 1851 | 1_1 | **KT266638** | **KT722425** | **KT757181** | **lem_S65** | Brazil |
| | Blackfordiidae | 28 | *Blackfordia virginica* Mayer, 1910 | 3_1 | **KT266605** | **KT722387** | **KT757145** | **lem_S42** | Brazil |
| | Eirenidae | 29 | *Eirene hexanemalis* (Goette, 1886) | 23_5 | FJ418648 | | | LMZ-200612-2 | no ID |
| | | 30 | *Eirene kambara* (Goette, 1886) | | FJ418649 | FJ418670 | | LMZ-200704 | no ID |
| | | 31 | *Eirene lacteoides* Kubota & Horita, 1992 | | FJ418650 | FJ418671 | | LMZ-200712 | no ID |
| | | 32 | *Eirene pyramidalis* (Agassiz, 1862) | | FJ418652 | FJ418673 | | LMZ-200705 | no ID |
| | | 33 | *Eirene viridula* (Péron & Lesueur, 1809) | | FJ550502 | FJ550588 | FJ550445 | no ID | France |

| | | | | | | | | | |
|---|---|---|---|---|---|---|---|---|---|
| | | 34 | *Eutonina indicans* (Romanes, 1876) | 2_1 | **KT266615** | | **KT757189-90** | **lem_S131** | Brazil |
| | Malagazziidae | 35 | *Octophialucium indicum* Kramp, 1958 | 12_1 | AY787897 | EU272626 | EU272577 | MHNG:INVE29970 | New Zealand |
| | Sugiuridae | 36 | *Sugiura chengshanense* (Ling, 1937) | 2_1 | HM053546 | | | IOCAS:36023 | no ID |
| Eirenids II | | | | | | | | | |
| | Eirenidae | 37 | *Eugymnanthea japonica* (Yamada, 1950) | 3_1 | AY285162 | FJ418674 | | no ID | no ID |
| | | | | | | | | no ID | Japan |
| | | 38 | *Eutima curva* Browne, 1905 | 21_5 | FJ550514 | FJ550599 | FJ550455 | MHNG:INVE33468 | New Zealand |
| | | 39 | *Eutima gegenbauri* (Haeckel, 1864) | | FJ550515 | FJ550600 | FJ550456 | MHNG INVE31748 | France |
| | | 40 | *Eutima krampi* Guo, Xu & Huang, 2008 | | FJ418653 | FJ418675 | | LMZ-200606-1 | no ID |
| | | 41 | *Eutima levuka* (Agassiz & Mayer, 1899) | | FJ418654 | FJ418676 | | LMZ-200707 | no ID |
| | | 42 | *Eutima sapinhoa* Narchi & Hebling, 1975 | | | EU305493 | EU305515 | AL548 | no ID |
| | | 43 | *Helgicirrha brevistyla* Xu & Huang, 1983 | 11_2 | FJ418655 | FJ418677 | | LMZ-200606-2 | no ID |
| | | 44 | *Helgicirrha malayensis* (Stiasny, 1928) | | FJ418645 | FJ418668 | | LMZ-200612-3 | no ID |
| | Lovenellidae | 45 | *Eucheilota maculata* Hartlaub, 1894 | 20_2 | **KT266613** | **KT722394** | **KT757148** | **lem_S132** | Brazil |
| | | 46 | *Eucheilota menoni* A. Agassiz, 1862 | | FJ550493 | FJ550570 | FJ550427 | MHNG:INVE33457 | New Zealand |
| | | 47 | *Hydranthea margarica* (Hincks, 1862) | 1_1 | DQ855932 | FJ550567 | FJ550424 | no ID | Spain |
| Suborder Proboscoida *sensu novum* | | | | | | | | | |
| Infraorder Campanulariida *sensu novum* | | | | | | | | | |
| | Bonneviellidae | 48 | *Bonneviella regia* (Nutting, 1901) | 10_4 | AY789805 | AY789740 | | 818AS | USA |
| | | 49 | *Bonneviella* sp. 2 819AS | | AY789806 | AY789741 | | 819AS | USA |
| | | 50 | *Bonneviella* sp. 3 830AS | | AY789807 | AY789742 | | 830AS | USA |
| | | 51 | *Bonneviella* sp. 4 839AS | | AY789808 | AY789743 | | 839AS | USA |
| | Campanulariidae | 52 | *Campanularia hincksii* Alder, 1856 | 35_3 | AY789794 | AY789729 | | 37IT | Italy |
| | | 53 | *Campanularia* sp. | | FN424118 | **KT722389** | **KT757187-8** | **lem_17A** | Antarctica |
| | | 54 | *Campanularia volubilis* (Linnaeus, 1758) | | AY789804 | AY789739 | | USNM:1106166 | USA |
| | | 55 | *Orthopyxis crenata*** (Hartlaub, 1901) | 19_4 | FJ550466 | | FJ550383 | no ID | New Zealand |
| | | 56 | *Orthopyxis everta* (Clark, 1876) | | AY789793 | AY789728 | | 66IT | Italy |
| | | 57 | *Orthopyxis mianzani* Cunha, Genzano & Marques, 2015 | | **KT266632** | **KT722415** | **KT757151** | **MZUSP:4107** | Brazil |
| | | 58 | *Orthopyxis sargassicola* (Nutting, 1915) | | **KT266633** | **KT722416** | **KT757155** | **MZUSP:4247** | Brazil |
| | | 59 | *Rhizocaulus verticillatus* (Linnaeus, 1758) | 2_1 | AY789803 | AY789738 | | USNM:1106183 | USA |
| | | 60 | *Silicularia rosea* Meyen, 1834 | 4_1 | FJ550482 | FJ550549 | FJ550406 | MHNG:INVE25072 | New Zealand |
| Lineage 3 (L 3) | Eirenidae | 61 | *Eirene ceylonensis* Browne, 1905 | 23_2 | FJ418647 | FJ418669 | | LMZ-200612-1 | no ID |
| | | 62 | *Eirene menoni* Kramp, 1953 | | FJ418651 | FJ418672 | | LMZ-200706 | no ID |
| | Lovenellidae | 63 | *Eucheilota bakeri** (Torrey, 1904) | 20_1 | AY789831 | AY789774 | | 704CA | USA |
| | | 64 | *Lovenella gracilis* Clarke, 1882 | 12_1 | AY789830 | AY789773 | | 848NJ | USA |
| Infraorder Obeliida *taxon novum* | | | | | | | | | |
| | Campanulariidae | 65 | *Clytia gracilis* (Sars, 1850) | 58_6 | DQ068062 | DQ068052 | | USNM:1078723 | Brazil |
| | | 66 | *Clytia hemisphaerica* (Linnaeus, 1767) | | AY789814 | AY789753 | FJ550457 | 826NS | Denmark |
| | | | | | | | | no ID | France |
| | | 67 | *Clytia hemisphaerica* 2** (Linnaeus, 1767) | | AY346365 | | | no ID | no ID |
| | | 68 | *Clytia hummelincki* (Leloup, 1935) | | AY789809 | AY789744 | | 856SA | South Africa |
| | | 69 | *Clytia linearis* (Thorneley, 1900) | | AY789810 | AY789748 | | USNM:1078729 | USA |
| | | 70 | *Clytia noliformis* (McCrady, 1859) | | DQ064792 | EU272611 | EU272554 | AL1405 | Brazil |
| | | | | | | | | no ID | Brazil |
| | | 71 | *Clytia paulensis* (Vanhöffen, 1910) | | AY346361 | AY789746 | | USNM:1106158 | Italy |
| | | 72 | *Gonothyraea inornata*** Nutting, 1901 | 2_2 | AY789822 | AY789761 | | 564WA | USA |
| | | 73 | *Gonothyraea loveni* (Allman, 1859) | | **KT266616** | **KT722398** | **KT757149** | **MZUSP:1845** | USA |
| | | 74 | *Laomedea calceolifera* (Hincks, 1871) | 7_2 | AY789829 | AY789768 | FJ550447 | 721MA | USA |
| | | | | | | | | MHNG:INVE37296 | France |
| | | 75 | *Laomedea flexuosa* Alder, 1857 | | AY789824 | AY789763 | | 864IC | Iceland |
| | | 76 | *Obelia bidentata* Clark, 1875 | 9_4 | AY789815 | AY789754 | FJ550446 | 76NC | USA |
| | | | | | | | | MHNG:INVE37294 | France |
| | | 77 | *Obelia dichotoma* (Linnaeus, 1758) | | KM603472 | **KT722411** | **KT757168** | **MZUSP:1776** | USA |
| | | 78 | *Obelia geniculata* (Linnaeus, 1758) | | **KT266629** | **KT722412** | **KT757167** | **MZUSP:1866** | USA |
| | | 79 | *Obelia longissima* (Pallas, 1766) | | KM603468 | KM603469 | **KT757159** | **MZUSP:1807** | USA |
| | Eirenidae | 80 | *Eirene brevistylus** ** Huang & Xu, 1994 | 23_1 | FJ418646 | | | LMZ-200703 | no ID |

| | | | | | | | | | |
|---|---|---|---|---|---|---|---|---|---|
| Order Macrocolonia | | | | | | | | | |
| Suborder Staurothecida *taxon novum* | | | | | | | | | |
| Staurothecidae *família nova* | | | | | | | | | |
| | Sertulariidae | 81 | *Staurotheca antarctica* Hartlaub, 1904 | 24_4 | FN424142 | **KT722438** | **KT757203** | **lem_S155** | Antarctica |
| | | 82 | *Staurotheca compressa* Briggs, 1938 | | FN424143 | | | no ID | Antarctica |
| | | 83 | *Staurotheca nonscripta Peña Cantero, Svoboda & Vervoort, 1997* | | FN424144 | | | no ID | Antarctica |
| | | 84 | *Staurotheca pachyclada* (Jäderholm, 1904) | | FN424145 | | | no ID | Antarctica |
| Symplectoscyphidae *família nova* | | | | | | | | | |
| | Sertulariidae | 85 | *Antarctoscyphus elongatus* (Jäderholm, 1904) | 10_3 | FN424138 | | | **lem_S154** | Antarctica |
| | | 86 | *Antarctoscyphus grandis* (Blanco, 1977) | | FN424139 | **KT722383** | **KT757185-6** | **lem_46A** | Antarctica |
| | | 87 | *Antarctoscyphus spiralis (Hickson & Gravely, 1907)* | | FN424140 | **KT722384** | **KT757141** | **lem_S151** | Antarctica |
| | | 88 | *Symplectoscyphus curvatus* (Jäderholm, 1917) | 105_6 | FN424146 | **KT722439** | **KT757144** | **lem_S156** | Antarctica |
| | | 89 | *Symplectoscyphus exochus* Blanco, 1982 | | FN424147 | **KT722440** | **KT757172** | **lem_S123** | Antarctica |
| | | 90 | *Symplectoscyphus glacialis* (Jäderholm, 1904) | | FN424148 | **KT722441** | **KT757178** | **lem_S153** | Antarctica |
| | | 91 | *Symplectoscyphus liouvillei* (Billard, 1914) | | FN424149 | | | no ID | Antarctica |
| | | 92 | *Symplectoscyphus plectilis* (Hickson & Gravely, 1907 | | FN424150 | | | no ID | Antarctica |
| | | 93 | *Symplectoscyphus tricuspidatus* (Alder, 1856) | | AY787907 | | | MHNG:INVE29953 | Iceland |
| Suborder Haleciida *sensu novum* | | | | | | | | | |
| Haleciidae *sensu novum* | | | | | | | | | |
| | Haleciidae | 94 | *Halecium beanii* (Johnston, 1838) | 128_13 | FJ550488 | FJ550560 | FJ550417 | MHNG:INVE34009 | South Africa |
| | | 95 | *Halecium delicatulum* Coughtrey, 1876 | | FN424123 | | | no ID | Antarctica |
| | | 96 | *Halecium halecinum* (Linnaeus, 1758) | | **KT266617** | **KT722399** | **KT757152** | **MZUSP:1782** | USA |
| | | 97 | *Halecium incertus* Naumov & Stepanjants, 1962 | | FN424124 | **KT722400** | **KT757191** | **lem_150** | Antarctica |
| | | 98 | *Halecium labrosum* Alder, 1859 | | AY787916 | FJ550550 | FJ550407 | MHNG:INVE29030 | Iceland |
| | | 99 | *Halecium lankesterii*** (Bourne, 1890) | | AM888316 | | | no ID | United Kingdom |
| | | 100 | *Halecium lenticulare* Trebilcock, 1928 | | FJ550469 | FJ550532 | FJ550387 | MHNG:INVE33461 | New Zealand |
| | | 101 | *Halecium mediterraneum* Weismann, 1883 | | FJ550492 | FJ550566 | FJ550423 | MHNG:INVE34437 | France |
| | | 102 | *Halecium mirabile* Schydlowsky, 1902 | | **KT266618** | | | no ID | no ID |
| | | 103 | *Halecium muricatum* (Ellis & Solander, 1786) | | AY787915 | EU272619 | FJ550408 | MHNG:INVE29028 | Iceland |
| | | 104 | *Halecium petrosum* Stechow, 1919 | | AY787893 | | | MHNG:INVE29763 | Spain |
| | | 105 | *Halecium pusillum* Sars, 1856 | | FJ550499 | FJ550580 | FJ550437 | MHNG:INVE36295 | France |
| | | 106 | *Halecium tenellum* Hincks, 1861 | | AM888322 | | | no ID | United Kingdom |
| Lineage 4 (L 4) | Lafoeidae | 107 | *Abietinella operculata* (Jäderholm, 1903) | 1_1 | FN424136 | | | no ID | Antarctica |
| | | 108 | *Cryptolaria pectinata* (Allman, 1888) | 5_1 | AM887994 | | | DBUA 1280.01 | Gulf of Cádiz |
| | | 109 | *Zygophylax biarmata* Billard, 1905 | 47_1 | AM888343 | | | DBUA 919.02 | Gulf of Cádiz |
| Suborder Sertulariida *taxon novum* | | | | | | | | | |
| Sertularellidae *família nova* | | | | | | | | | |
| | Sertulariidae | 110 | *Sertularella africana* Stechow, 1919 | 131_10 | FJ550490 | FJ550563 | FJ550420 | MHNG:INVE34017 | South Africa |
| | | 111 | *Sertularella diaphana* (Allman, 1885) | | **KT266639** | **KT722430** | **KT757131** | **MZUSP:1725** | Brazil |
| | | 112 | *Sertularella ellisii (Deshayes & Milne Edwards, 1836)* | | FJ550478 | FJ550545 | FJ550402 | MHNG:INVE32156 | Spain |
| | | 113 | *Sertularella gayi* (Lamouroux, 1821) | | | FJ550579 | FJ550436 | MHNG:INVE36302 | France |
| | | 114 | *Sertularella mediterranea* Hartlaub, 1901 | | FJ550479 | FJ550546 | FJ550403 | MHNG:INVE32948 | France |
| | | 115 | *Sertularella polyzonias*** (Linnaeus, 1758) | | AM888340 | | | DBUA 1186.01 | Gulf of Cádiz |
| | | 116 | *Sertularella robusta* Coughtrey, 1876 | | AM888339 | | | no ID | Gulf of Cádiz |
| | | 117 | *Sertularella rugosa* (Linnaeus, 1758) | | AY787906 | | | MHNG:INVE29032 | Iceland |
| | | 118 | *Sertularella sanmatiasensis* El Beshbeeshy, 2011 | | FN424141 | **KT722431** | **KT757202** | **lem_S158** | Antarctica |
| | | 119 | *Sertularella tenella* (Alder, 1857) | | | **KT722432** | **KT757179** | **MZUSP:4466** | Brazil |
| | | 120 | *Symplectoscyphus turgidus** (Trask, 1857) | 105_1 | FJ550462 | FJ550524 | FJ550377 | MHNG:INVE29467 | USA |
| Thyroscyphidae | Thyroscyphidae | 121 | *Thyroscyphus ramosus* Allman, 1877 | 10_2 | **KM822774** | **KM822775** | **KM822776** | **MZUSP:1664** | Brazil |
| | | 122 | *Thyroscyphus marginatus*** (Allman, 1877) | | FJ550495 | FJ550573 | FJ550430 | MHNG:INVE35477 | Honduras |
| | | 123 | *Sertularelloides cylindritheca*** (Allman, 1888) | 1_1 | **KT266640** | **KT722433** | | **MZUSP:1677** | Brazil |
| Sertulariidae *sensu novum* | | | | | | | | | |
| | Sertulariidae | 124 | *Abietinaria abietina* (Linnaeus, 1758) | 37_2 | FJ550484 | FJ550554 | FJ550411 | MHNG:INVE29446 | Iceland |

| | | | | | | | | | |
|---|---|---|---|---|---|---|---|---|---|
| | | 125 | *Abietinaria filicula* (Ellis & Solander, 1786) | | FJ550485 | FJ550555 | FJ550412 | MHNG:INVE29947 | Iceland |
| | | 126 | *Amphisbetia minima* (Thompson, 1879) | 29_2 | AY787903 | EU272602 | EU272544 | MHNG:INVE25071 | New Zealand |
| | | 127 | *Amphisbetia operculata* (Linnaeus, 1758) | | FJ550489 | FJ550561 | FJ550418 | MHNG:INVE34014 | South Africa |
| | | 128 | *Diphasia fallax* (Johnston, 1847) | 28_2 | AY787901 | EU305491 | FJ550414 | MHNG:INVE29950 | Iceland |
| | | 129 | *Diphasia rosacea* (Linnaeus, 1758) | | AM888306 | | | DBUA 1172.02 | United Kingdom |
| | | 130 | *Dynamena crisioides* Lamouroux, 1824 | 19_5 | **KT266609** | **KT722391** | **KT757177** | **MZUSP:1679** | Brazil |
| | | 131 | *Dynamena disticha* (Bosc, 1802) | | AY787909 | | | MHNG:INVE29754 | Spain |
| | | 132 | *Dynamena moluccana*** (Pictet, 1893) | | FJ550494 | FJ550572 | FJ550429 | no ID | Thailand |
| | | 133 | *Dynamena obliqua* Lamouroux, 1816 | | **KT266610** | | | no ID | no ID |
| | | 134 | *Dynamena pumila* (Linnaeus, 1758) | | AY787902 | FJ550558 | FJ550415 | MHNG:INVE29026 | Iceland |
| | | 135 | *Hydrallmania falcata* (Linnaeus, 1758) | 3_1 | FJ550487 | FJ550559 | EU305519 | MHNG:INVE29948 | Iceland |
| | | 136 | *Idiellana pristis* (Lamouroux, 1816) | 2_1 | **KT266622** | **KT722404** | **KT757192-3** | **MZUSP:1651** | Brazil |
| | | 137 | *Salacia desmoides* (Torrey, 1902) | 22_1 | FJ550464 | FJ550528 | FJ550381 | no ID | France |
| | | 138 | *Sertularia argentea* Linnaeus, 1758 | 61_8 | FJ550461 | FJ550520 | FJ550373 | no ID | France |
| | | 139 | *Sertularia cupressina* Linnaeus, 1758 | | FJ550475 | FJ550539 | FJ550395 | MHNG:INVE29949 | Iceland |
| | | 140 | *Sertularia distans* (Lamouroux, 1816) | | **KT266641** | **KT722434** | **KT757163** | **MZUSP:1705** | Brazil |
| | | 141 | *Sertularia marginata* (Kirchenpauer, 1864) | | **KT266642** | **KT722435** | **KT757150** | **MZUSP:1656** | Brazil |
| | | 142 | *Sertularia perpusilla* Stechow, 1919 | | AY787894 | | EU305532 | MHNG:INVE29765 | Spain |
| | | 143 | *Sertularia tumida* Allman, 1877 | | **KT266643** | **KT722436** | **KT757154** | **lem_S54** | Brazil |
| | | 144 | *Sertularia turbinata* (Lamouroux, 1816) | | **KT266644** | **KT722437** | **KT757147** | **MZUSP:1704** | Brazil |
| | | 145 | *Sertularia unguiculata* Busk, 1852 | | AY787904 | | | MHNG:INVE29969 | New Zealand |
| | | 146 | *Thuiaria cornigera*** Kudelin, 1914 | 50_2 | | Z92899 | | no ID | no ID |
| | | 147 | *Thuiaria thuja* (Linnaeus, 1758) | | AY787908 | EU305503 | EU305536 | MHNG:INVE29951 | Iceland |
| Suborder Plumupheniida *taxon novum* | | | | | | | | | |
| Lineage 5 (L 5) | Haleciidae | 148 | *Hydrodendron mirabile* (Hincks, 1866) | 25_1 | DQ855933 | FJ550568 | FJ550425 | MHNG:INVE34779 | Spain |
| | | 149 | *Nemalecium lighti* (Hargitt, 1924) | 2_1 | **KT266628** | **KT722410** | **KT757146** | **lem_S17** | Brazil |
| Infraorder Aglaopheniida *taxon novum* | | | | | | | | | |
| Aglaopheniidae | Aglaopheniidae | 150 | *Aglaophenia acacia* Allman, 1883 | 73_14 | FJ550507 | | | MHNG:INVE37535 | Italy |
| | | 151 | *Aglaophenia elongata* Meneghini, 1845 | | FJ550508 | FJ550593 | FJ550450 | MHNG:INVE37539 | Italy |
| | | 152 | *Aglaophenia harpago* Schenck, 1965 | | FJ550506 | FJ550592 | FJ550449 | MHNG:INVE37531 | Italy |
| | | 153 | *Aglaophenia kirchenpaueri* (Heller, 1868) | | AM887983 | | | no ID | United Kingdom |
| | | 154 | *Aglaophenia latecarinata* Allman, 1877 | | **KT266600** | | | **lem_S48** | Brazil |
| | | 155 | *Aglaophenia lophocarpa* Allman, 1877 | | AM887988 | | | no ID | Gulf of Cádiz |
| | | 156 | *Aglaophenia octodonta* Heller, 1868 | | AM887989 | FJ550541 | FJ550397 | MHNG:INVE32875 | France |
| | | 157 | *Aglaophenia parvula* Bale, 1882 | | DQ855914 | | | MHNG:INVE34013 | South Africa |
| | | 158 | *Aglaophenia picardi* Svoboda, 1979 | | AY787891 | | | MHNG:INVE29758 | Spain |
| | | 159 | *Aglaophenia pluma* (Linnaeus, 1758) | | DQ855916 | FJ550542 | FJ550398 | MHNG:INVE38220 | France |
| | | 160 | *Aglaophenia rhynchocarpa* Allman, 1877 | | **KT266601** | **KT722382** | **KT757157** | **MZUSP:1687** | Brazil |
| | | 161 | *Aglaophenia struthionides* (Murray, 1860) | | **KT266602** | | | no ID | no ID |
| | | 162 | *Aglaophenia tubiformis* Marktanner-Turneretscher, 1890 | | DQ855917 | EU272601 | FJ550399 | MHNG:INVE32960 | France |
| | | | | | | | | MHNG:INVE29967 | France |
| | | 163 | *Aglaophenia tubulifera* (Hincks, 1861) | | AM887992 | | | no ID | United Kingdom |
| | | 164 | *Cladocarpus integer* (Sars, 1873) | 52_1 | FJ550512 | FJ550597 | FJ550453 | MHNG:INVE48754 | Norway |
| | | 165 | *Gymnangium gracilicaule* (Jäderholm, 1903) | 38_3 | DQ855934 | FJ550585 | FJ550442 | MHNG:INVE36839 | Madagascar |
| | | 166 | *Gymnangium hians* (Busk, 1852) | | AY787922 | Z86122 | | MHNG:INVE32586 | Thailand |
| | | | | | | | | no ID | no ID |
| | | 167 | *Gymnangium montagui* (Billard, 1912) | | AM888313 | | | no ID | United Kingdom |
| | | 168 | *Lytocarpia phyteuma* (Kirchenpauer, 1876) | 40_3 | AY787921 | | | MHNG:INVE32597 | Thailand |
| | | 169 | *Lytocarpia canepa* (Blanco & Bellusci de Miralles, 1971) | | **KT266645** | **KT722406** | **KT757196-7** | **lem_S139** | Argentina |
| | | 170 | *Lytocarpia* sp. | | FJ550505 | FJ550591 | FJ550448 | MHNG:INVE36828 | Madagascar |
| | | 171 | *Macrorhynchia philippina* Kirchenpauer, 1872 | 25_2 | **KT266625** | **KT722407** | **KT757153** | **MZUSP:1728** | Brazil |
| | | 172 | *Macrorhynchia phoenicea* (Busk, 1852) | | DQ855935 | FJ550584 | FJ550441 | MHNG:INVE36813 | Madagascar |
| | | 173 | *Macrorhynchia phoenicea* 2** (Busk, 1852) | | FJ550500 | FJ550586 | FJ550443 | MHNG:INVE36832 | Madagascar |
| Infraorder Plumulariida *sensu novum* | | | | | | | | | |
| Schizotrichidae | Halopterididae | 174 | *Schizotricha crassa* Peña Cantero & Vervoort, 2004 | 21_5 | FN424125 | **KT722426** | **KT757136** | **lem_S162** | Antarctica |

| | | | | | | | | | |
|---|---|---|---|---|---|---|---|---|---|
| | | 175 | *Schizotricha falcata* Peña Cantero, 1998 | | FN424126 | **KT722427** | **KT757135** | **lem_S157** | Antarctica |
| | | 176 | *Schizotricha nana* Peña Cantero, Svoboda & Vervoort, 1996 | | FN424127 | **KT722428** | **KT757132** | **lem_S127** | Antarctica |
| | | 177 | *Schizotricha trinematotheca* Peña Cantero & Vervoort, 2005 | | FN424128 | | | no ID | Antarctica |
| | | 178 | *Schizotricha turqueti* Billard, 1906 | | FN424129 | **KT722429** | **KT757142** | **lem_S159** | Antarctica |
| Kirchenpaueriidae | Kirchenpaueriidae | 179 | *Kirchenpaueria pinnata* (Linnaeus, 1758) | 14_1 | AY787911 | FJ550578 | FJ550435 | MHNG:INVE29965 | France |
| | | | | | | | | MHNG:INVE36294 | France |
| | | 180 | *Kirchenpaueria pinnata* 2** (Linnaeus, 1758) | | DQ855923 | FJ550581 | FJ550438 | MHNG:INVE36296 | France |
| | | 181 | *Oswaldella grandis* Peña Cantero, Svoboda & Vervoort, 1997 | 26_5 | FN424132 | **KT722417** | **KT757140** | **lem_13A** | Antarctica |
| | | 182 | *Oswaldella incognita* Peña Cantero, Svoboda & Vervoort, 1997 | | FN424133 | | | no ID | Antarctica |
| | | 183 | *Oswaldella laertesi* Peña Cantero, 2007 | | FN424134 | **KT722418** | **KT757137** | **lem_S129** | Antarctica |
| | | 184 | *Oswaldella shetlandica* Stepanjants, 1979 | | **KT266634** | **KT722419** | **KT757134** | **lem_S128** | Antarctica |
| | | 185 | *Oswaldella stepanjantsae* Peña Cantero, Svoboda & Vervoort, 1997 | | FN424135 | **KT722420** | **KT757133** | **lem_S130** | Antarctica |
| | | 186 | *Pycnotheca mirabilis* (Allman, 1883) | 3_1 | FJ550465 | FJ550529 | FJ550382 | MHNG:INVE25847 | New Zealand |
| | | 187 | *Ventromma halecioides*** (Alder, 1859) | 3_1 | AY787895 | FJ550530 | FJ550385 | MHNG:INVE29766 | France |
| | | 188 | *Ventromma halecioides* 2** (Alder, 1859) | | **KT266623** | | **KT757180** | **lem_S106** | Brazil |
| Halopterididae | Halopterididae | 189 | *Antennella ansini* Peña Cantero & García Carrascosa, 2002 | 23_3 | FJ550470 | FJ550533 | FJ550388 | MHNG:INVE32157 | Spain |
| | | 190 | *Antennella kiwiana* Schuchert, 1997 | | DQ855918 | FJ550534 | FJ550389 | MHNG:INVE33623 | New Zealand |
| | | 191 | *Antennella secundaria* (Gmelin, 1791) | | DQ883445 | FJ550575 | FJ550432 | MHNG:INVE32969 | France |
| | | 192 | *Halopteris alternata* (Nutting, 1900) | 35_8 | DQ855939 | | | AL517 | Brazil |
| | | 193 | *Halopteris catharina* (Johnston, 1833) | | DQ855920 | FJ550517 | FJ550370 | no ID | France |
| | | 194 | *Halopteris carinata* Allman, 1877 | | **KT266619** | **KT722401** | **KT757139** | **MZUSP:1681** | Brazil |
| | | 195 | *Halopteris diaphana* (Heller, 1868) | | **KT266620** | **KT722402** | **KT757138** | **MZUSP:1662** | Brazil |
| | | 196 | *Halopteris liechtensternii* (Marktanner-Turneretscher, 1890) | | AY787888 | FJ550526 | FJ550379 | MHNG:INVE30116 | Spain |
| | | | | | | | | MHNG:INVE29751 | Spain |
| | | 197 | *Halopteris minuta* (Trebilcock, 1928) | | AY787912 | EU272620 | EU272567 | MHNG:INVE25073 | New Zealand |
| | | 198 | *Halopteris schucherti* Galea, 2006 | | | FJ550577 | FJ550434 | no ID | Chile |
| | | 199 | *Halopteris tenella* (Verrill, 1874) | | DQ855938 | | | AL575 | Brazil |
| | | 200 | *Monostaechas quadridens* (McCrady, 1859) | 5_1 | **KT266627** | **KT722409** | **KT757171** | **MZUSP:4632** | Brazil |
| | | 201 | *Polyplumaria flabellata* Sars, 1874 | 8_1 | AM888338 | | | no ID | Gulf of Cadiz |
| Plumulariidae | Plumulariidae | 202 | *Dentitheca bidentata* (Jäderholm, 1905) | 4_1 | **KT266608** | **KT722390** | **KT757143** | **MZUSP1731** | Brazil |
| | | 203 | *Nemertesia antennina* (Linnaeus, 1758) | 43_2 | AY787910 | EU305498 | EU305523 | MHNG:INVE29954 | France |
| | | | | | | | | MHNG:INVE34437 | France |
| | | 204 | *Nemertesia ventriculiformis* (Marktanner-Turneretscher, 1890) | | AM888336 | | | no ID | Gulf of Cadiz |
| | | 205 | *Plumularia filicaulis* Kirchenpauer, 1876 | 93_10 | DQ855926 | FJ550565 | FJ550422 | MHNG:INVE34020 | South Africa |
| | | 206 | *Plumularia habereri* Stechow, 1909 | | | FJ550571 | FJ550428 | no ID | Thailand |
| | | 207 | *Plumularia habereri* 2** Stechow, 1909 | | DQ855927 | | | no ID | Thailand |
| | | 208 | *Plumularia hyalina* (Bale, 1882) | | AY787913 | FJ550552 | FJ550409 | MHNG:INVE25333 | New Zealand |
| | | 209 | *Plumularia insignis* Allman, 1883 | | | **KT722422** | **KT757200-1** | **lem_S134** | Argentina |
| | | 210 | *Plumularia lagenifera* Allman, 1885 | | DQ855928 | FJ550527 | FJ550380 | MHNG:INVE25120 | USA |
| | | 211 | *Plumularia margaretta* (Nutting, 1900) | | FJ550483 | FJ550553 | FJ550410 | MHNG:INVE29760 | Spain |
| | | 212 | *Plumularia margaretta* 2 (Nutting, 1900) | | **KT266636** | **KT722423** | **KT757166** | **MZUSP:1734** | Brazil |
| | | 213 | *Plumularia setacea* (Linnaeus, 1758) | | **KT266637** | **KT722424** | **KT757170** | **lem_S144** | Argentina |
| | | 214 | *Plumularia obliqua* (Johnston, 1847) | | DQ855929 | FJ550544 | FJ550401 | no ID | France |
| | | 215 | *Plumularia pulchella* Bale, 1882 | | DQ855930 | FJ550562 | FJ550419 | MHNG:INVE34016 | South Africa |
| | | 216 | *Plumularia spiralis* Billard, 1911 | | AY787920 | FJ550569 | FJ550426 | MHNG:INVE32600 | Thailand |
| Rogue taxa 1 (R 1) | Haleciidae | 217 | *Hydrodendron gardineri** (Jarvis, 1922) | 25_1 | AY787923 | | | MHNG:INVE32618 | Thailand |
| Rogue taxa 2 (R 2) | Campanulinidae | 218 | *Opercularella lacerata* (Johnston, 1847) | 4_1 | **KT266631** | **KT722414** | **KT757198-9** | **MZUSP:1848** | USA |
| Rogue taxa 3 (R 3) | Lafoeidae | 219 | *Billardia subrufa** (Jäderholm, 1904) | 4_1 | **KT266603** | **KT722385** | **KT757130** | **lem_S124** | Antarctica |
| Rogue taxa 3 (R 4) | Hebellidae | 220 | *Scandia gigas* (Pieper, 1884) | 6_1 | AY787919 | | | MHNG:INVE29764 | Spain |

| Taxa / Category | G/Sp G//Sp | nr | Species | S_s | 16S info | 18S info | 28S info | Voucher | Country/Region |
|---|---|---|---|---|---|---|---|---|---|
| Order “Anthoathecata” | | | | | | | | | |
| Suborder Capitata | | | | | | | | | |
| Corynidae | 13/56 3//3 | 221 | *Coryne pintneri* Schneider, 1897 | 19_1 | AJ878717 | GQ424330 | GQ424303 | MHNG:INVE31976 | France |
| | | | | | | | | Sch251 | France |
| | | 222 | *Sarsia lovenii* (M. Sars, 1846) | 11_1 | GQ395329 | GQ424337 | GQ424310 | MHNG:INVE48736 | Norway |
| | | 223 | *Stauridiosarsia ophiogaster*** (Haeckel, 1879) | 9_1 | EU305473 | EU272615 | EU272560 | KUNHM 2803 | no ID |
| Cladocorynidae | 2/7 1//1 | 224 | *Cladocoryne floccosa* Rotch, 1871 | 5_1 | **KT266607** | EU272608 | **KT757173** | **lem_S40** | Brazil |
| | | | | | | | | AL1407 | Brazil |
| Moerisiidae | 3/10 1//1 | 225 | *Moerisia inkermanica* Paltschikowa-Osroumowa, 1925 | 7_1 | **KT266626** | **KT722408** | **KT757161** | **lem_S41** | Brazil |
| Pennariidae | 1/5 1//1 | 226 | *Pennaria disticha* Goldfuss, 1820 | 5_1 | **KT266635** | **KT722421** | **KT757129** | **MZUSP:1670** | Brazil |
| Porpitidae | 2/4 1//1 | 227 | *Porpita porpita* (Linnaeus, 1758) | 2_1 | AY935322 | GQ424319 | EU883551 | no ID | Mexico |
| | | | | | | | | CWD147 | no ID |
| | | | | | | | | no ID | no ID |
| Zancleidae | 3/40 1//1 | 228 | *Zanclea prolifera* Uchida & Sugiura, 1976 | 33_1 | EU305488 | EU272639 | EU272598 | KUNHM:2793 | no ID |
| Suborder Aplanulata | | | | | | | | | |
| Candelabridae | 3/19 1//1 | 229 | *Candelabrum cocksii* (Cocks, 1854) | 14_1 | AY512520 | AY920758 | AY920796 | MHNG:INVE29591 | France |
| | | | | | | | | no ID | France |
| Corymorphidae | 11/77 1//1 | 230 | *Corymorpha pendula* L. Agassiz, 1862 | 44_1 | EU876538 | EU876565 | EU879936 | KUNHM:2962 | USA |
| Hydridae | 1/37 1//1 | 231 | *Hydra circumcincta* Schulze, 1914 | 37_1 | GU722769 | EU876568 | EU879939 | DMAK13c | USA |
| | | | | | | | | no ID | USA |
| Tubulariidae | 7/69 1//2 | 232 | *Ectopleura obypa* Migotto & Marques, 1999 | 33_2 | **KT266612** | **KT722393** | **KT757156** | **MZUSP:0257** | Brazil |
| | | 233 | *Ectopleura larynx* (Ellis & Solander, 1786) | | **KT266611** | **KT722392** | **KT757164** | **MZUSP:1839** | USA |
| Subordem Filifera I | | | | | | | | | |
| Eudendriidae | 2/75 1//3 | 234 | *Eudendrium caraiuru* Marques & Oliveira, 2003 | 73_3 | | **KT722395** | **KT757160** | **lem_S105** | Brazil |
| | | 235 | *Eudendrium carneum* Clarke, 1882 | | **KT266614** | **KT722396** | **KT757158** | **MZUSP:1700** | Brazil |
| | | 236 | *Eudendrium glomeratum* Picard, 1952 | | AM991301 | **KT722397** | **KT757162** | **lem_S58** | Italy |
| | | | | | | | | MHNG:INVE39717 | France |
| Suborder Filifera II | | | | | | | | | |
| Ptilocodiidae | 5/9 1//1 | 237 | *Hydrichthella epigorgia* Stechow, 1909 | 2_1 | EU305478 | EU272622 | EU272569 | KUNHM:2665 | no ID |
| Proboscidactylidae | 1/9 1//1 | 238 | *Proboscidactyla ornata* (McCrady, 1859) | 9_1 | EU305481 | EU272631 | EU272587 | KUNHM:2767 | no ID |
| Magapiidae | 3/5 1//1 | 239 | *Fabienna sphaerica* Schuchert, 1996 | 2_1 | AM183133 | AY920767 | AY920797 | MHNG:INVE33453 | New Zealand |
| Suborder Filifera III | | | | | | | | | |
| Hydractiniidae | 13/111 1//1 | 240 | *Hydractinia* sp. | 73_1 | **KT266621** | **KT722403** | **KT757165** | **lem_S104** | Chile |
| Stylasteridae | 30/260 1//1 | 241 | *Adelopora crassilabrum* Cairns, 1991 | 4_1 | EU645356 | EU272642 | EU272541 | USNM:1027760 | New Caledonia |
| Suborder Filifera IV | | | | | | | | | |
| Bougainvilliidae | 15/102 2//2 | 242 | *Bimeria vestita* Wright, 1859 | 7_1 | **KT266604** | **KT722386** | **KT757175** | **MZUSP:1653** | Brazil |
| | | 243 | *Bougainvillia muscus* (Allman, 1863) | 31_1 | **KT266606** | **KT722388** | **KT757176** | **MZUSP:4104** | Brazil |
| Cytaeididae | 4/17 1//1 | 244 | *Perarella schneideri* (Motz-Kossowska, 1905) | 5_1 | AM411414 | HM357626 | HM357628 | MHNG:INVE49086 | Italy |
| Pandeidae | 21/87 1//1 | 245 | *Leuckartiara octona* (Fleming, 1823) | 19_1 | AM411421 | EU272624 | EU272573 | no ID | France |
| | | | | | | | | Sch354 | France |
| Oceaniidae | 7/27 1//1 | 246 | *Turritopsis nutricula* McCrady, 1857 | 9_1 | **KT266646** | **KT722442** | **KT757169** | **MZUSP:4093** | Brazil |
| Rathkeidae | 5/14 1//1 | 247 | *Lizzia blondina* Forbes, 1848 | 5_1 | AM411417 | EU272625 | EU272574 | Sch493 | France |
| | | | | | | | | MHNG:INVE48742 | Norway |
| **Taxa / Category** | **G/Sp G//Sp** | **nr** | **Species** | **S_s** | **16S info** | **18S info** | **28S info** | **Voucher** | **Country/Region** |
| Order Siphonophorae | | | | | | | | | |
| Suborder Calycophorae | | | | | | | | | |
| Prayidae | 13/27 1//1 | 248 | *Praya dubia* (Quoy & Gaimard, 1833) | 2_1 | AY935285 | AY937326 | EU305526 | YPM:35346 | USA |
| Suborder Physonectae | | | | | | | | | |
| Apolemiidae | 1/5 1//1 | 249 | *Apolemia* sp. CWD-2005 | 5_1 | AY935290 | AY937331 | EU272546 | YPM:35090 | W Pacific |
| Physophoridae | 1/2 1//1 | 250 | *Physophora hydrostatica* Forsskål, 1775 | 2_1 | AY935300 | AY937342 | EU272582 | YPM:35046 | NW Atlantic |
| Agalmatidae | 11/23 1//1 | 252 | *Cordagalma ordinata*** (Haeckel, 1888) | 2_1 | AY935275 | AY937317 | EU272555 | YPM:35032 | NW Atlantic |
| Forskaliidae | 1/6 1//1 | 253 | *Forskalia edwardsii*** Kölliker, 1853 | 6_1 | AY935278 | AY937320 | EU305516 | YPM:35036 | NW Atlantic |

| | | | | | | | | | |
|---|---|---|---|---|---|---|---|---|---|
| Rhodaliidae | 9/14 1//1 | 254 | *Stephalia dilata* (Bigelow, 1911) | 3_1 | AY935315 | AY937357 | EU305534 | YPM:35358 | Mexico |
| Suborder Cystonectae | | | | | | | | | |
| Physaliidae | 1/1 1//1 | 251 | *Physalia physalis* (Linnaeus, 1758) | 1_1 | AY935284 | AF358065 | EU448095 | YPM:35345 | USA |
| | | | | | | | | no ID | no ID |

| Taxa / Category | G/Sp G//Sp | nr | Species | SP_sp | 16S info | 18S info | 28S info | Voucher | Country/Region |
|---|---|---|---|---|---|---|---|---|---|
| Subclass Trachylinae | | | | | | | | | |
| Order Narcomedusae | | | | | | | | | |
| Aeginidae | 8/8 1//1 | 255 | *Solmundella bitentaculata* (Quoy & Gaimard, 1833) | 1_1 | EU293998 | EU247812 | EU247795 | MHNG:INVE31746 | no ID |
| | | | | | | | | USNM:1107456 | Antarctica |
| Order Trachymedusae | | | | | | | | | |
| Geryoniidae | 4/4 2//2 | 256 | *Geryonia proboscidalis* (Forsskål, 1775) | 1_1 | EU293979 | EU247816 | EU247807 | JAM RB-BWD-4 | Japan |
| | | 257 | *Liriope tetraphylla* (Chamisso & Eysenhardt, 1821) | 1_1 | **KT266624** | **KT722405** | **KT757194-5** | **lem_S10** | Brazil |
| Rhopalonematidae | 15/36 3//3 | 258 | *Aglantha digitale* (O. F. Müller, 1776) | 4_1 | EU293985 | EU247821 | AY920791 | USNM:1073329 | USA |
| | | | | | | | | no ID | no ID |
| | | 259 | *Crossota rufobrunnea* (Kramp, 1913) | 5_1 | EU293986 | EU247824 | EU247800 | JAM I060319b-N4 | Japan |
| | | 260 | *Pantachogon haeckeli* Maas, 1893 | 3_1 | EU293988 | AF358062 | AY920792 | MBARI T981 SS1 | no ID |
| | | | | | | | | no ID | no ID |
| Halicreatidae | 6/9 1//1 | 261 | *Botrynema brucei* Browne, 1908 | 2_1 | EU293982 | EU247822 | EU247798 | no ID | Japan |
| Order Limnomedusae | | | | | | | | | |
| Olindiidae | 17/45 2//2 | 262 | *Maeotias marginata* (Modeer, 1791) | 1_1 | AY512508 | AF358056 | EU247810 | no ID | USA |
| | | 263 | *Olindias sambaquiensis* Müller, 1861 | 6_1 | **KT266630** | **KT722413** | **KT757174** | **lem_S01** | Brazil |

| Taxa / Category | Nr | 16S info | 18S info | 28S info |
|---|---|---|---|---|
| **TOTAL Leptothecata** | **220** | 211 | 165 | 139 |
| **TOTAL “Anthoathecata”** | 27 | 27 | 27 | 27 |
| **TOTAL Siphonophorae** | 7 | 7 | 7 | 7 |
| **TOTAL Trachylina** | 9 | 9 | 9 | 9 |
| **TOTAL HYDROZOA** | **263** | **254** | **208** | **182** |

Supplementary Table 2 – Information about the primers used to generate molecular markers using PCR. In bold we indicate the primers developed by us in the Marine Evolution Laboratory (*Laboratório de Evolução Marinha* – LEM-IB-USP). Primers developed by the Cnidaria Tree of Life are indicated by CNIDToL, GB indicates GenBank for the reference sequences used to map the relative position of the primer pairing with the DNA regions of interest in the PCR reactions.

| **primers Nuclear Markers** | | | | | |
|---|---|---|---|---|---|
| **Gene: 28S** | **Sequence primer 5´-3´** | **f/r** | **tm – base pair** | **Reference** | **Map of *primers* (GB: GU722663)** |
| **Name of primer** | | | | | ***Hydra circumcincta* (Hydrozoa:Medusozoa)** |
| F63 mod | ACCCGCTGAAYTTAAGCATATHANTMAG | f | 55°C – 28bp | Medina et al., 2001 | ITS-2 --- 28S start |
| **F15** | **CTAACAAGGATTCCCCTAGTAACGGCGAGT** | **f** | **62°C – 30bp** | **LEM lab** | **15-44** |
| F97 | CCYYAGTAACGGCGAGT | f | 50ºC - 17bp | CNIDToL lab | 28-44 |
| F798 | CCGTCTTGAAACACGGACC | f | 56°C - 19bp | Medina et al., 2001 | 785-803 |
| R798 | GGTCCGTGTTTCAAGACGG | r | 56°C - 19bp | Medina et al., 2001 | 785-803 |
| 28SRD4.8A-F | ACCTATTCTCAAACTTTAAATGG | f | 46°C - 26bp | Schwendinger & Giribet. 2005 | 1,207-1,229 |
| F1414 | GACAGCAGGACGGTGGYCATGG | f | 59°C - 22bp | Medina et al., 2001 | 1,391-1,412 |
| R1446 | GTTGTTACACACTCCTTAGCGG | r | 55°C - 22bp | Medina et al., 2001 | 1,444-1,423 |
| F1586 | GTGCAGATCTTGGTDGNAGTAGCAAATATTC | f | 60ºC - 31bp | Medina et al., 2001 | 1,598-1,628 |
| F1689 | CTAAGMSRYAGGGAAAYTC | f | 52°C - 19bp | CNIDToL lab | 1,701-1,719 |
| **F2103** | **GATCCGTAACTTCGGGAAAAGGATTGGCTC** | **f** | **62°C - 30bp** | **LEM lab** | **2,103-2,132** |
| F2076sq | TAACYTCGGGAWAAGGATTGGCTC | f | 55ºC - 24bp | Medina et al., 2001 | 2,109-2,132 |
| **R2144** | **GAGCCAATCCTTTTCCCGAAGTT** | **r** | **57°C - 23bp** | **LEM lab** | **2,110-2,132** |
| R2084 | AGAGCCAATCCTTTTCC | r | 45ºC - 17bp | CNIDToL lab | 2,130-2,146 |
| R2813 | CAGRTGTRCCGCCCCAGCCAAACT | r | 66°C - 24bp | Medina et al., 2001 | 2,813-2,836 |
| F2813 | AGTTTGGCTGGGGCGGYACA | f | 65°C - 20bp | Medina et al., 2001 | 2,813-2,836 |
| F2800 | GCAGGTGTCCTAAGGYRAGCTC | f | 59°C - 22bp | Voigt et al., 2004 | 2,849-2,870 |
| **R3214** | **GTGAATTCTGCTTCACAATGATAGGAAGAGCC** | **r** | **60°C - 32bp** | **LEM lab** | **3,081-3,112** |
| R3264 | TTCYGACTTAGAGGCGTTCAG | r | 51°C - 21bp | Medina et al., 2001 | 3,260-3,281 |
| **Gene: 18S** | **Sequence primer 5´-3´** | **f/r** | **tm – base pair** | **Reference** | **Map of primers (GB: Z86108)** |

| Name of primer | | | | | *Obelia* sp. (Hydrozoa:Medusozoa) |
|---|---|---|---|---|---|
| A | AACCTGGTTGATCCTGCCAGT | f | 54°C - 21bp | Medlin et al., 1988 | 1-21 |
| 18Sini | ATCCTGCCAGTAGTCATA | f | 45°C - 18bp | Turbeville et al., 1991 | 11-28 |
| 554f (GBF) | AAGTCTGGTGCCAGCAGCCGC | f | 62°C - 21bp | Turbeville et al., 1991 | 568-588 |
| 18S-C | CGGTAATTCCAGCTCCAATAG | f | 50°C - 21bp | Apakupakul et al., 1999 | 588-608 |
| 18S-L | CCAACTACGAGCTTTTTAACTG | r | 50°C - 22bp | Apakupakul et al., 1999 | 627-648 |
| **18S inter (Rv)** | **GCGAAGAAGAACAGACCGATCAG** | **r** | **52°C - 23bp** | **LEM lab** | **689-711** |
| 18S-O | AAGGGCACCACCAGGAGTGGAG | f | 60°C - 22bp | Apakupakul et al., 1999 | 1,166-1,187 |
| 18J-5 | GCCTGCGGCTTAATTTGACTCAACACGGG | f | 63°C - 29bp | Hillis & Dixon. 1991 | 1,187-1,215 |
| 1282r (GBR) | TCACTCCACCAACTAAGAACGGC | r | 56°C - 23bp | Turbeville et al., 1991 | 1,293-1,315 |
| 18S-Y | CAGACAAATCGCTCCACCAAC | r | 54°C - 21bp | Apakupakul et al., 1999 | 1,303-1,323 |
| Rh-18S9R | GATCCTTCCGCAGGTTCACCTAC | r | 56°C - 23bp | Turbeville et al., 1991 | 1,811-1,833 |
| B | TGATCCTTCCGCAGGTTCACCT | r | 57°C - 22bp | Medlin et al., 1988 | 1,814-1,835 |
| **primers Mitochondrial Markers** | | | | | |
| **Gene: 16S** | **Sequence primer 5´-3´** | **f/r** | **tm – base pair** | **Reference** | **Map of primers (GB: DQ787873)** |
| **Name of primer** | | | | | ***Aurelia aurita*** **(Scyphozoa:Medusozoa)** |
| CB1 | TCGACTGTTTACCAAAAACATA | f | 60°C - 32bp | Cunningham & Buss. 1993 | 1,028-1,049 |
| 16Sar | CGCCTGTTTATCAAAAACAT | f | 49°C - 20bp | Simon et al., 1991 | 1,029-1,048 |
| SHB | GACTGTTTACCAAAAACATA | f | 44°C - 20bp | Schroth et al 2002 | 1,030-1,049 |
| **1 Hyd-Scyph** | **TGACCGTGDTAADGTAGC** | **f** | **50°C -18bp** | **LEM lab** | **1,134-1,151** |
| **2 Scyhom** | **CTGTTATCCCTACGGTAAC** | **r** | **49°C - 19bp** | **LEM lab** | **1,519-1,537** |
| **2 Hydrom** | **CTGTTATCCCTAAGGTAGC** | **r** | **49°C - 19bp** | **LEM lab** | **1,519-1,537** |
| **16S-R-BR** | **CATAATTCAACATCGAGG** | **r** | **45°C - 18bp** | **LEM lab** | **1,581-1,598** |
| CB2 | ACGGAATGAACTCAAATCATGTAAG | r | 52°C - 25bp | Cunningham & Buss. 1993 | 1,652-1,676 |

82

Supplementary Table 3 - Protocols to generate nucleotide sequences for this study along with the optimized volumes for the PCR and BigDye® reactions. For specific time (Tm) for every primer for PCR, Supplementary Table 3.

| **A – DNA extraction** | **Protocol summary** | |
|---|---|---|
| Chaotropic resin Chelex INSTAGENE® (BIO-RAD #732-6030) | 1. Incubate tissue sample [*Tm* = 56ºC / T = 30´] + [Tm = 99ºC / T = 8´] | |
| | 2. Extraction quality control via NANODROP® | |
| | 3. Store at -4ºC | |
| **B - Amplification by PCR** | **Protocol summary** | |
| Basic program of the thermocycler for the PCR (expected fragment length ~600pb) | 1. Initial cycle: *Tm* = 94ºC / T = 5´ | |
| | 2. Cycle repetition (35 or 40 Cycles): [*Tm* = 94ºC / T = 50´´]+[*Tm* = primer / T = 50´´]+[*Tm* = 72ºC / T = 50´´] | |
| | 3. Final cycle: *Tm* = 72ºC / T = 7´ | |
| | 4. PCR quality control via NANODROP® | |
| | 5. Store at -4ºC | |
| | Reagents and concentrations of PCR mixture | Volume |
| | Ultra pure $H_2O$ | 14.5ul |
| | Buffer *Taq*: 10X | 2.5ul |
| | dNTPs (combined): 100 mM | 1.33ul |
| | $MgCl_2$: 3.5 mM | 3.5ul |
| | *Primers* (*forward* and *reverse*: 0.6 mM each) | 1.5ul |
| | *Taq* polymerase: 0.8 units (5U/ml) | 0.17ul |
| | DNA ( ~30 ng) | 1.5ul |
| | **TOTAL volume for PCR reaction** | **25ul** |
| **C – Double-stranded DNA purification (PCR product)** | **Protocol summary** | |
| Magnetic particles AMPure XP® (Agencourt #A63880) | 1. Add 1.8x of the volume of the PCR of AMPure XP solution. Remove supernatant in the magnetic bed | |
| | 2. Two ethanol 70% washes | |
| | 3. Final dilution in 15ul ulta pure $H_2O$ | |
| | 4. PCR prufication control: gel agarose 1.25%. stain with Gel-Red® | |
| | 5. Store at -4ºC | |
| **D – BigDye® Reaction** | **Protocol summary** | |
| Thermocycler Program for the ABI BigDye® (Life Technologies #4336935). | 1. Initial cycle: *Tm* = 96ºC / T = 1´ | |
| | 2. Cycle repetition (40 Cycles): [*Tm* = 96ºC / T = 10´´]+[*Tm* = *primer* / T = 5´´]+[*Tm* = 60ºC / T = 4´] | |
| | 3. Store at -4ºC | |
| | Reagentes and concentrations for the BigDye® reaction. | Volume |

| | | |
|---|---|---|
| | Buffer BigDye® Terminator v3.1 | 2ul |
| | BigDye® Terminator v3.1 | 1ul |
| | *Primer* (F *or* R*)* | 0.5ul |
| | PCR purification (final DNA concentration: 50-70ng per reaction. *e.g.*: 50ng/ul) | 1ul |
| | ulta pure H2O | 5.5ul |
| | **TOTAL reaction volume BigDye®** | **10ul** |
| **E – Analysis of the obtained sequences from the Sequencer ABI® 3730 DNA Analyser** | **Software** | |
| Analysis of the .abi. files and generation of contigs (final format: genbank .gb) | Geneious® 5.1.6 | |

## Rogue taxa analysis

To evaluate potential phylogenetic artifacts from unstable taxa, full rogue taxa analyses were perfomed in combined datasets. Original and filtered data matrices, together with optimal tree and (non parametric) bootraped trees were submitted to the online version of RogueNaRok server (http://rnr.h-its.org) using default parameters. We pruned the top-ten rogue taxa for every data matrix (Supplementary Table S4). Pruned datasets were analyzed and like complete datamatrices (Figs. S13-S15). Major clades represented in main results are also presented in these analyses with high support (Table S5). So, we concluded that i) those Leptothecata unstable terminals identified with RogueNaRok would be treated as *incertae sedis* in main results, ii) they do not affect relationships between major clades in most of the cases, and iii) stable clades with high support were used to propose a new phylogenetic classification of Leptothecata.

## Bibliography


1. Collins, A. G. Towards understanding the phylogenetic history of Hydrozoa: Hypothesis testing with 18S gene sequence data. *Sci. Mar.* **64,** 5–22 (2000).
2. Schuchert, P. Hydrozoa. Accessed through: World Register of Marine Species at http://www.marinespecies.org/.

Supplementary Table S4 – Top-ten rogue taxa identified by RogueNaRok. Species in bold face and grey background cells were rogues in all concatenated matrices, species in bold face are rogues in two out of the three datamatrices.

| | RogueNaRok (Mayoriry Rule MR) | | |
|---|---|---|---|
| | 16S18S28S_N | 16S18S28S | 16S18S28S_Nrw4 |
| 1 | ***Hydrodendron gardineri*** | ***Hydrodendron gardineri*** | ***Hydrodendron gardineri*** |
| 2 | ***Scandia gigas*** | ***Scandia gigas*** | ***Aequorea conica*** |
| 3 | ***Aequorea conica*** | ***Aequorea conica*** | ***Scandia gigas*** |
| 4 | ***Symplectoscyphus turgidus*** | ***Symplectoscyphus turgidus*** | ***Symplectoscyphus tricuspidatus*** |
| 5 | *Halecium delicatulum* | ***Schizotricha trinematotheca*** | ***Schizotricha trinematotheca*** |
| 6 | ***Turritopsis nutricula*** | *Physalia physalis* | *Aglaophenia latecarinata* |
| 7 | *Cordagalma ordinata* | ***Sertularia unguiculata*** | *Dynamena obliqua* |
| 8 | ***Sertularia unguiculata*** | *Turritopsis nutricula* | *Dynamena disticha* |
| 9 | ***Schizotricha trinematotheca*** | ***Billardia subrufa*** | *Physophora hydrostatica* |
| 10 | ***Billardia subrufa*** | *Symplectoscyphus tricuspidatus* | *Praya dubia* |

Supplementary Table S5 - Results of nodal support for the main groups of Leptothecata pruned-of-rogues and complete matrices. Black cells indicate nodes with significant support according to all or most support methods (3 of 4 support methods for multilocus, and 3 of 3 for single locus analyses); grey cells with significant supports for one or two methods (ab = aBAYES; sh = SH_aLRT, bs = Bootstrap; see Fig. 1 for details); white cells reprent clades with non-significant support in all methods. Asterisk indicates monophyletic taxon absent in the topology but present in a para- or polyphyletic composition (i.e., small patristic distance between terminals); number in parenthesis () indicates number of figure in main text.

| Taxon | 16S18S28S_N | | 16S18S28S | | 16S18S28S_Nrw4 | |
|---|---|---|---|---|---|---|
| | all taxa (1) | rogue taxa | all taxa (2) | rogue taxa | all taxa (3) | rogue taxa |
| **Lafoeida** | | | | | | |
| **Laodiceida** | | ab / bs | | ab / sh | ab | ab |
| **Statocysta** | | | | | | |
| *Campanulinida* | | | | | | |
| *Eirenida* | | | | | | |
| Eirenids I | | | | | | |
| Eirenids II | | | | | | |
| *Proboscoida* | | | | | ab | |
| Campanulariida | | | | | | |
| Obeliida | ab / sh | | | | | |
| **Macrolonia** | | | | | | |
| *Staurothecida* | | no* | no* | no* | no* | no* |
| Staurothecidae | | | | | | |
| Symplectoscyphidae | | | | | | |
| *Haleciida* | | | | | | |
| *Sertulariida* | | | | | | |
| Sertularellidae | | | | | no* | |
| Thyroscyphidae | | | | | | |
| Sertulariidae | | | | | | bs / sh |
| *Plumupheniida* | | | | | | |
| Aglaopheniida | | | | | | |
| Plumulariida | | | ab / bs | | | |
| Schizotrichidae | | | | | | |
| Kirchenpaueriidae | | | | | | |
| Halopterididae | | | | | | |
| Plumulariidae | | | | | | |

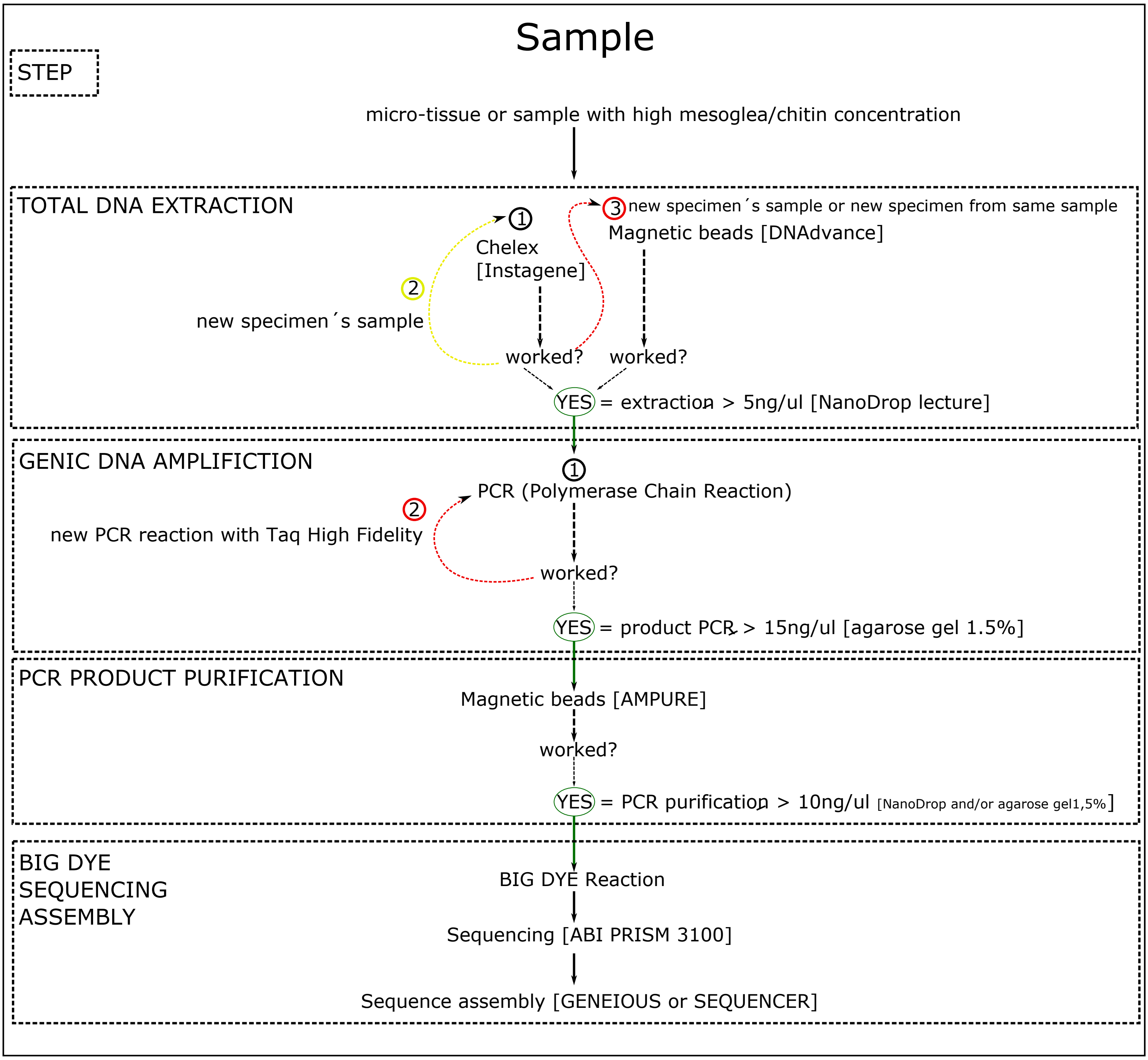
Sample
STEP
micro-tissue or sample with high mesoglea/chitin concentration
TOTAL DNA EXTRACTION
1
Chelex [Instagene]
2
new specimen´s sample
3
new specimen´s sample or new specimen from same sample
Magnetic beads [DNAdvance]
worked?
worked?
YES = extraction > 5ng/ul [NanoDrop lecture]
GENIC DNA AMPLIFICTION
1
PCR (Polymerase Chain Reaction)
2
new PCR reaction with Taq High Fidelity
worked?
YES = product PCR > 15ng/ul [agarose gel 1.5%]
PCR PRODUCT PURIFICATION
Magnetic beads [AMPURE]
worked?
YES = PCR purification > 10ng/ul [NanoDrop and/or agarose gel1,5%]
BIG DYE SEQUENCING ASSEMBLY
BIG DYE Reaction
Sequencing [ABI PRISM 3100]
Sequence assembly [GENEIOUS or SEQUENCER]

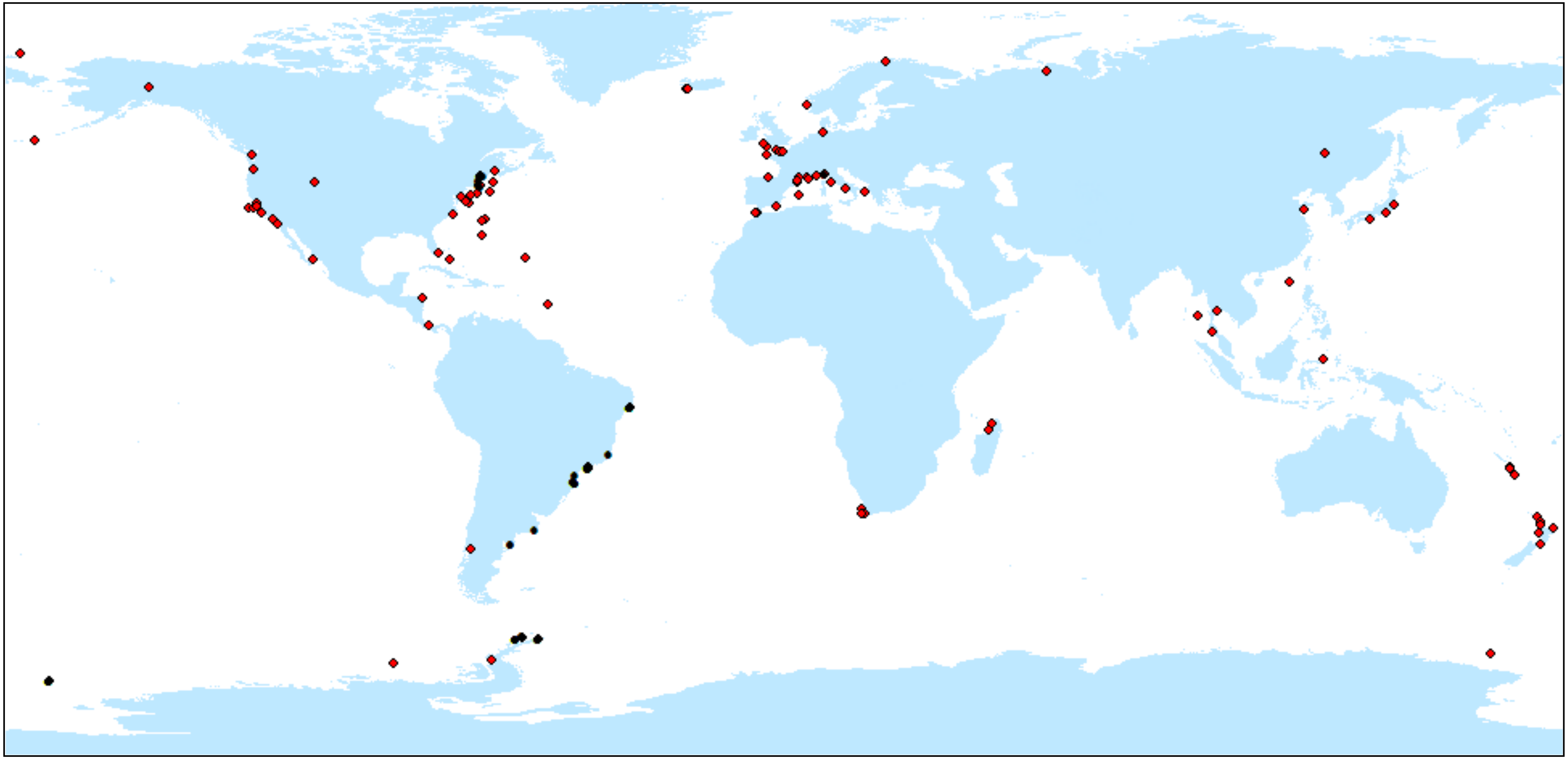

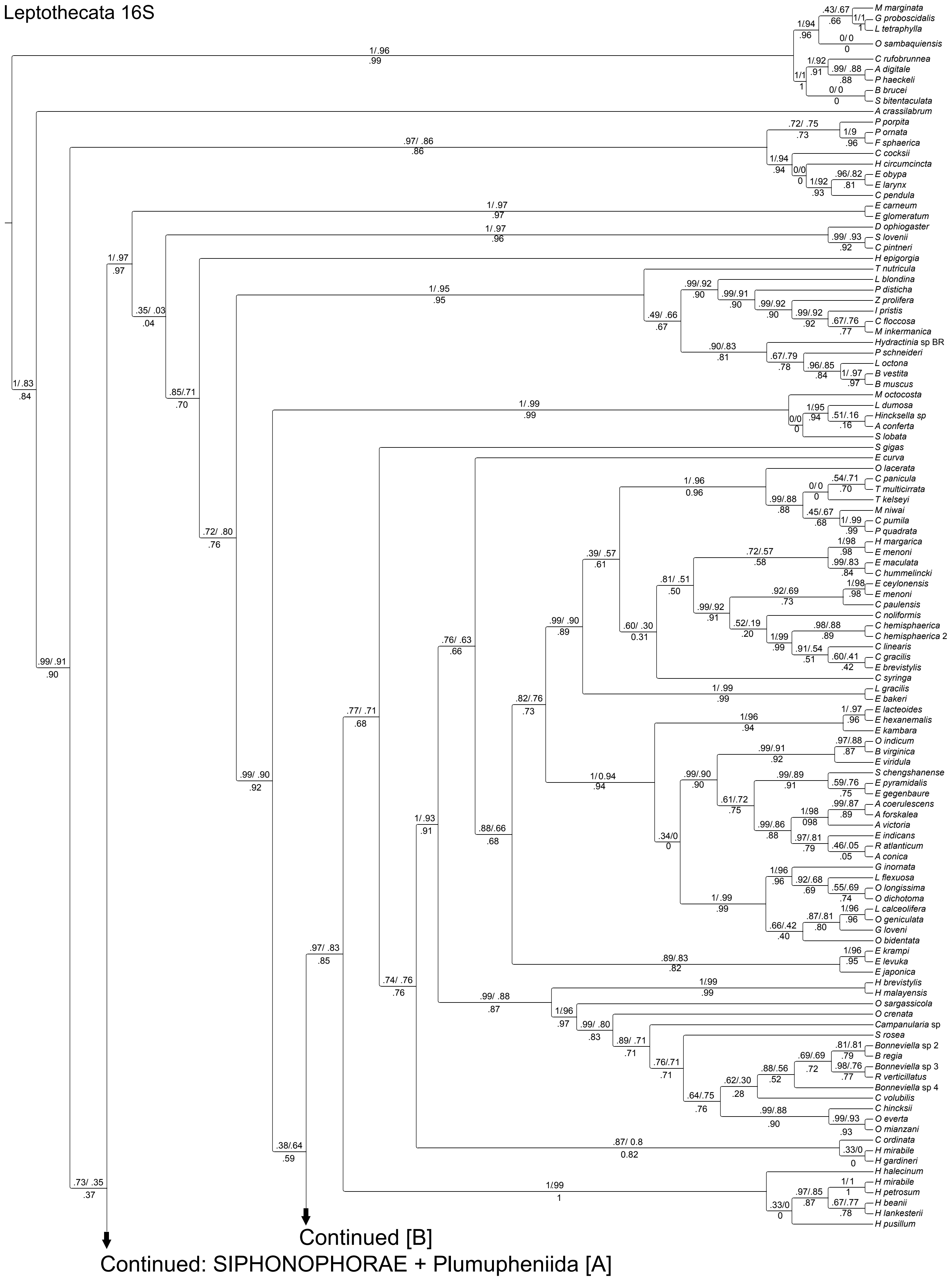


Supplementary Figure S3_A

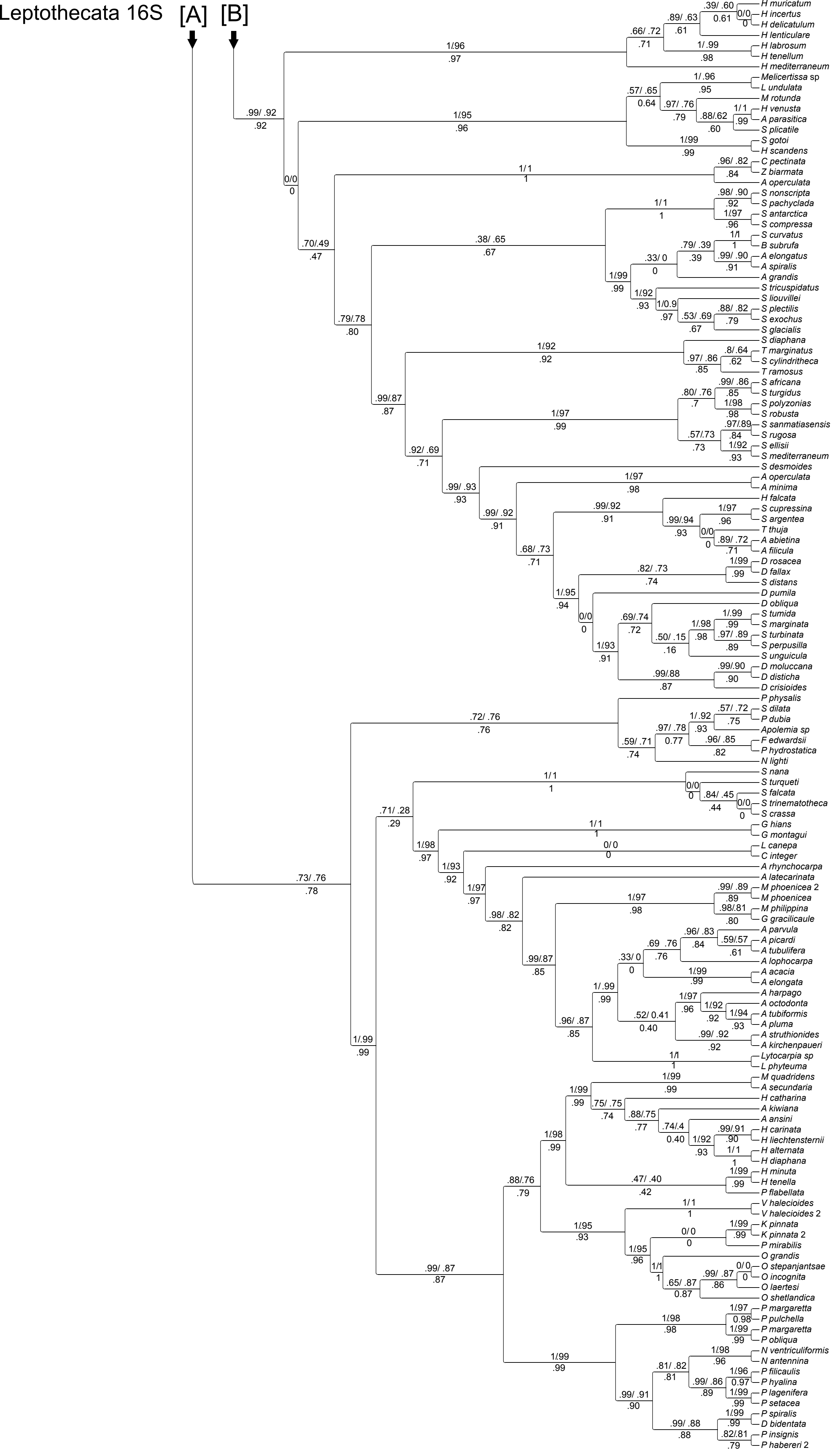


Supplementary Figure S3_B - continued

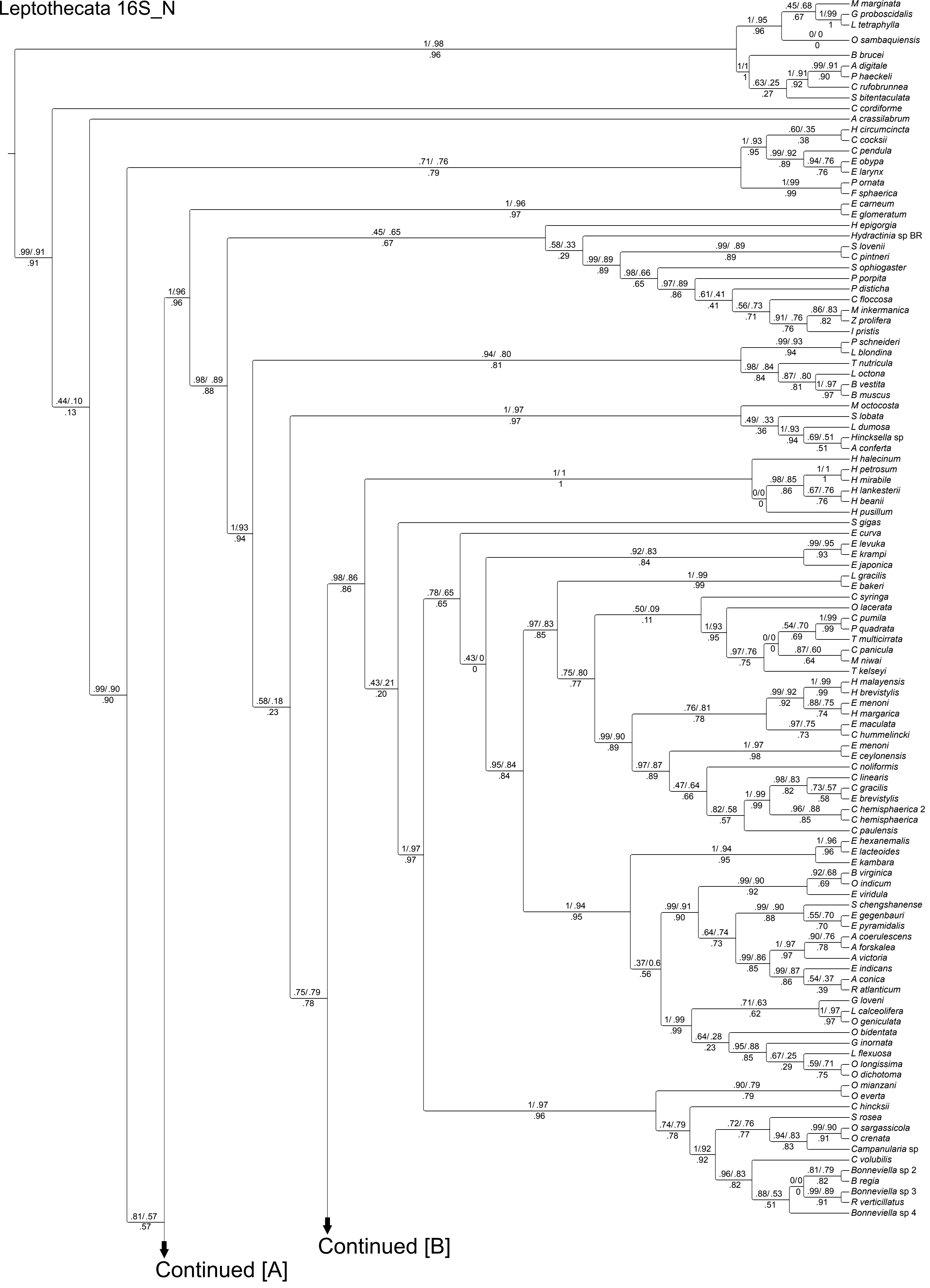


Supplementary Figure S4_A

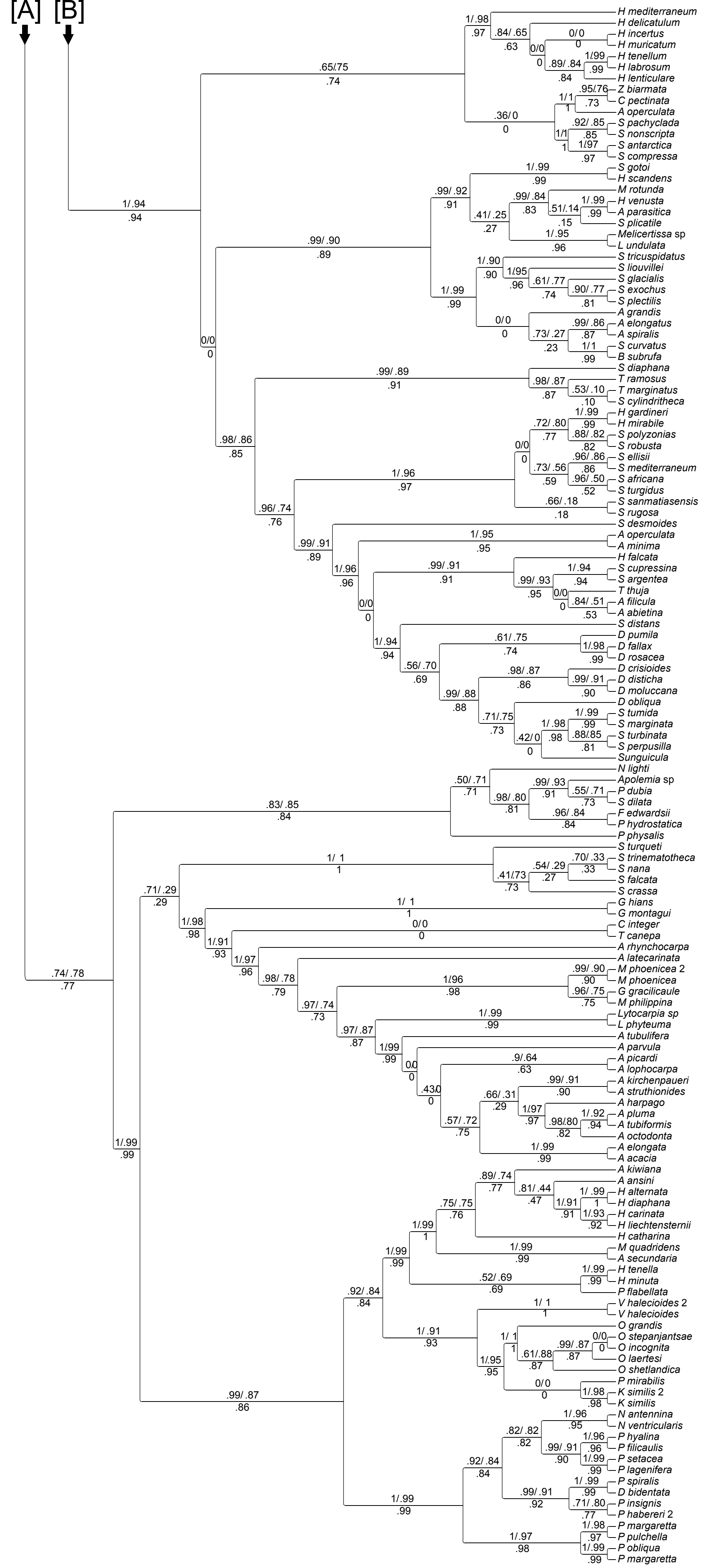


FigurSupplemetary Figure S4_B - continued

Leptothecata 16S_Nrw4

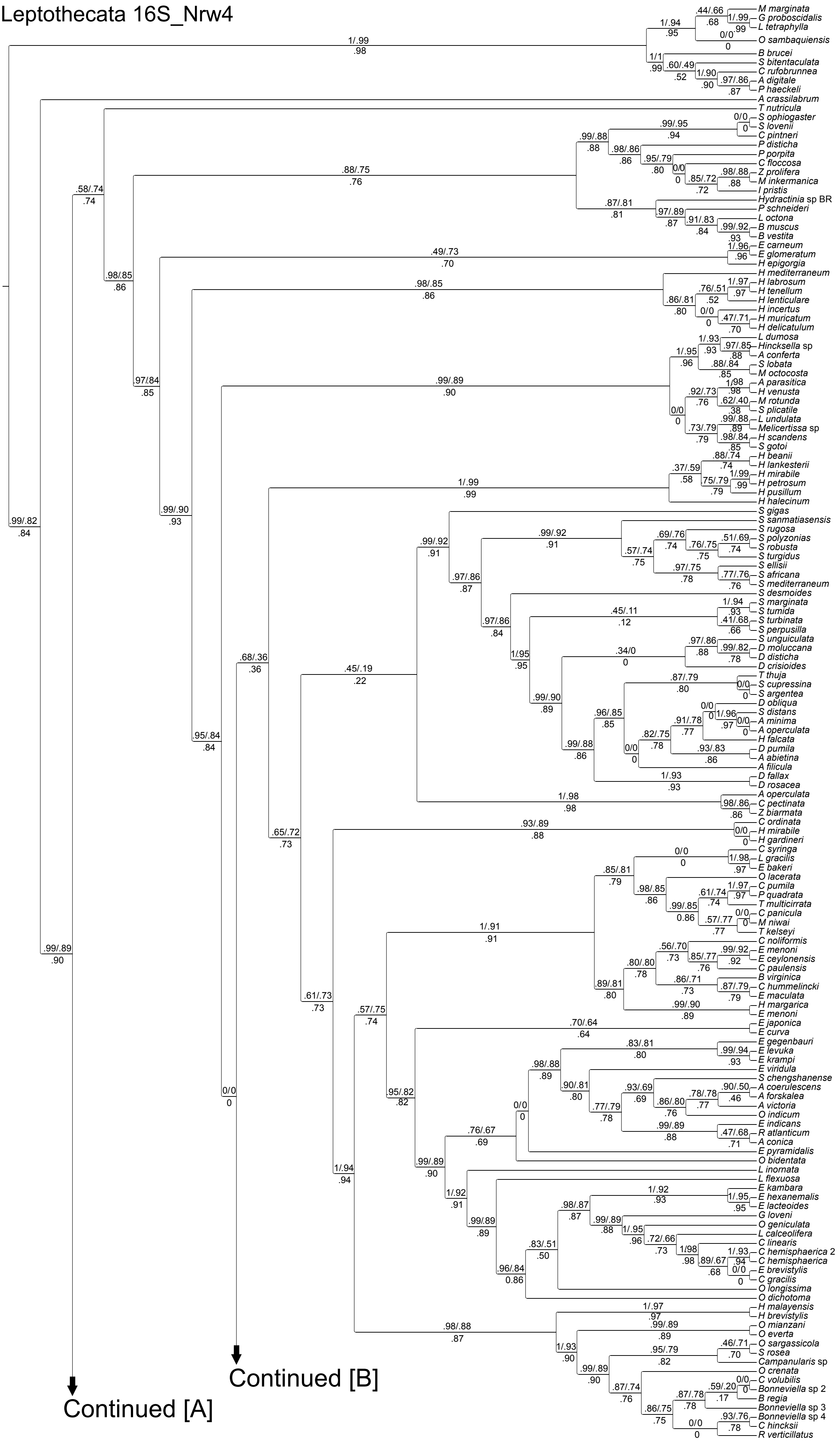

Continued [A]

Continued [B]

Supplementary Figure S5_A

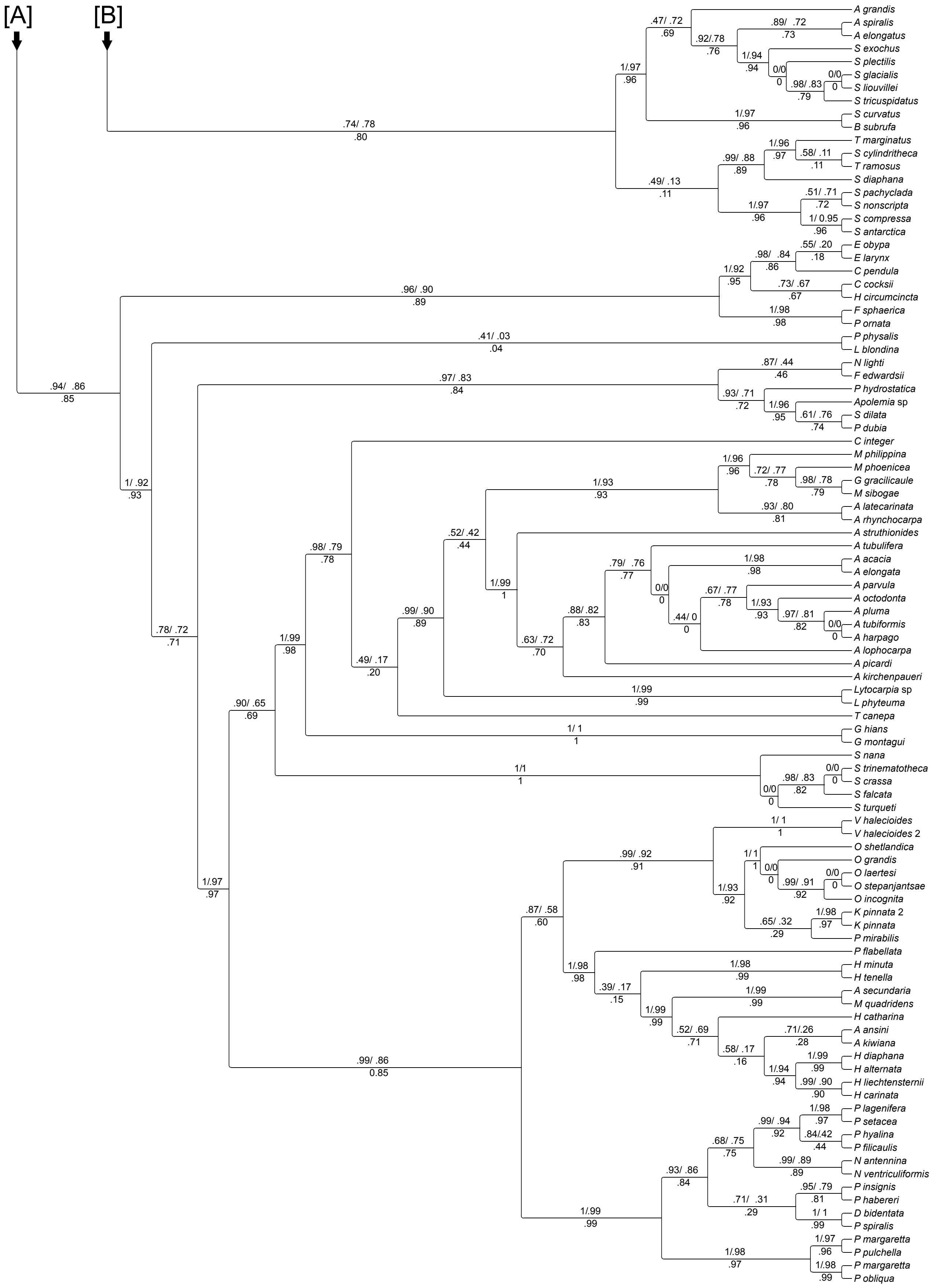


Supplementary Figure S5_B

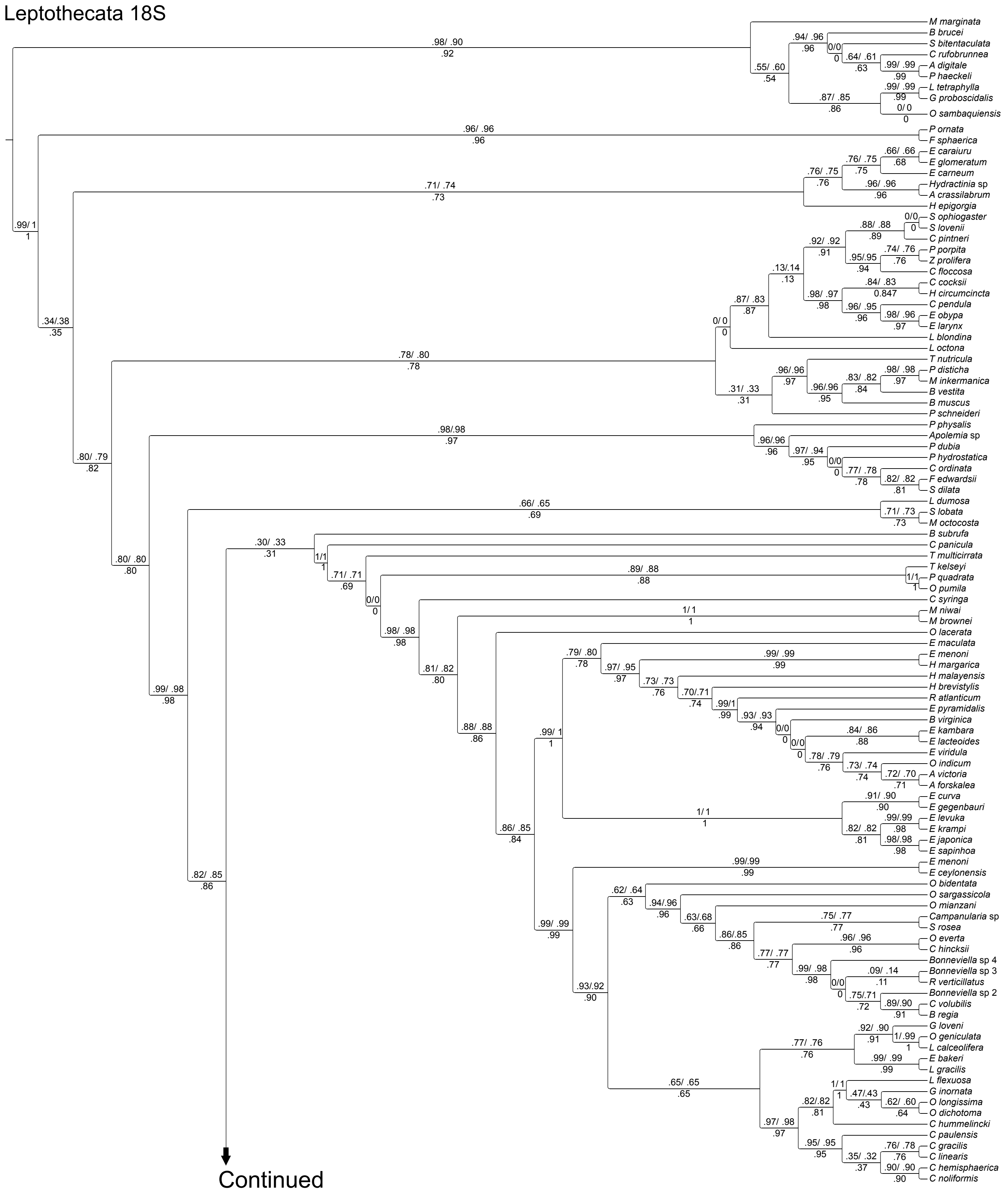


Supplementary Figure S6_A

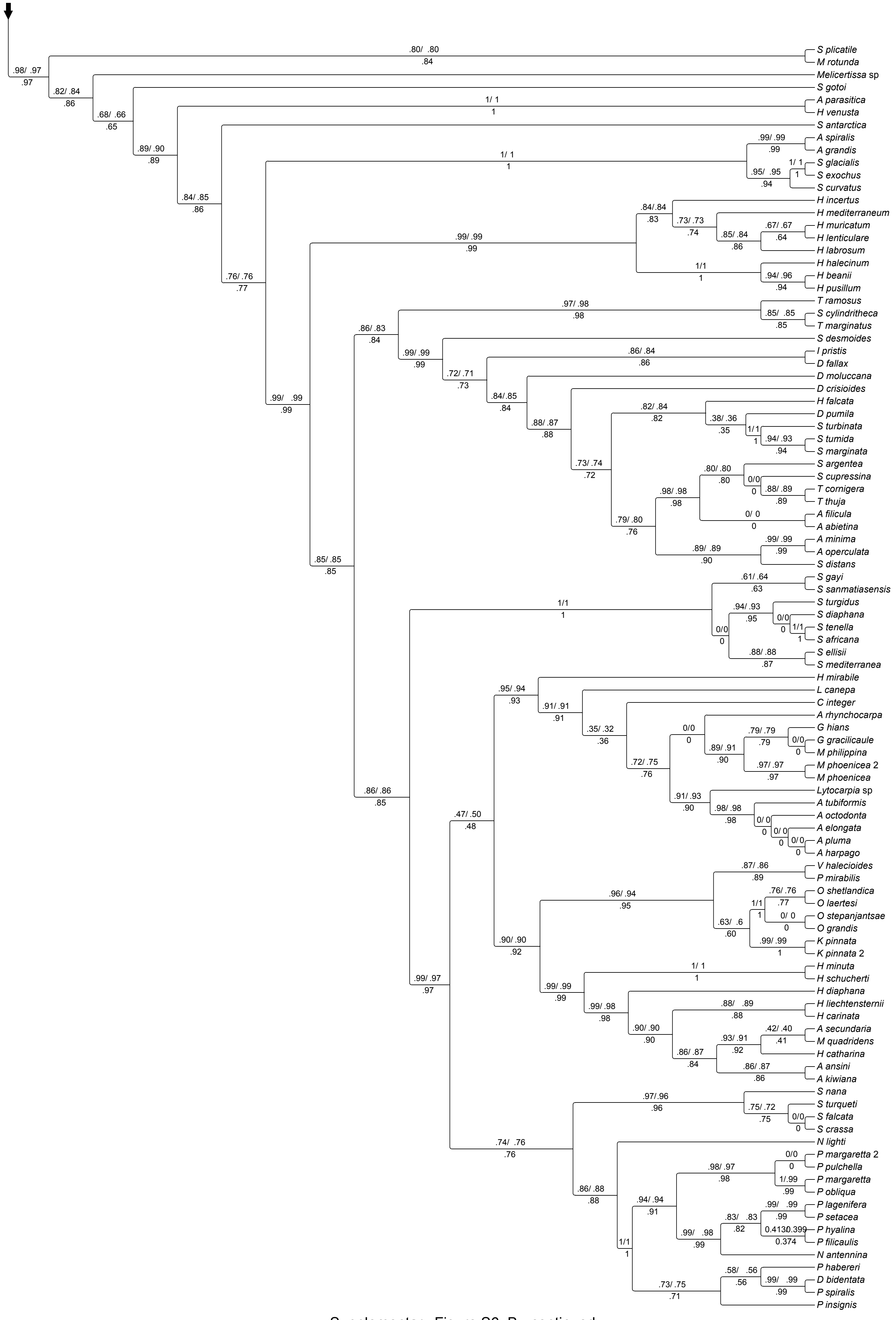


Supplementary Figure S6_B - continued

# Leptothecata 18S_N

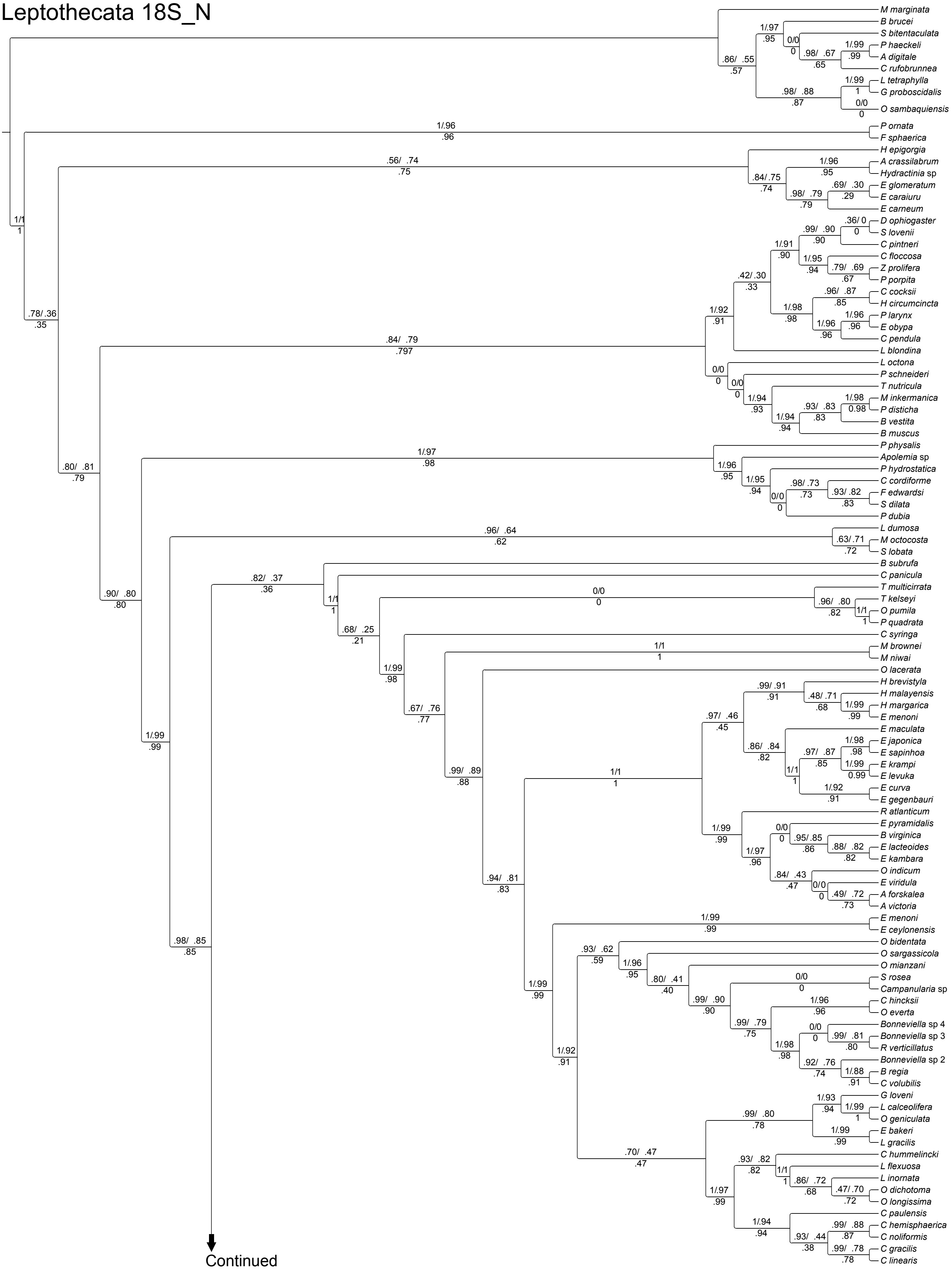


Supplementary Figure S7_A

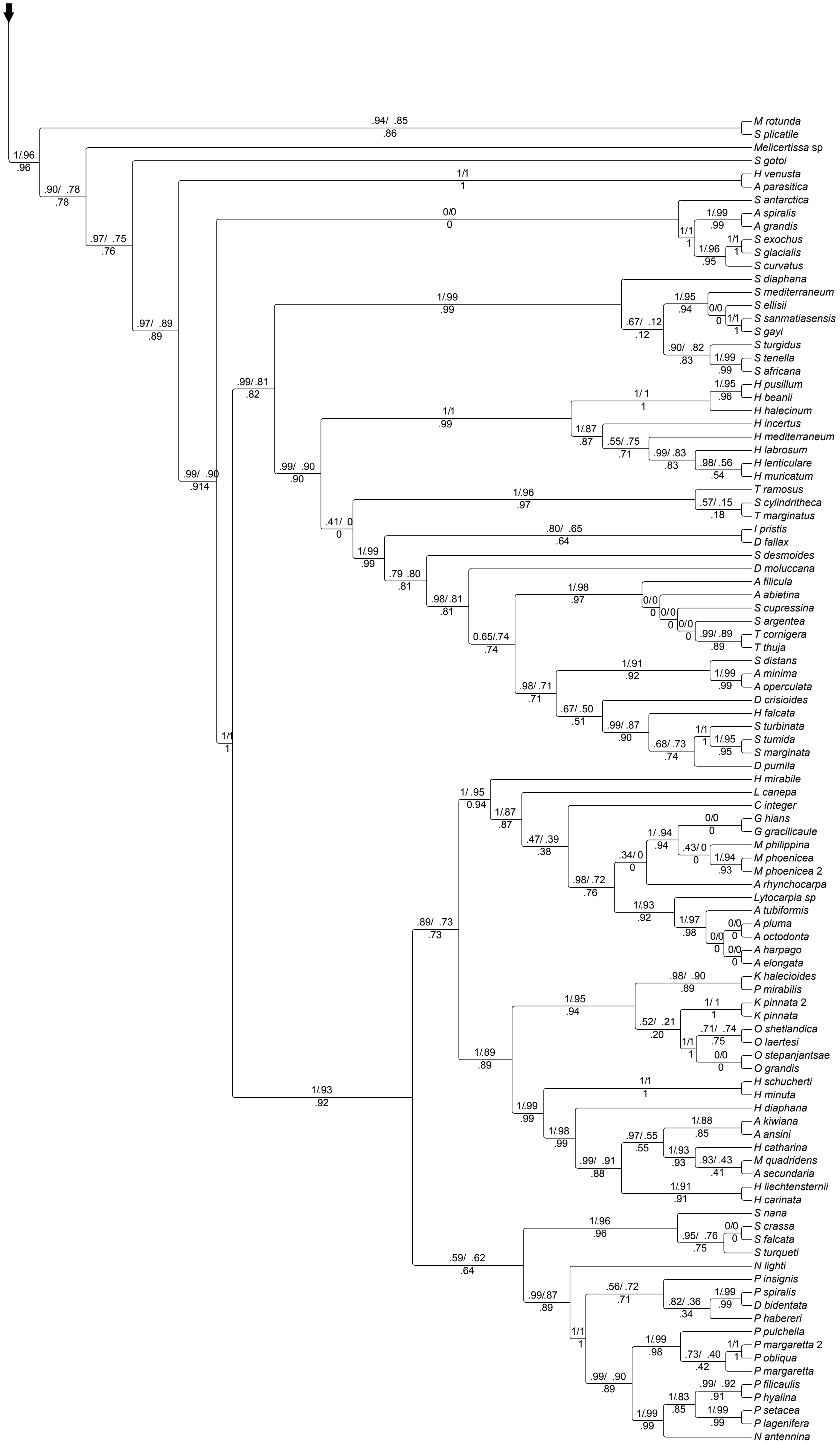

Supplementary Figure S7_B - continued

Leptothecata 18S_Nrw4

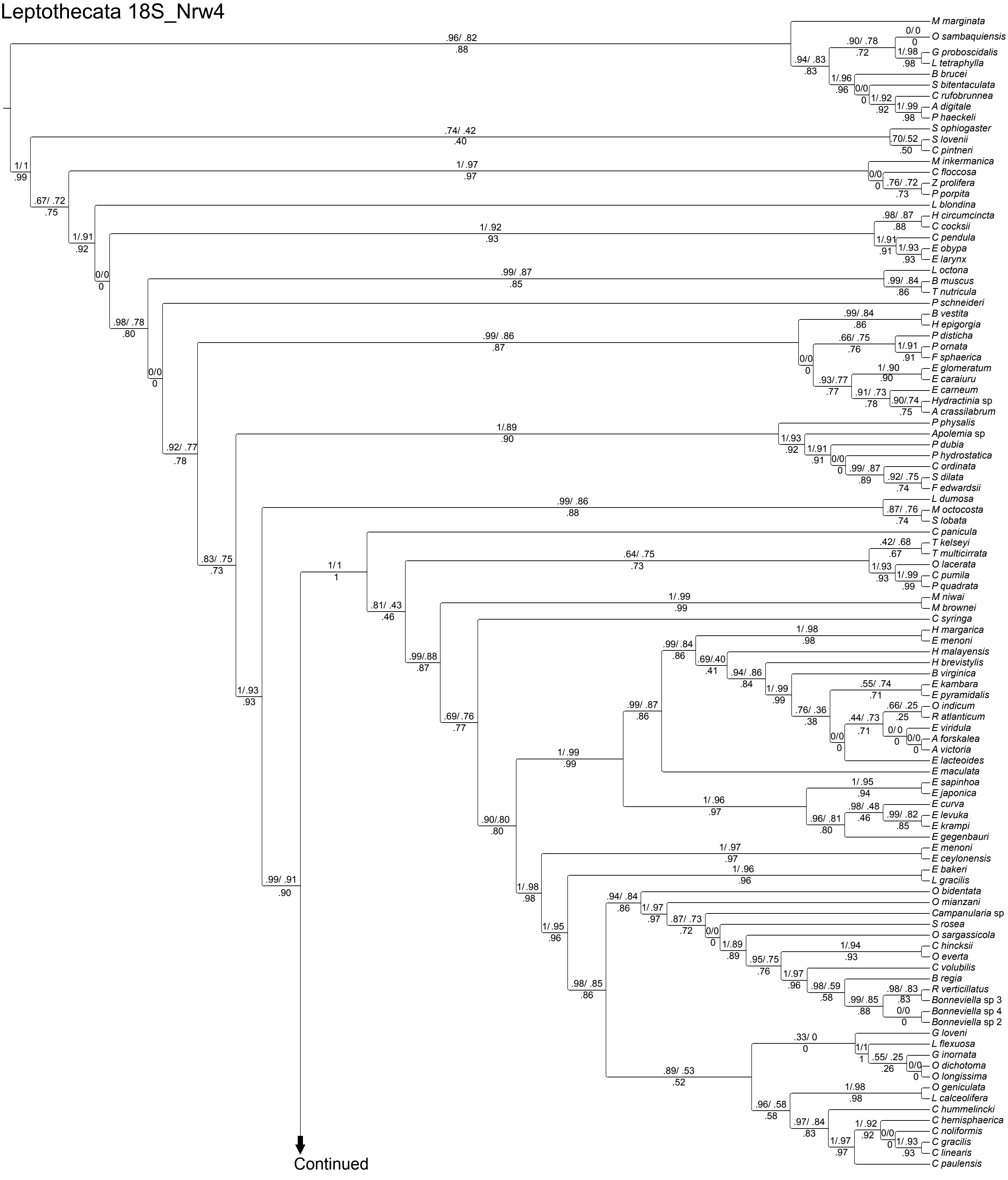


Supplementary Figure S8_A

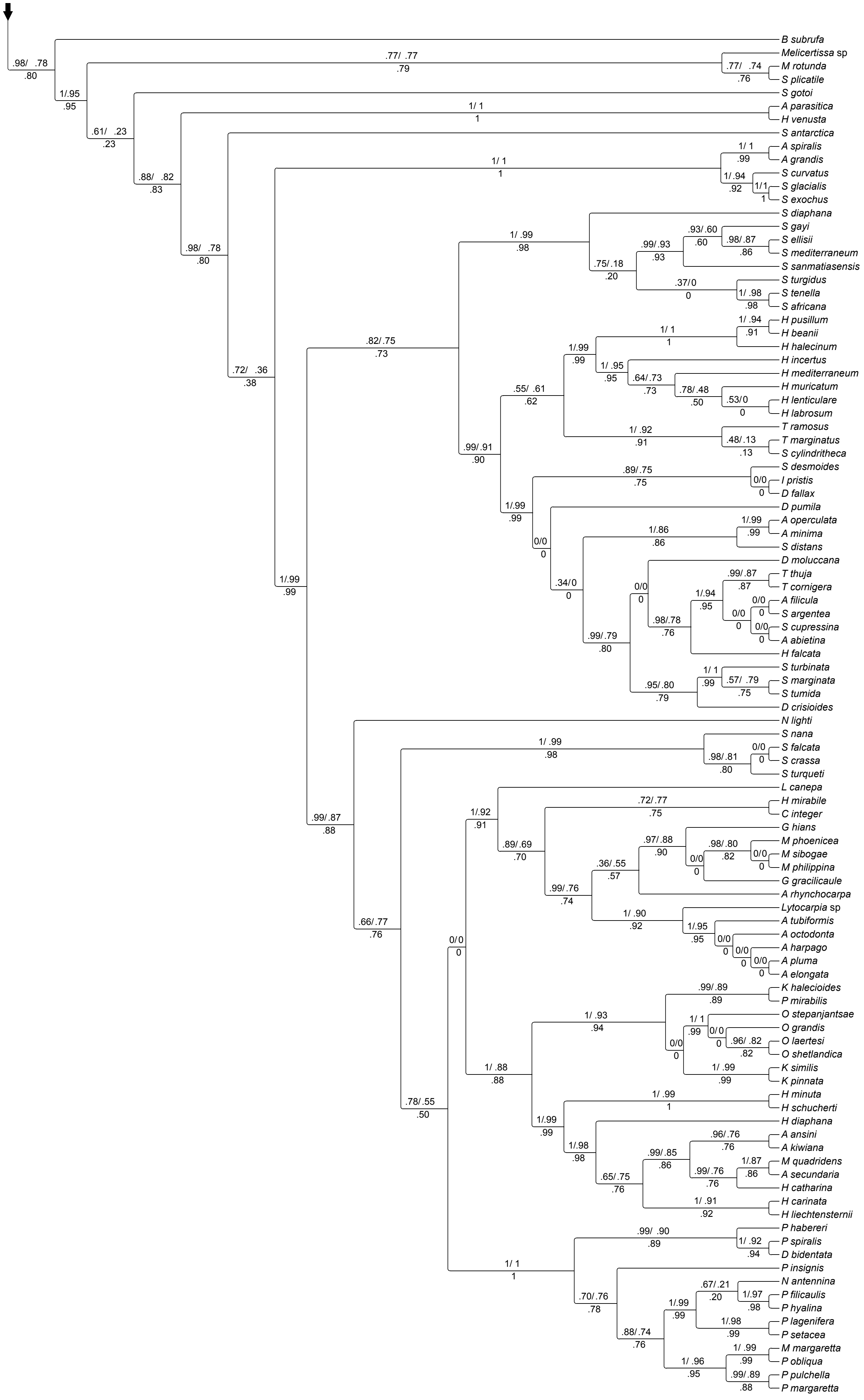


Supplementary Figure S8_B - continued

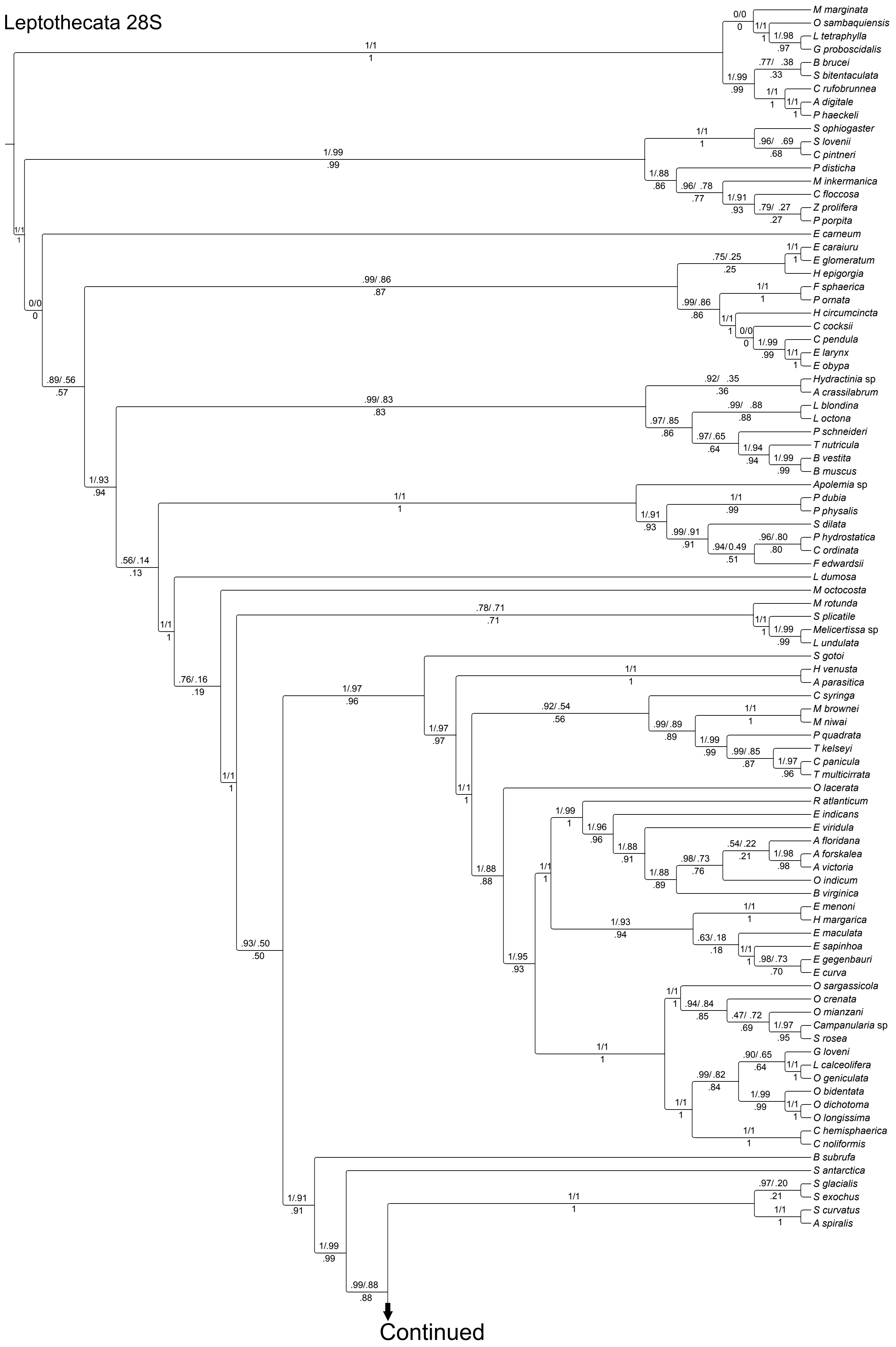


Supplementary Figure S9_A

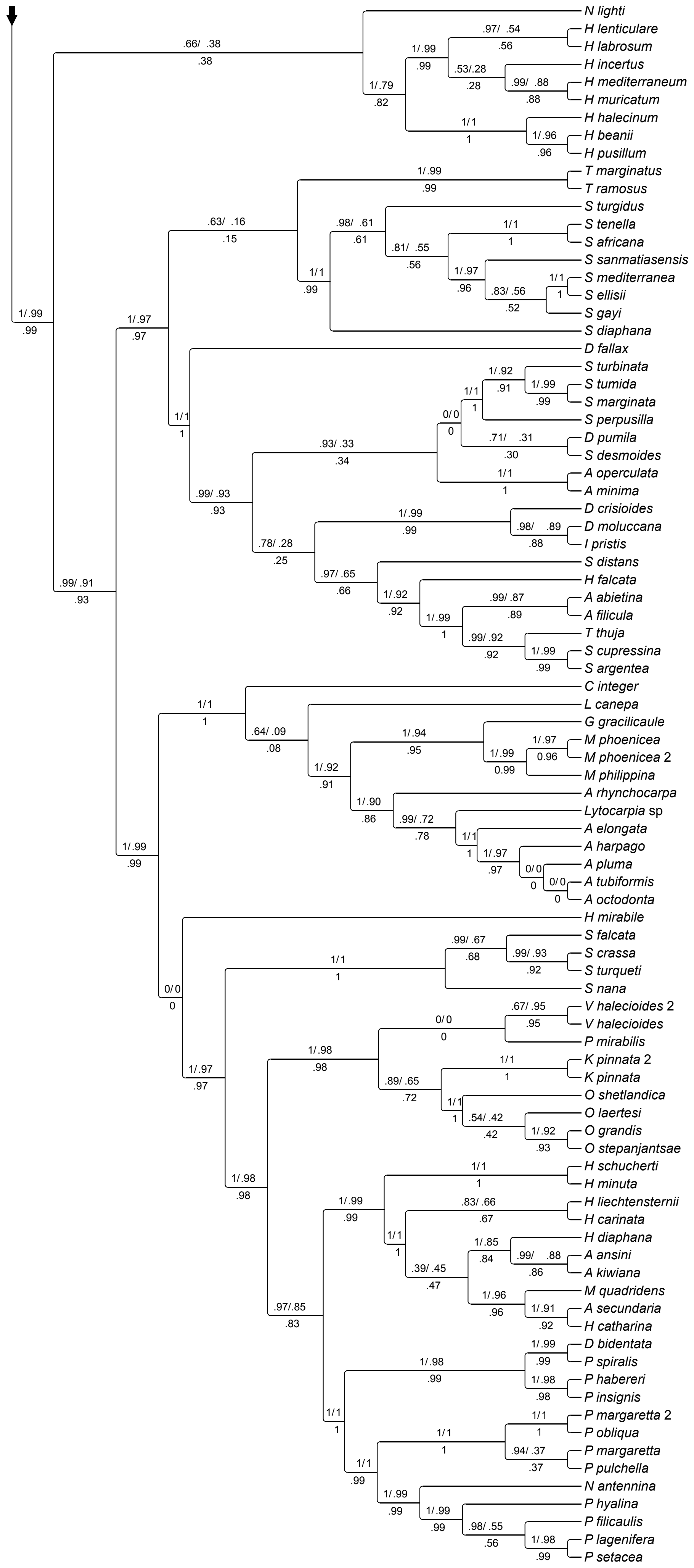


Supplementary Figure S9_B - continued

Leptothecata 28S_N

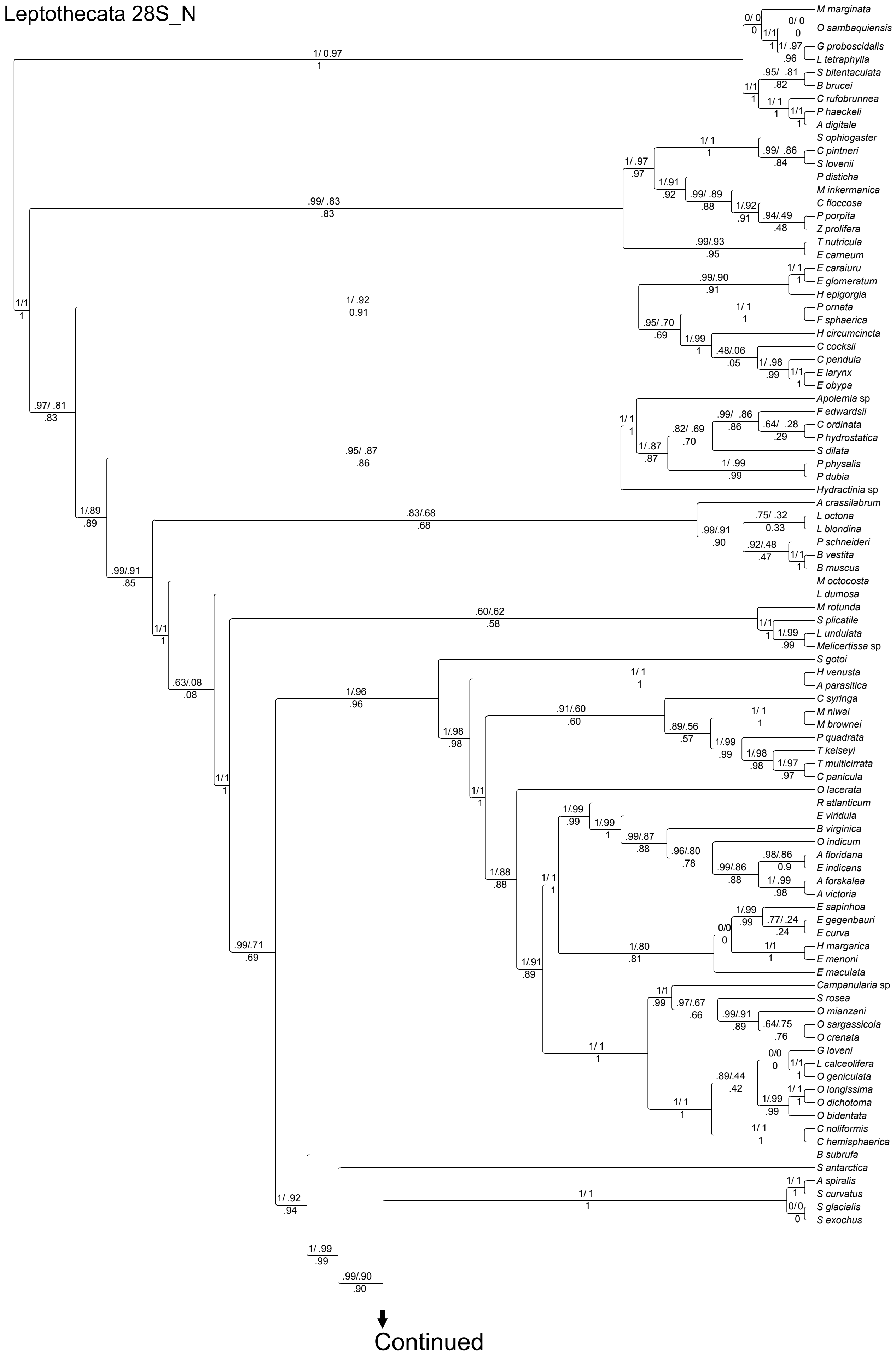


Supplementary figure 10_A

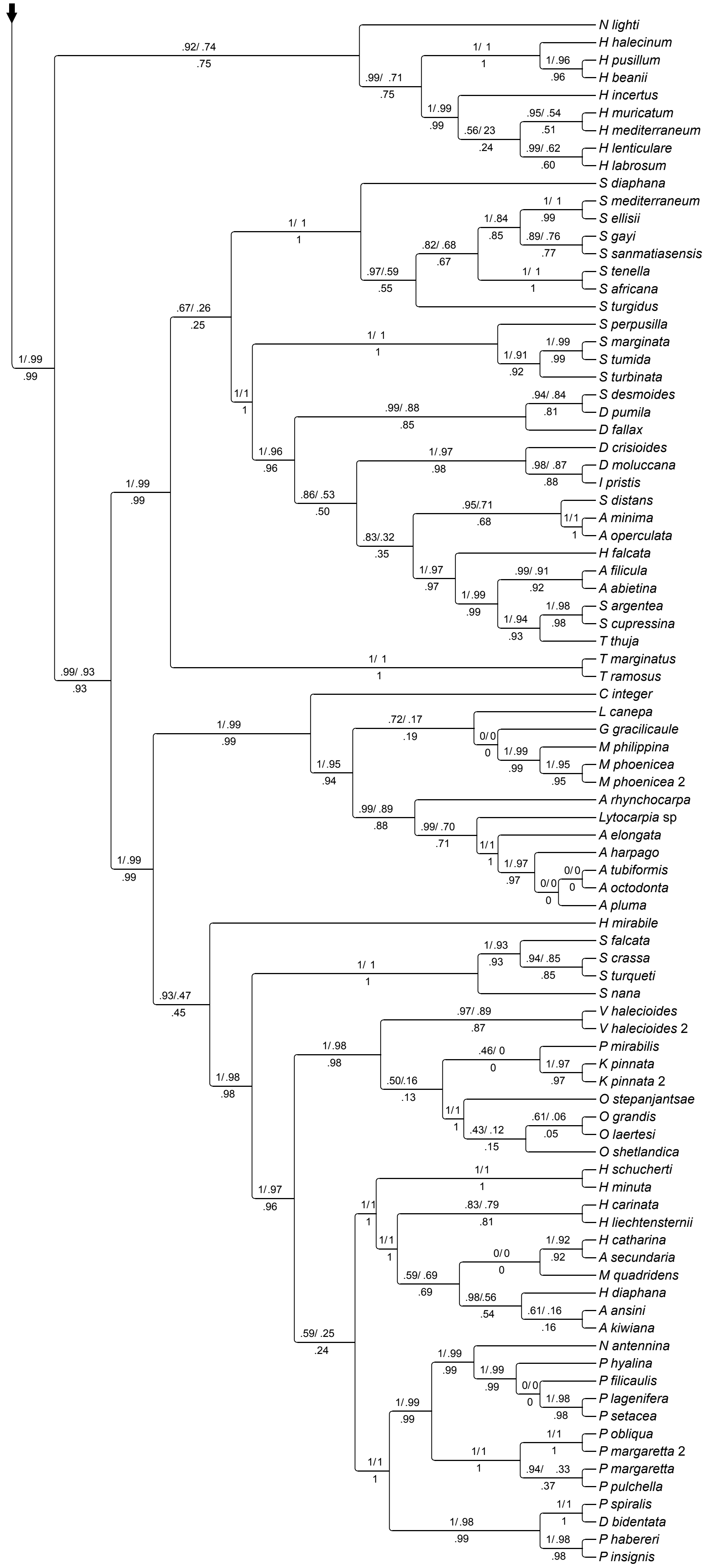


Supplementary Figure S10_B - continued

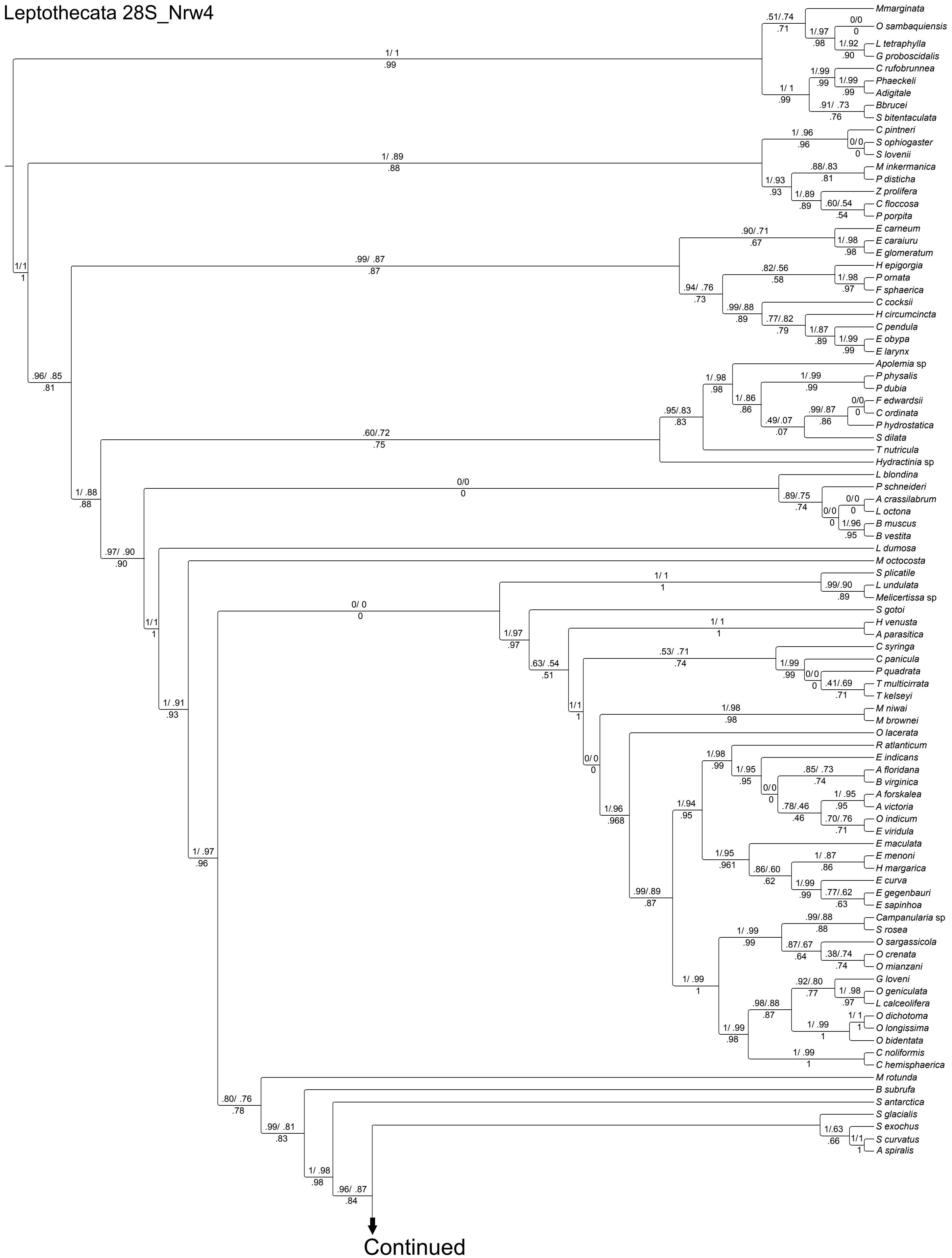


Supplementary Figure S11_A

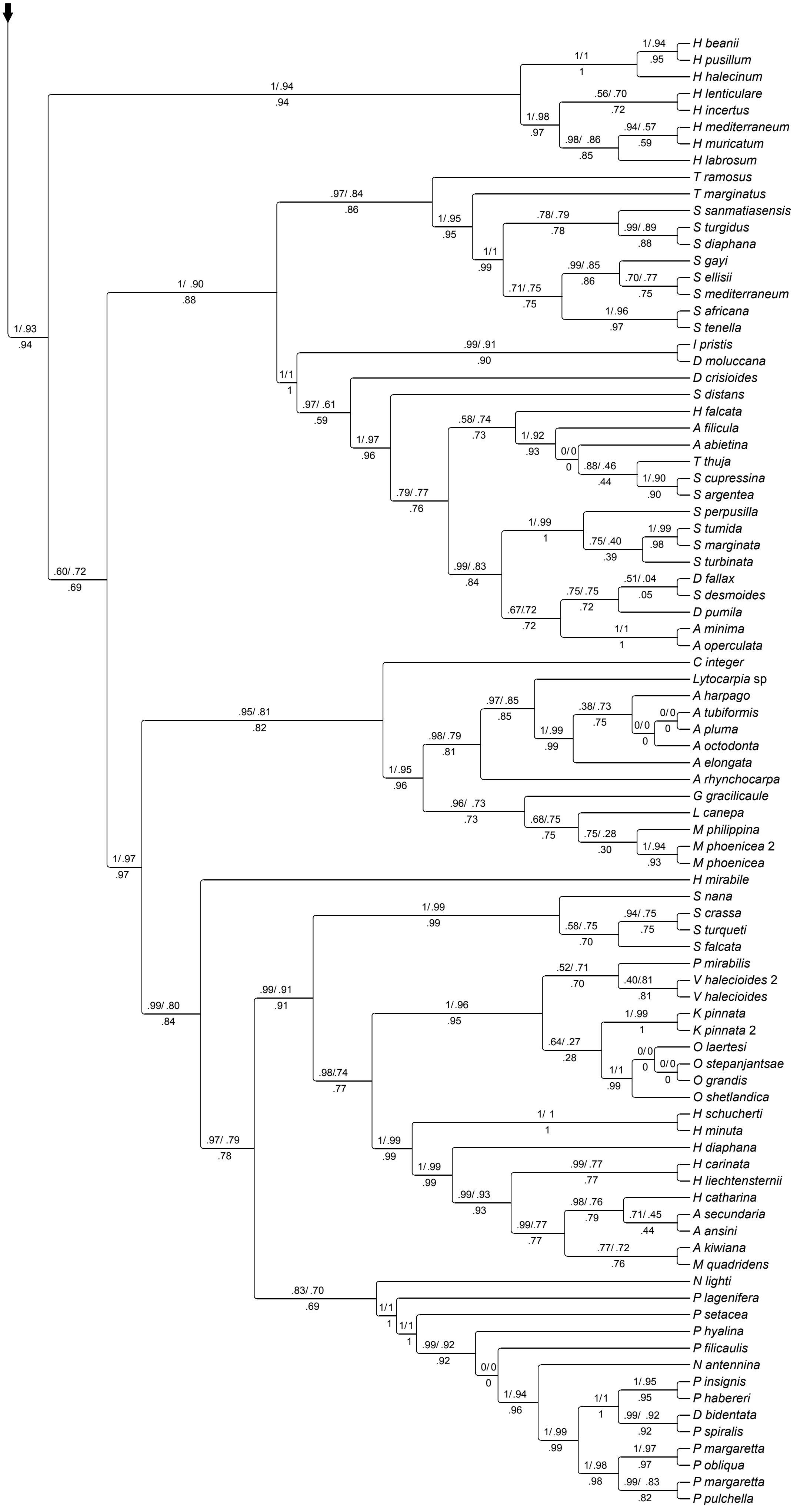


Supplementary Figure S11_B - continued

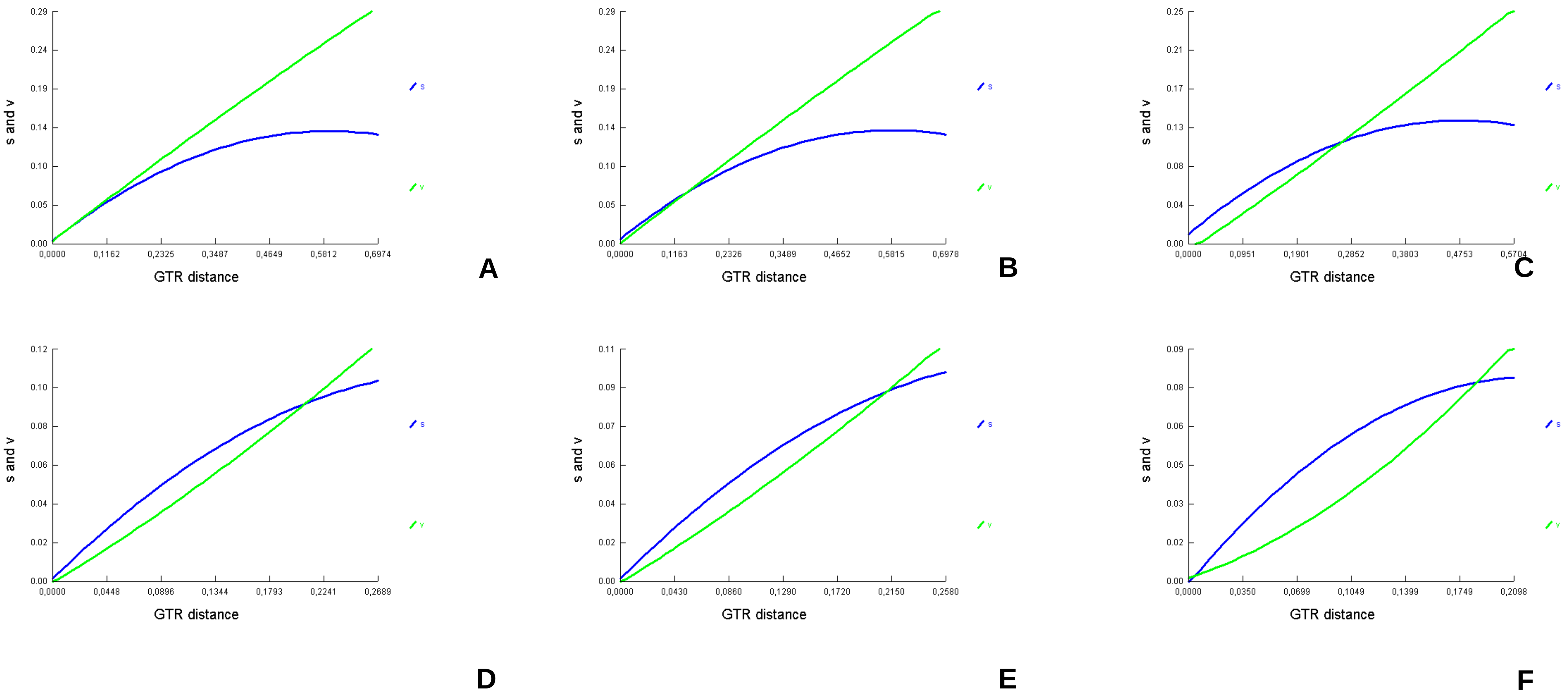


Supplementary Figure 12

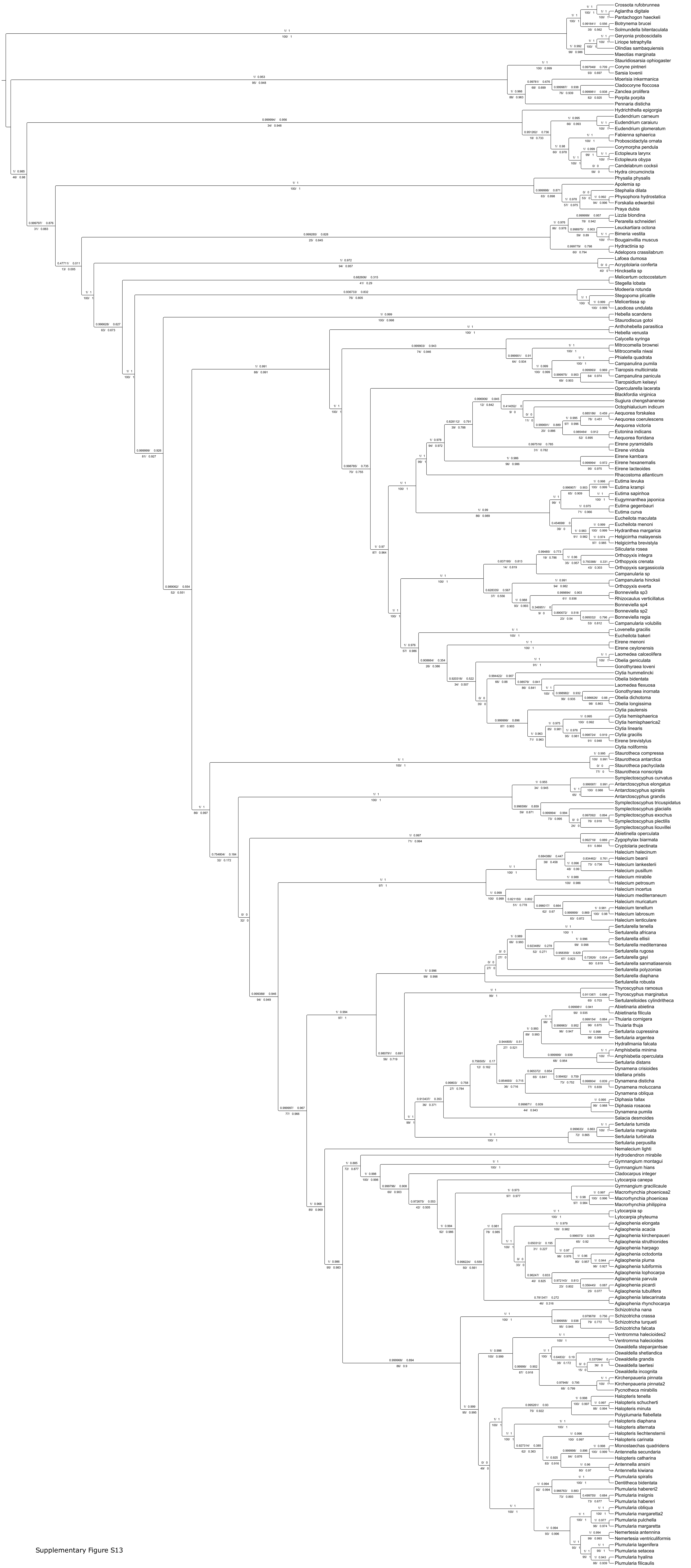

Supplementary Figure S13

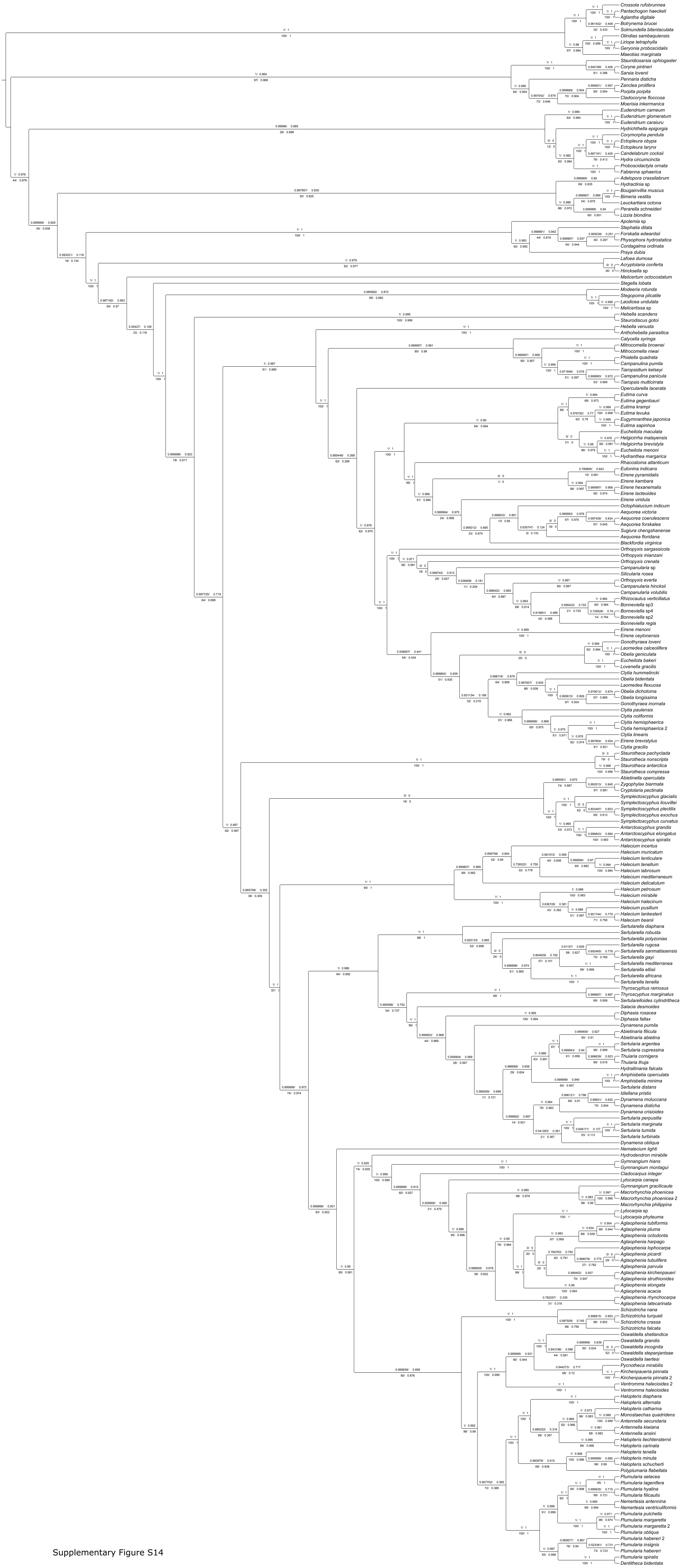

Supplementary Figure S14

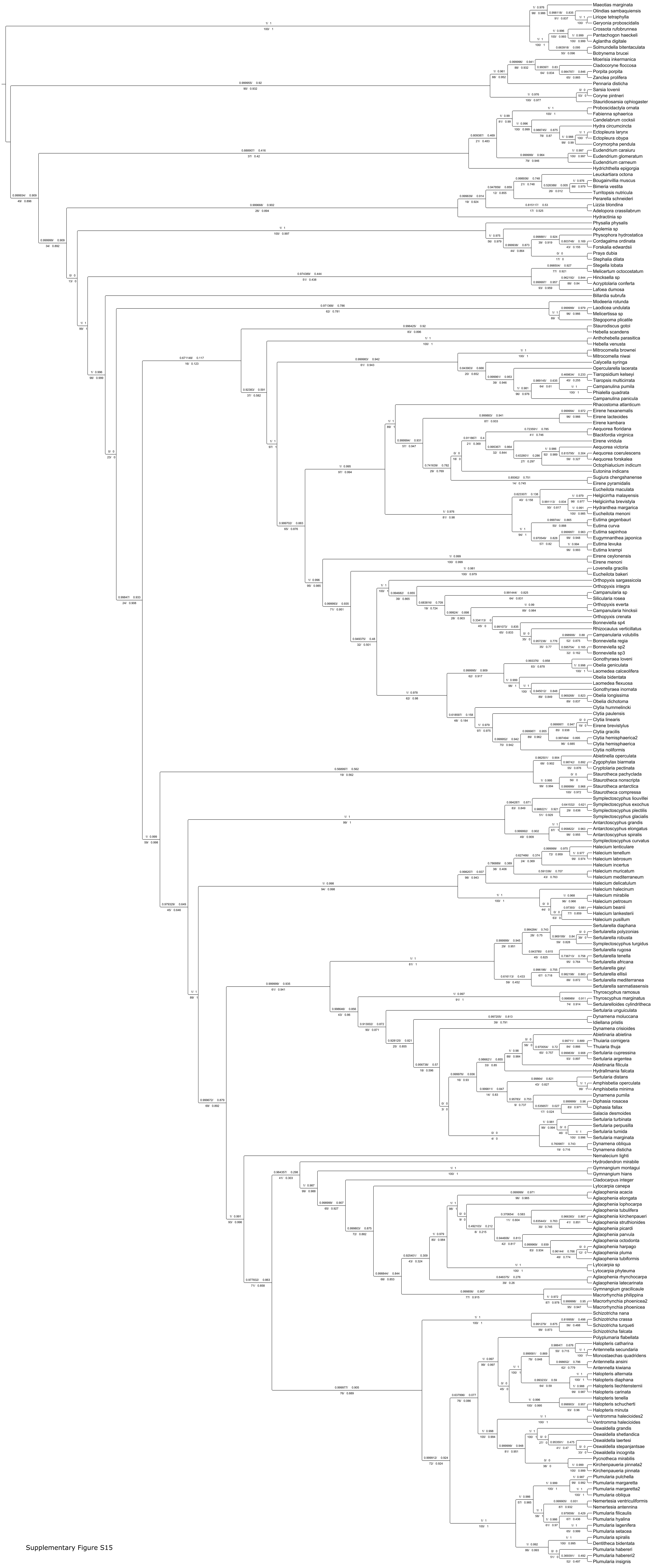

Supplementary Figure S15
